# Haplotype-resolved genome assembly provides insights into the evolutionary origin of waterlogging-tolerant *Actinidia valvata* hexaploid

**DOI:** 10.1093/hr/uhag011

**Published:** 2026-01-09

**Authors:** Feng Zhang, Yunzhi Lin, Yingzhen Wang, Binglong Li, Hongtao Wang, Ying Wu, Yanyan Zhu, Xiuhong Zhou, Wangmei Ren, Lihuan Wang, Ying Yang, Songhu Wang, Junyang Yue, Pengpeng Zheng, Yongsheng Liu

**Affiliations:** School of Horticulture, Anhui Agricultural University, Hefei 230036, China; Department of Horticulture and Landscape Architecture, Taiyuan University, Taiyuan 030032, China; School of Horticulture, Anhui Agricultural University, Hefei 230036, China; School of Forestry Science and Technology, Lishui Vocational and Technical College, Lishui 323000, China; School of Horticulture, Anhui Agricultural University, Hefei 230036, China; School of Horticulture, Anhui Agricultural University, Hefei 230036, China; School of Horticulture, Anhui Agricultural University, Hefei 230036, China; School of Horticulture, Anhui Agricultural University, Hefei 230036, China; School of Horticulture, Anhui Agricultural University, Hefei 230036, China; School of Horticulture, Anhui Agricultural University, Hefei 230036, China; School of Horticulture, Anhui Agricultural University, Hefei 230036, China; School of Horticulture, Anhui Agricultural University, Hefei 230036, China; School of Horticulture, Anhui Agricultural University, Hefei 230036, China; School of Horticulture, Anhui Agricultural University, Hefei 230036, China; School of Horticulture, Anhui Agricultural University, Hefei 230036, China; School of Horticulture, Anhui Agricultural University, Hefei 230036, China; Ministry of Education Key Laboratory for Bio-Resource and Eco-Environment, College of Life Science, State Key Laboratory of Hydraulics and Mountain River Engineering, Sichuan University, Chengdu 610064, China

## Abstract

Kiwifruit plants are much damaged by several days of waterlogging stress. The effect can be a serious problem for the growers in the lowlands or plain areas where floods cannot be drained timely. *Actinidia valvata* is a polyploid species that has been widely used as waterlogging resistant rootstock in kiwifruit cultivation. Here we report haplotype-resolved chromosome-scale assemblies of an *A. valvata* male plant ‘DE’, defining two subgenomes, a diploid closely related to *Actinidia polygama* and an autotetraploid closely related to *Actinidia macrosperma* as their ancestral contributors, respectively, together to form an allohexaploid. Genome-wide comparisons of the assembled 174 pseudochromosomes with nine distinct *Actinidia* species revealed the genomic structure, phylogeny and duplication history of ‘DE’ genome. Evolutionary analyses suggest that it was formed ~0.44 to 0.88 Mya and evolved by a recent alloploid addition to an autotetraploid ancestor. Annotation of sex determining genes (*SyGl* and *FrBy*) on Y chromosome unraveled that the chromosomal location and organization of sex determining region (SDR) are conserved to and share an identical lineage with *A. polygama*, the diploid ancestor. Comprehensive transcriptome analysis indicates that its enhanced waterlogging tolerance is due to the restricted activation of anaerobic survival genes and the consequence with prolonged carbohydrate supply to keep the root system quiescently alive under hypoxia. Our study provides valuable genomic resources and offers insights into the evolution and the underlying mechanism of enhanced waterlogging tolerance of *A. valvata* hexaploid.

## Introduction

Flooding due to extreme climate is a disaster that seriously affects cultivation and productivity of crops. Flooding stress leads to oxygen deprivation and prevents aerobic respiration for ATP synthesis, resulting a severe energy crisis [1, 2]. Due to intermittent soil inundation, terrestrial plants have been evolved to employ anaerobic fermentation as an alternative energy source to cope with the depletion of available oxygen (hypoxia), but this otherwise has led to poor energy metabolic efficiency and accumulating toxic metabolites [3, 4].

The cellular basis for this acclimation is to transcriptionally activating genes coding for critical enzymes of anaerobic fermentation pathway as a consequence of entrapment of the gaseous hormone ethylene and water stress, such as alcohol dehydrogenase (ADH) and pyruvate decarboxylase (PDC) [5, 6]. An analysis of highly resolved chromatin accessibility and gene expression in four distinct angiosperms, ranging from a dryland-adapted wild species to a wetland crop, led to a definition of a cohort of conserved submergence-activated genes with overlapping *cis*-element targeted by four transcription factor (TF) families, including group VII ethylene response factors (ERFVII), bHLH, MYB, and WRKY [7]. Extensive studies demonstrated the ERFVIIs are featured with a destabilizing N terminus, acting as an N-degron that targets them for oxygen-dependent 26S proteosome-mediated degradation, but allowing their stabilization in response to hypoxia stress [2].

Like most fruit trees that are susceptible to waterlogging [8–10], kiwifruit (*Actinidia* ssp.) plants with root system distributed in shallow soil are extremely sensitive to floods [11]. Nevertheless, the waterlogging tolerance of *Actinidia* species exhibits significant genetic diversification [12–14]. An investigation demonstrated that rootstocks from *Actinidia valvata* are much more tolerant to waterlogging condition than those from *Actinidia deliciosa*, which are commonly used in kiwifruit production [13]. Another independent experiment showed *A. deliciosa* plants possess much lower waterlogging tolerance compared to *Actinidia macrosperma*, *A. valvata*, and *Actinidia arguta* [12]. Transcriptome analysis of root tissues from *Actinidia chinensis* and *A. deliciosa* revealed that a batch of most significantly differentially expressed genes (DEGs) responding to waterlogging stress are enriched in glycolytic and anaerobic fermentation pathway [15–17].

Using *A. chinensis* and *A. deliciosa*, gene expression analysis showed that ABA responsive genes, DREB2 and WRKY40, were greatly upregulated in roots with flooding, which are the key site of water stress perception, implicating kiwifruit plants fight against water stress also through regulation of ABA signaling [18]. Several transgenic studies indicated ectopic expression of *AdADH1/2*, *AdPDC1/2*, or *AdRAP2.3* (an AP2/ERF TF) isolated from *A. deliciosa* resulted in enhanced waterlogging resistance [19–21]. Interestingly, transgenic kiwifruit constitutively overexpressing *AcMYB68* and *AcERF74/75* displayed inhibited plant growth and restricted responses to water stress [17, 22].

In recent years, *A. valvata* plant through cuttings has been served as a predominant source of rootstock in kiwifruit production due to its fairly well-adapted floods resistance [12] and robust tolerance to salt stress [23]. However, we still lack information on the genomics and evolutionary origin of its natural polyploid forms for this important *Actinidia* species, primarily because its genome has not yet been sequenced.

Genomic resources are essential for understanding the physiological and environmentally adaptive traits as well as sex determination system. Therefore, in this study we sequenced the genome of a polyploid male of *A. valvata* from a wild population. The haplotype-resolved assembly of all 174 pseudochromosomes unraveled that this individual is indeed a naturally evolved allohexaploid that combines features of autopolyploidy and allopolyploidy. We identified two polyploidization events as the origin of the sexual lineage. Further work concentrates on the relatively high resilience of the *A. valvata* with waterlogging stress. Comparison of its transcriptomic profile with that of susceptible *A. chinensis* counterpart has helped to identify core responsive factors that are critical for survival or for differentially limiting the extent of damage resulting from waterlogging stress. Our results provide first insights into its evolutionary history of the hexaploid and shed light on better understanding of why *A. valvata* plants are well adapted to waterlogging stress.

## Results

### Haplotype-resolved assembly of a hexaploid *A. valvata* genome

**Figure 1:**
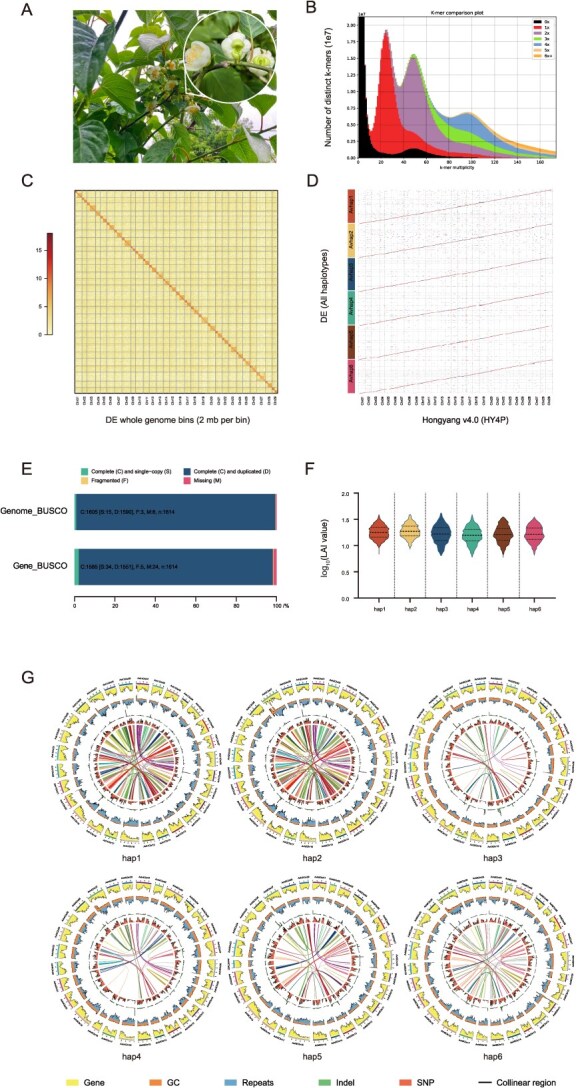
Quality assessment of the DE genome assembly. (A) The plant with flowers, leaves and sepals of *A. valvata* cv. ‘DE’, the number of sepals is usually two. (B) Comparison of the presence of distinct K-mers and copy number variation between the DE assembly and raw HiFi reads. The plots are colored to represent the frequency of specific K-mers derived from the reads present in the assembly. K-mers absent in the assembly are shown as black, while K-mers appearing 1, 2, 3, 4, 5, or ≥6 times are indicated by different symbols or shading. (C) Heatmap displaying Hi-C interaction signals between pseudochromosomes of DE assembly. Each homologous group consists of six pseudochromosomes. (D) Collinearity between the six haplotypes of DE assembly and HY4P. HY4P was used to adjust the orientation of pseudochromosomes of DE assembly. (E) Genome BUSCO and gene BUSCO assessments across all six haplotypes showing proportions classified into categories of complete and single-copy (S), complete and duplicated (D), fragmented (F), and missing (M). (F) Distribution of LAI among the six haplotypic individuals of DE assembly. (G) Genomic features of the six haplotypes of DE assembly. The tracks from outside to inside represents chromosome identifiers (Chr), gene density (Gene), guanine-cytosine content (GC), repeat density (Repeats), InDel density (InDel), SNP density (SNP), and collinear regions, respectively. All statistics were computed for windows of 500 kb.

Flow cytometry was performed to confirm that *A. valvata* cv. ‘DE’ is actually a hexaploid (Fig. S1). For precise genome assembly, the genomic DNA derived from a wild male *A. valvata* accession ‘DE’ (Fig. 1A) was sequenced using the PacBio Sequel II system, resulting in ~173.7 Gb (48.3×) of high-fidelity (HiFi) reads (Table S1). The HiFi reads had an average length of 17.3 kb (Table S1). Additionally, Hi-C libraries were generated using the BGI DNBSEQ-T7 platform, and 394.6 Gb (109.6×) of clean reads were obtained for clustering, ordering, orienting, and unitigs confirming (Table 1 and Table S2). Utilizing the PacBio HiFi and Hi-C data, 26 480 unitigs were assembled via hifiasm [24] program, resulting in 3739.82 Mb with an N50 of 0.84 Mb (Table 1). Subsequently, a slightly modified ALLHiC pipeline [25] was used to phase and assemble these unitigs into pseudochromosomes. Finally, a haplotype-resolved genome was obtained, designated as DE (Table S2).

**Table 1:** Summary statistics of *Actinidia valvata* genome assemblies

**Genomic feature**	**hap1**	**hap2**	**hap3**	**hap4**	**hap5**	**hap6**
Total size of assembled unitigs (Mb)	3739.82					
Number of unitigs	26 480					
N50 value of unitig length (Mb)	0.84					
Total size of assembled genomes (Mb)	549.37	536.98	472.60	467.67	444.30	445.96
Number of pseudochromosomes	29	29	29	29	29	29
Number of telomeres (pairs)	35 (9)	33 (8)	22 (3)	29 (7)	21 (3)	18 (3)
Number of definite centromeres	29	29	29	29	29	29
Genome BUSCOs (%)	99.5					
TE size (%)	40.44	39.13	31.29	32.79	31.06	31.47
GC content (%)	35.85	35.9	34.74	34.75	34.76	34.83
LTR assembly index	16.46	17.53	15.06	14.4	14.99	15.35
Gene BUSCOs (%)	98.2					
Number of genes/transcripts	30 352/36222	29 975/35814	26 322/31633	25 649/30700	25 371/30407	24 892/29903
Number of shared genes	29 640	29 070	25 806	25 184	24 932	24 343
Number of specific genes	712	905	516	465	439	549

The DE assembly comprises six distinct haplotypes, referred to as hap1, hap2, hap3, hap4, hap5, and hap6, each consisting of 29 pseudochromosomes. Six haplotypes span 549.37 Mb, 536.98 Mb, 472.60 Mb, 467.67 Mb, 444.30 Mb, and 445.96 Mb with 584, 577, 731, 728, 714, and 740 gaps, respectively (Table 1 and Table S3).

The accuracy of the six haplotypes was verified through multiple methods. A spectral graph generated by the KAT program [26] provided conclusive evidence that the black region in Fig. 1B corresponds to k-mer containing sequencing errors, which occur only a limited number of times in reads but rarely in the assembled genome. The phase accuracy was also confirmed (Fig. 1B and Fig. S2A). All 174 chromosomes are clustered to 29 homologous chromosome groups and a heatmap was drawn to visualize Hi-C contact signal. Strong Hi-C contact signal can be clearly seen across the diagonal, and the contact falls weak between different homologous chromosome groups. In each homologous chromosome group, the contact signal splits the chromosomes to two blocks. Between the two blocks, Hi-C contact signals are relatively weak, and within them are strong. This validation confirms the accuracy of phasing, ordering, and orientation (Fig. 1C and Fig. S2B). Genome completeness was assessed by aligning various raw read datasets including PacBio HiFi reads, Hi-C reads, and RNA-seq reads to the genome, and their mapping rates were 99.99%, 99.72% and 98.75%, respectively. Collinearity analysis between the six haplotypes of DE and the HY4P reference [27] suggests that they are largely syntenic (Fig. 1D). The assembly completed 99.50% BUSCO gene of the embryophyta_odb10 gene set [28] across all six haplotypes (Fig. 1E and Table 1), confirming a well-assembled *A. valvata* genome. Furthermore, long terminal repeat (LTR) annotations yielded LTR assembly index (LAI) for the six haplotypic individuals with 16.46, 17.35, 15.06, 14.40, 14.99, and 15.35, respectively (Fig. 1F and Table S4), suggesting that DE is a reference-level assembly [29].

Next, 30 353, 29 975, 26 322, 25 649, 25 371, and 24 892 protein-coding genes were annotated for the six haplotypic individuals in the assembly, respectively, capturing 98.2% of the BUSCO embryophyta_odb10 [28] gene set (Table 1). Furthermore, transcript predictions yielded potentially 36 222, 35 814, 31 633, 30 700, 30 407, and 29 903 transcripts with an average of 1.19 to 1.20 alternative splicing variants per gene for the six haplotypic individuals (Fig. 1G and Table 1). Of these protein-coding genes, 30 211, 29 723, 26 242, 25 604, 25 321, and 24 807 were functionally annotated using the comprehensive eggNOG database [30], respectively.

### Structural variations across the haplotypic individuals

**Figure 2:**
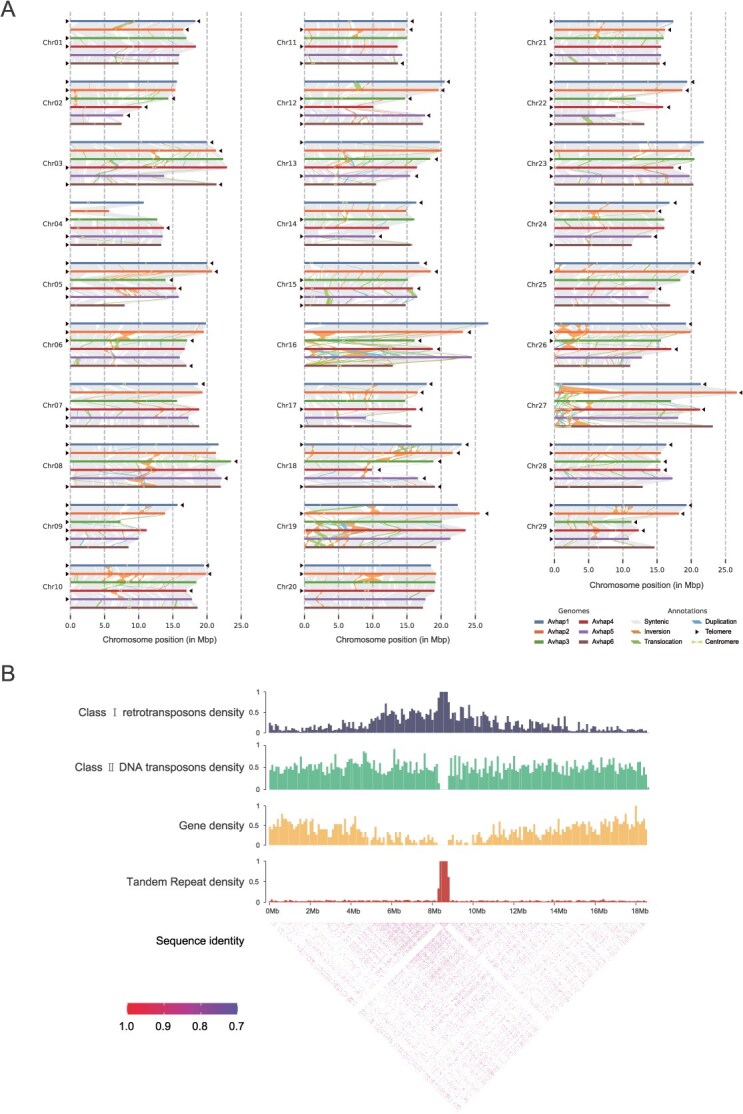
Structural variation analysis of the DE genome. (A) Structure of the haplotype-resolved genome of DE assembly. All 174 chromosomes of the six haplotypes are drawn to scale, and the ruler indicates the length of chromosomes. Collinearity between haplotypes with syntenic regions is represented by gray lines. Inversions, translocations, and duplications are indicated by distinct line types or patterns. Telomeres are marked with black triangles. Predicted centromeres are represented by dumbbell shapes, with their locations and sizes shown. (B) Characterization of the centromere on Chr10 of haplotype 6. The histogram shows the density of Class I retrotransposons, Class II DNA transposons, genes, and tandem repeats density. The heatmap shows the pairwise similarity along the entire chromosome. All features are calculated for window of 50 kb.

A comparative analysis between the haplotypic individuals allowed unraveling a range of genomic attributes, including variations in genome size, the content of repetitive sequences, and gene counts (Table 1). Whole-genome alignments revealed a highly conserved synteny across the main bodies of the annotated haplotypes (Fig. 2A). In comparison with haplotype 1, haplotype 2 ~ 6 displayed different numbers of variations, including 1 897 909, 1 737 799, 1 679 174, 1 632 767, and 1 610 956 single-nucleotide polymorphisms (SNPs); 161 918, 82 063, 77 694, 79 776, and 77 280 insertions; 162 646, 89 513, 84 584, 86 128, and 83 733 deletions; 97, 96, 95, 87, and 89 inversions; 409, 1088, 1190, 1105, and 1180 translocations; as well as 579, 1107, 1083, 1055, and 1120 duplications, respectively (Table S5). The analysis identified 158 975 shared genes that exist in multiple haplotypes distributed among 25 837 orthogroups across the six haplotypes, demonstrating a conserved gene cluster core within the DE assembly (Table 1 and Table S6). In contrast, 571, 653, 436, 420, 389, and 464 unassigned genes that has no homolog in the whole genome were annotated individually in the six haplotypes. (Table 1 and Table S6). These specific genes were validated through mapping against the other haplotype genomes to ensure the specificity.

Next, the relatively conserved potential candidates of telomeres and centromeres were analyzed. By utilizing the TeloExplorer module of quarTeT [31], we detected 35, 33, 22, 29, 21, and 18 distinct telomeres in the six haplotypes of DE assembly and among them, 9, 8, 3, 7, 3, and 3 chromosomes had telomeres at both ends, respectively (Table 1, Fig. 2A, and Table S7). To identify centromeric regions in the DE assembly, we employed the CentroMiner module of quarTeT [31] and found that the majority of centromeres were located near the middle parts of the chromosomes, while a small proportion of centromeres were found at the terminals of chromosomes, including Chr07, Chr19, and Chr27 (Fig. 2A and Table S8). Furthermore, our comprehensive analyses confirmed that it is consistent with previous studies that class I retrotransposons, including Gypsy and Copia, are found in various locations throughout the genome, including traditionally heterochromatic regions such as centromeric, pericentromeric, and subtelomeric regions, as well as class II DNA transposons displayed an even distribution across the genome, while tandem repeats were predominantly located in the centromere region [32, 33]. Chr10 of hap6 was presented as an example (Fig. 2B). These results suggest that the position of centromere is relatively conserved in *Actinidia* species as compared to previous studies [27, 34, 35]. However, it should be noted that our analyses relied solely on bioinformatics predictions without experimental demonstrations, and the precise positions of these centromeres require further validation using more robust techniques, such as ChIP-seq [36].

### Origin of hexaploid *A. valvata*

**Figure 3:**
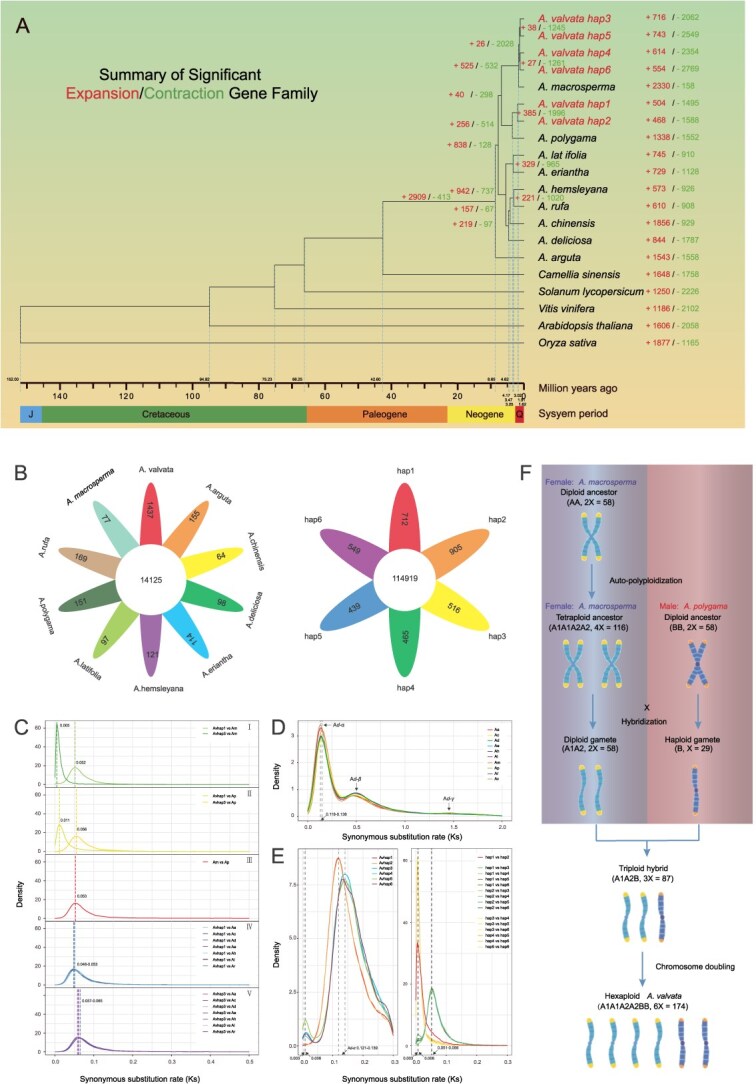
Phylogenetic relationships, genome comparation, and evolutionary analysis of *Actinidia valvata* (Av), *Actinidia arguta* (Aa), *Actinidia chinensis* (Ac), *Actinidia deliciosa* (Ad), *Actinidia eriantha* (Ae), *Actinidia hemsleyana* (Ah), *Actinidia latifolia* (Al), *Actinidia macrosperma* (Am), *Actinidia polygama* (Ap), *Actinidia rufa* (Ar), and other 5 outgroup species. (A) Phylogenetic trees of gene family expansion/contraction and divergence time based on orthologous genes. (B) Petal maps of genes in species-specific and haplotype-specific orthogroups. (C) *K_S_* distribution of orthologous gene pairs among (I) Av and Am, (II) Av and Ap, (III) Am and Ap, (IV) Av hap1 and other *Actinidia* species, and (V) Av hap3 and other Actinidia species. (D) *K_S_* distribution of paralogous gene pairs among 10 Actinidia species. (E) Haplotype-aware *K_S_* distribution of paralogous and allelic gene pairs in *A. valvata*. The left panel represents *K_S_* distribution of paralogous gene pairs, and the right panel shows that of allelic gene pairs. (F) Evolutionary model of the hybrid origin and WGD history of *A. valvata*.

Utilizing Orthofinder [37] and r8s [38], we inferred the phylogenetic relationships and divergence times for *A. valvata* ‘DE’ and fourteen other plant species (Fig. 3A), including *A. arguta* (Aa) [39], *A. chinensis* (Ac) [27], *A. deliciosa* (Ad) [40], *A. eriantha* (Ae) [34], *A. hemsleyana* (Ah) [41], *A. latifolia* (Al) [42], *A. macrosperma* (Am) [43], *A. polygama (*Ap) [39], *A. rufa* (Ar) [39], as well as non-*Actinidia* species, i.e. *Camellia sinensis* [44], *Solanum lycopersicum* [45], *Vitis vinifera* [46], *Arabidopsis thaliana* [47], and *Oryza sativa* [48]. The six haplotypes derived from the *A. valvata* can be divided into two distinct subgenomes. Subgenome A consisting of two haplotypes (hap1 and hap2) is further clustered and shares a closest evolutionary lineage with *A. polygama*. The remaining four haplotypes (hap3, hap4, hap5, and hap6) constitute subgenome B that are further clustered and share a closest evolutionary lineage with *A. macrosperma* to form an adjacent parallel distinct clade that represents a potential tetraploid ancestor of the hexaploid *A. valvata*. The constructed phylogenetic tree (Fig. 3A) and the Hi-C heatmap (Fig. S2B) suggests the sequenced *A. valvata* ‘DE’ is an allopolyploid containing two distinct subgenomes, a diploid (subgenome A) closely related to *A. polygama* and an autotetraploid (subgenome B) closely related to *A. macrosperma*, hereafter designated as their putative ancestral contributors.

To characterize the Y chromosome of the male *A. valvata* ‘DE’, a BLASTp homology search [49] was performed to locate sex-determining region (SDR) using the protein sequences of sex-determining genes, the *SyGl* and *FrBy* located in SDR, from *Actinidia* species [50]. Highly conserved with *A. polygama* [39], the chromosomal position and organization of SDR encompassing *SyGl* and *FrBy* genes were exclusively determined on chromosome 25 in hap1 (Fig. 2A and Fig. S2C). To infer whether the absence of SDR in haplotypes other than hap1 is due to true biological absence or a consequence of collapse, we further validated the mapping depth of SDR region, and the result showed that there is no trace of collapse in SDR region as the mapping depth is largely identical to the sequencing depth (Fig. S3), suggesting that the SDR region is indeed single copied in *A. valvata*.

Next, we investigated the expansion and contraction of gene families. In the annotated *A. valvata* genome, two subgenomes are diversified with significant expansion or contraction of 40 or 298 gene families, respectively (Fig. 3A). Functional enrichment analysis of expanded gene families revealed enrichment in multiple biological processes and molecular functions that could be relevant to stress adaptation (Fig. S4A). The enrichment of negative regulation of phosphoprotein phosphatase activity, phosphatase inhibitor activity, and related phosphatase regulatory pathways indicate a potential role in sustaining phosphorylation-dependent signaling processes that have been implicated in hypoxia-responsive regulation [51]. Enrichments of casparian strip and secondary cell wall metabolisms are correlated with possible modifications to apoplastic barriers that may influence ion and water homeostasis under high-moisture conditions [52–54]. KEGG enrichment in ascorbate and aldarate metabolism refer to involvement in reactive oxygen species (ROS) detoxification pathways [55]. Additional expansions in aminoacyl-tRNA biosynthesis and cytoplasmic translation are shown to be related to the maintenance of protein synthesis under stress [56], while phospholipid:diacylglycerol acyltransferase activity confers membrane lipid remodeling under stress conditions [57]. In contrast, contracted gene families related to developmental polarity regulations (e.g. polarity specification of adaxial/abaxial axis, adaxial/abaxial axis specification, adaxial/abaxial pattern specification, specification of axis polarity) suggest a reduced investment in organ morphogenesis, allowing energy to be redirected toward survival under prolonged hypoxic stress condition [58] (Fig. S4B). Similarly, the reduction of metabolic pathways such as complex carbohydrate catabolism (polysaccharide digestion, digestion) and nucleotide dephosphorylation (polynucleotide 5′ dephosphorylation) implicates a downregulation of non-essential energy-intensive processes, potentially conserving carbohydrate reserves during submergence [59]. In addition, the contraction of immune-related processes (neutrophil activation involved in immune response, myeloid leukocyte mediated immunity, and neutrophil mediated immunity) may reflect a strategic shift from costly defense activities toward core physiological functions that are favorable for oxygen management and cellular stability [60]. Collectively, these patterns indicate coordinated genomic reprogramming during the evolution of *A. valvata*, where expansions bolster stress signaling, ROS detoxification, and structural reinforcement, while contractions streamline growth, secondary metabolism, and energy-intensive pathways. This combination of enhanced defense-related capacities and reduced non-essential functions may together underpin the species’ notable waterlogging tolerance.

Additionally, all the nine sequenced genomes of *Actinidia* species share 14 125 orthogroups, whereas *A. valvata* possesses 1437 species-specific orthogroups that may represent an important determinator distinguishing *A. valvata* from other *Actinidia* species (Fig. 3B). Furthermore, the six haplotypic individuals differentially contain 712, 905, 516, 465, 439, and 549 genes in haplotype-specific orthogroups, respectively (Table 1 and Fig. 3B).

Followed using JCVI program [61], 369 945 orthologous pairs between the representative subgenomic haplotypes (hap1 or hap3) of *A. valvata* ‘DE’ and nine other *Actinidia* species were identified. Estimations of synonymous substitution rates (*K_S_*) demonstrated that the divergence of *Actinidia* species intensively occurred 7.1 ~ 9.6 Mya, and that the two distinct subgenomes in *A. valvata* ‘DE’ are supposed to be diverged from their ancestors 0.7 ~ 1.8 Mya after the species divergence (Fig. 3C, Tables S9 and S10). This analysis is basically in accordance with the lineage revealed by the phylogenetic tree construction. In addition, *K_S_* values calculated for a total of 137 227 paralogous gene pairs within ten monoploid genome of *Actinidia* species suggest two recent whole-genome duplication (WGD) events, occurring ~16.8 ~ 20.6 Mya (*Ad-α*) and ~ 73.7 Mya (*Ad-β*), respectively (Fig. 3D, Tables S9 and S11). Meanwhile, we identified 10 987, 10 953, 9577, 8768, 8932, and 8220 paralogous gene pairs within the six individual haplotypes, respectively. Paralogous pairs derived from hap1 or hap2 are weakly peaked at 0.003, suggesting a most recent WGD event (designated as WGDh) occurred about 0.44 Mya. By contrast, paralogous pairs derived from hap3, hap4, hap5 or hap6 are abundantly peaked at 0.006, suggesting a recent WGD event (designated as WGDt) preceding WGDh occurred ~0.88 Mya after *Ad-α* (Fig. 3E left panel, Tables S9 and S12).

Concurrently, allele-specific annotation allowed identification of allelic genes on homologous chromosomes within the DE assembly. Using the JCVI program [61], 7023, 7372, 7845, 6005, and 8383 loci were found with sextuple, quintuple, quadruple, triple, and double alleles in all the 162 561 genes annotated, respectively (Table S13). Meanwhile, 17 402 genes with no allele found across the six haplotypes were denoted as ‘singletons’ in this study. *K_S_* values for allelic gene pairs between hap1 and hap2 are intensively peaked at 0.003, consistently implicating the most recent WGD event (WGDh) occurred 0.44 Mya (red color graph in Fig. 3E right panel, Table S9). *K_S_* values for allelic gene pairs across hap3, hap4, hap5, and hap6 are acutely peaked at 0.003 ~ 0.006, suggesting multiple WGD events might happen 0.44 ~ 0.88 Mya (yellow color graph in Fig. 3E right panel, Table S9). *K_S_* values for allelic pairs between the two subgenomic haplotypes (i.e. between hap1/2 and hap3/4/5/6) display two distinct peaks (green color graph in Fig. 3E right panel, Table S9). The right peak covers a *K_S_* distribution region ranging from 0.051 to 0.056, which probably represents the divergency time of the two subgenomic ancestral species, estimated to have occurred about 7.52 ~ 8.26 Mya. And the left peak encompasses a *K_S_* distribution region ranging from 0.003 ~ 0.006, reflecting two recent WGD events might occur 0.44 ~ 0.88 Mya. Taken together, *K_S_* estimates of both paralogous pairs and allelic pairs suggest that the sequenced *A. valvata* ‘DE’ genome comprises of two profoundly differentiated (7.52 ~ 8.26 Mya) subgenomes, a diploid (subgenome A) highly homologous to *A. polygama* and a tetraploid (subgenome B) highly homologous to *A. macrosperma*, wherein the tetraploid ancestor was formed about 0.88 Mya and the hexaploidization occurred about 0.44 Mya.

In *Actinidia* species, chloroplast is uniparentally inherited from paternal plant [62]. To infer the directionality of hybridization in polyploid formation, chloroplast genome of *A. valvata* ‘DE’ was assembled utilizing Oatk software [63]. Combined with seven other *Actinidia* species chloroplast genomes including *A. polygama*, *A. macrosperma*, *A. arguta*, *A. eriantha*, *A. chinensis*, *A. deliciosa*, and *A. kolomikta*, a phylogenetic tree was constructed using *A. thaliana* chloroplast genome as outgroup. The result showed that the chloroplast genome of *A. valvata* ‘DE’ is most closely clustered with that of *A. polygama*, suggesting that ancestors of *A. polygama* may serve as the paternal donor in the hybrid origin of polyploid (Fig. S5). In summary, we could infer that a hybridization event between the ancestral female tetraploid and male diploid initially led to the formation of a triploid hybrid, which subsequently underwent chromosome doubling to evolve into the hexaploid *A. valvata* (Fig. 3F).

### Allele specific expression underlies the subgenomic dominance

*Actinidia chinensis* ‘Hongyang’ and *A. valvata* ‘DE’ showed distinct responses to waterlogging stress. To evaluate phenotypic alterations between ‘Hongyang’ and ‘DE’, waterlogging treatments were applied. The results showed that ‘Hongyang’ plants subjected to 3 days of waterlogging displayed wrinkled leaves and a detectable reduction in root number compared with the control group (CK), suggesting the growth of ‘Hongyang’ was markedly inhibited under waterlogging stress. In contrast, ‘DE’ showed no obvious changes in growth status (Fig. S6A). Further assessment of root activity through TTC (2,3,5-Triphenyltetrazolium chloride) staining [64] revealed pronounced fading of the red staining in ‘Hongyang’ after 3 days of waterlogging, whereas ‘DE’ roots retained a clear red coloration (Fig. S6B), suggesting higher root activity in ‘DE’ under waterlogging stress. Notably, even in the control group, the root activity of ‘DE’ is higher than that of ‘Hongyang’ (Fig. S6B). Quantitative measurements of root activity supported the staining results, showing that root activity in ‘Hongyang’ (HY) was significantly reduced after 3 days of waterlogging, while that of ‘DE’ remained almost unaffected (Fig. S7).

WGD is a major driver of gene functional divergence and transcriptional reprogramming, shaping the evolution of key agronomic traits. WGD has been shown to contribute to improvements in quality traits [65, 66] and multiple stress tolerances, including lodginge [67], disease [68, 69], cold [70], drought [71, 72], and salinity tolerance [73, 74]. An important outcome of WGD is the generation of multiple homoeologous haplotypes, which frequently display regulatory divergence and subgenome-specific expression bias. In order to explore the subgenomic dominance, RNA-seq was conducted on roots of ‘DE’ plants subjected to different durations of waterlogging treatments (0, 1, 2, 3 days after flooding). Investigation into the expression level for genes with six alleles showed that the total expression was unbalanced among the six haplotypes, and the expressed transcripts derived from hap1 to hap2 were lower than that from other four haplotypes (Fig. S8A), suggesting that subgenomic dominance existed in the annotated A. *valvata* assemblies. Leveraging the assembled haplotypes and RNA-seq data, we explored the divergence in gene sequences and the expression imbalance among allelic genes. For the 7023 loci with six alleles, an analysis of 12 RNA-seq datasets revealed that 6233 loci (88.75%) exhibited a distinct allele-specific expression (ASE) pattern in response to different waterlogging treatments (|log2FoldChange| > 1 and *P* < 0.05), demonstrating that the expression of most sextuple-allelic genes was unbalanced (Fig. S8B and Table S14). All those genes that displayed switched expression dominance between alleles at different waterlogging time points were identified as inconsistent ASE genes (ASEGs), in accordance with expression patterns reported in rice. Based on the RNA-seq dataset, we calculated the mean expression levels of 6951 genes with sextuple alleles for hap1/2 and hap3/4/5/6 haplotype groups, respectively. The results showed that across different time points after waterlogging stress treatment, 4170 ~ 4223 genes exhibited preferential expression in hap3 ~ hap6, while 2723 ~ 2770 genes were biased toward hap1 ~ hap2 (Fig. S8C). Similarly, the expression fold-change (log_2_(hap3 ~ 6/hap1 ~ hap2)) frequency distribution of these genes further demonstrated that the more genes exhibited expression bias toward hap3 ~ hap6 (Fig. S8D). Consistently, BUSCO analysis of the genome and genes showed that the dominant subgenome displays a higher level of BUSCO completeness (Fig. S9). Specifically, ERF gene family that has been shown to play pivotal role in waterlogging resistance [22, 75–79] was carefully inspected in the genome of *A. valvata* ‘DE.’ A total of 1149 ERF family members were annotated throughout the six haplotypes (Fig. S10). At 58 loci with six alleles of ERF manifested a distinct ASE pattern in response to different periods of waterlogging stress treatments, in which the gene expression levels of hap1/2 were lower than those of the other four haplotypes (hap3/4/5/6) with an overall consistent expression at different time points (Fig. S11). In addition, ASEGs of all six haplotypes were mainly enriched in the regulation of chlorophyll biosynthetic/metabolic processes (GO:0010380/GO:0090056) and the regulation of tetrapyrrole biosynthetic process (GO:1901463) (Fig. S12A and Table S15). Furthermore, Kyoto Encyclopedia of Genes and Genomes (KEGG) pathway annotations suggested that ASEGs are functionally enriched in several biological pathways, including glycine, serine and threonine (ko00260) and tyrosine (ko00350) metabolisms as well as photosynthesis (ko00195) (Fig. S12B and Table S16).

### Identification of waterlogging resistance genes

To elucidate the mechanism underlying the much stronger flooding resistance observed in *A. valvata* than that in *A. chinensis* [12–14, 22], we compared and analyzed the DEGs between the transcriptomes derived from the root tissues of *A. chinensis* cultivar ‘Hongyang’ (‘HY’) [27] and the annotated *A. valvata* ‘DE’ at 0, 1, 2, and 3 days after flooding treatment using the assembled haplotypic genomes. Sample clustering analysis revealed significant differences between two genotypes of *A. chinensis* and *A. valvata* at days 0, 1, 2, and 3 following flooding treatment, with the three biological replicates in each treatment clustering closely, suggesting the high quality of the RNA-seq libraries (Fig. S13A).

**Figure 4:**
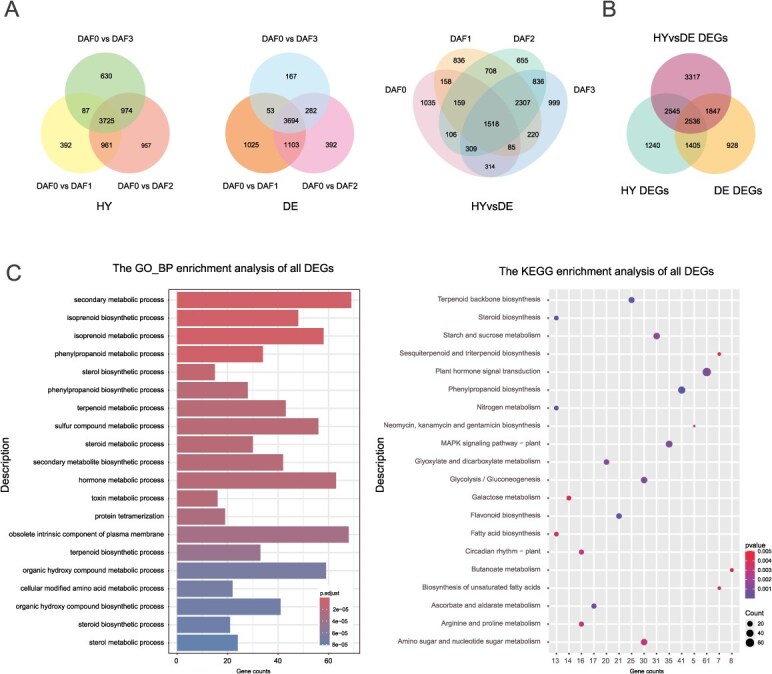
DEGs analysis in *A. chinensis* cv. ‘Hongyang’ and *Actinidia valvata* cv. ‘DE’ at 0, 1, 2, or 3 days after root flooding (DAF). (A) Venn diagram of the DEGs of *A. chinensis* (HY, left panel) and *A. valvata* (DE, middle panel) under different waterlogging treatment time, and the DEGs between *A. chinensis* and *A. valvata* at the same flooding stage (HYvsDE, right panel). (B) Venn diagram of the DEGs in HY, DE and HYvsDE. (C) GO and KEGG pathways enrichment analysis of the DEGs in HY, DE, and HYvsDE.

We analyzed the DEGs within individual genotypes under different durations with flooding (DAF0 vs DAF1, DAF0 vs DAF2, DAF0 vs DAF3). A total of 7726 DEGs (|log2FoldChange| > 1, *P* < 0.05) were individually identified in *A. chinensis* ‘HY,’ of which 3725 genes were differentially expressed throughout the three treatments (Fig. 4A left panel). In *A. valvata* ‘DE’, 6716 DEGs were individually identified, of which 3694 genes exhibited differential expression across the three durations of treatments (Fig. 4A middle panel). Importantly, 3941 DEGs were identified simultaneously in both *A. chinensis* and *A. valvata* genotypes. In addition, a total of 10 245 DEGs between the two different genotypes (HY vs DE at DAF0, DAF1, DAF2, DAF3) were identified at the same time point of flooding treatment (Fig. 4A right panel). These may be the genes responsible for allowing *A. chinensis* ‘HY’ and *A. valvata* ‘DE’ expressing differential responses with flooding. Among all the DEGs identified, 2536 were simultaneously present and overlapped by either distinct genotypes or different durations of flooding stress treatment (Fig. 4B). GO enrichment analysis showed that these DEGs were mainly enriched in secondary metabolic process (GO:0019748), hormone metabolic process (GO:0042445), and toxin metabolic process (GO:0009404) (Fig. 4C left panel and Table S17). In addition, changes in key metabolic pathways under different waterlogging stress treatments were investigated through KEGG pathway enrichment analysis. The resultant DEGs were enriched in 112 pathways, such as Plant Hormone Signal Transduction (ko04075), Phenylpropanoid Biosynthesis (ko00940), MAPK Signaling Pathway - Plant (ko04016), Starch and Sucrose Metabolism (ko00500), and Glycolysis/Gluconeogenesis (ko00010) (Fig. 4C right panel and Table S18). These results unraveled that a number of enriched DEGs are homologous to previously characterized waterlogging/hypoxia responsive genes, such as *ACO* [80], *CAT* [81], *ADH* [82], *PDC* [19], *RBOH* [83], *PCO* [84, 85], *XTH* [86], *CIPK* [87, 88], *GASA* [89], *GST* [90], *PYL* [91], *TPS* [16], *SUS* [92], and *HUP* [93], as well as several TF families including ERF, WRKY and MYB [7]. Interestingly, NAC TF family is also enriched while not previously reported to involve waterlogging/hypoxia response.

Next, we compared the expression pattern of the core waterlogging responsive genes between the tested genotypes (Fig. S14). As a result, ethylene synthesis enzyme gene *ACO1* and hypoxia response unknow protein *HUP54* were significantly upregulated by waterlogging stress in both *A. chinensis* and *A. valvata*. Interestingly, the expression levels of candidate genes, such as catalase *CAT2*, gibberellin-regulated protein *GASA1*, plant cysteine oxidase *PCO2*, and abscisic acid receptor *PYL* that are beneficial for detoxification or survival with flooding are significantly higher in *A. valvata* ‘DE’ than in *A. chinensis* ‘HY.’ By contrast, the expression of genes related to alcoholic fermentation (*TPS11*, *ADH1*, and *PDC1*), generation of reactive oxygen species (*RBOHD*), and cell wall modification (*XTH23*) were relatively lower in the *A. valvata* ‘DE’ than *in A. chinensis* ‘HY.’

**Figure 5:**
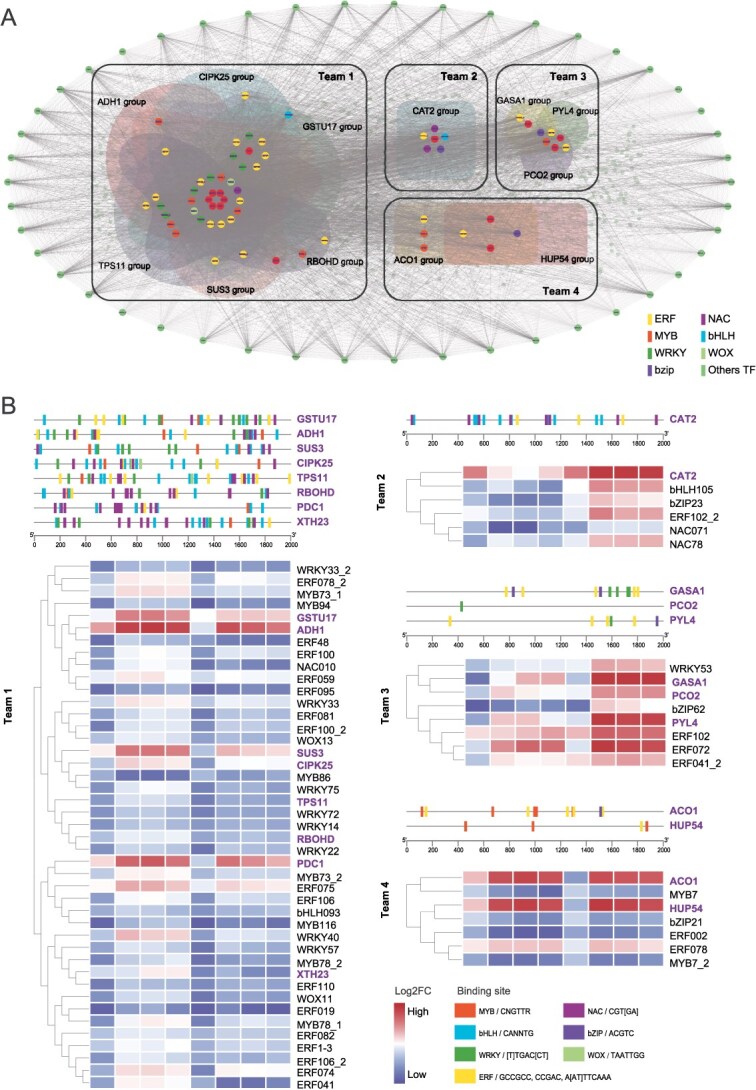
Weighted gene-coexpression network analysis (WGCNA) of *Actinidia chinensis* cv. ‘Hongyang’ and *Actinidia valvata* cv. ‘DE’ at 0, 1, 2, or 3 days after root flooding (DAF). (A) Network visualization of waterlogging related genes regulating flood tolerance. (B) Analysis of the promoters of the hub genes in the positive network and the expression pattern of the candidate genes or transcript factors screened by WGCNA.

Subsequently, weighted gene co-expression network analysis (WGCNA) was conducted for the RNA-seq libraries derived from *A. chinensis* ‘HY’ and *A. valvata* ‘DE’, and the resulting regulatory network was associated with the screened DEGs between the two genotypes with flooding to explore the regulatory cascades of waterlogging tolerance (Fig. 5A and Table S19). Seven modules were identified upon the analysis, and each module showed a different correlation with the expression of the waterlogging tolerance gene set, among which the MEblue module was generally highly correlated (Fig. S13B). Furthermore, we screened for gene sets with correlation coefficient higher than 0.85. Utilizing eggNOG and NCBI BLAST annotations, we identified 110 genes and TFs associated with waterlogging tolerance. To further investigate the regulatory relationship of these genes, we extracted the sequence of 2000 bp upstream of the coding region as the promoter region and search for *cis*-acting elements using PlantPan [94]. Subsequently, we visualized the interaction network of these genes using Cytoscape [95] (Fig. 5A). The network and upset diagram revealed numerous intersections among different groups, particularly with *ADH1*, *CPIK25*, *GSTU17*, *TPS11*, *RBOHD*, *SUS3*, and the TFs (including WRKY, ERF, NAC and MYB) involving multiple groups. Subsequently, by integrating the information across various groups, these groups were merged into teams (Fig. 5A and B and Fig. S15). Transcriptome expression level clustering was performed on the genes of different teams using the Pearson correlation coefficient method. Furthermore, by integrating the TF binding sites and transcriptome profile, many regulatory relationships between TFs and their direct targets were determined (Fig. S16 and Table S20). For instance, anaerobic respiration pathway genes, such as *ADH1* (in Team 1), exhibit a close clustering relationship with ERF48, ERF100, ERF059, ERF95, and NAC010. Additionally, ADH1 contains multiple cis-acting elements, which may allow it to be transcriptionally activated by these TFs (Fig. 5B). It is notable that the expression of these hub genes expression levels in ‘DE’ was significantly lower than in ‘HY’ (Fig. 5B), suggesting the compromised activation of alcohol fermentation pathway underlying the waterlogging tolerance in the *A. valvata*. It worth noting that, in addition to ERF, bHLH, WRKY, and MYB TFs that have been previously demonstrated to directly regulate the expression of hypoxia responsive genes [7, 17, 22], NAC TF is initially implicated by our analysis involving the adaptability of waterlogging responses. Quantitative real-time PCR (qRT-PCR) was performed to further validate the expression of NAC010 and other four representative genes (homologs of ADH1, PDC1, MYB68, ERF74) putatively involved in response to waterlogging stress [17, 22]. As shown in Fig. S17, qRT-PCR results were consistent with the RNA sequencing data.

**Figure 6:**
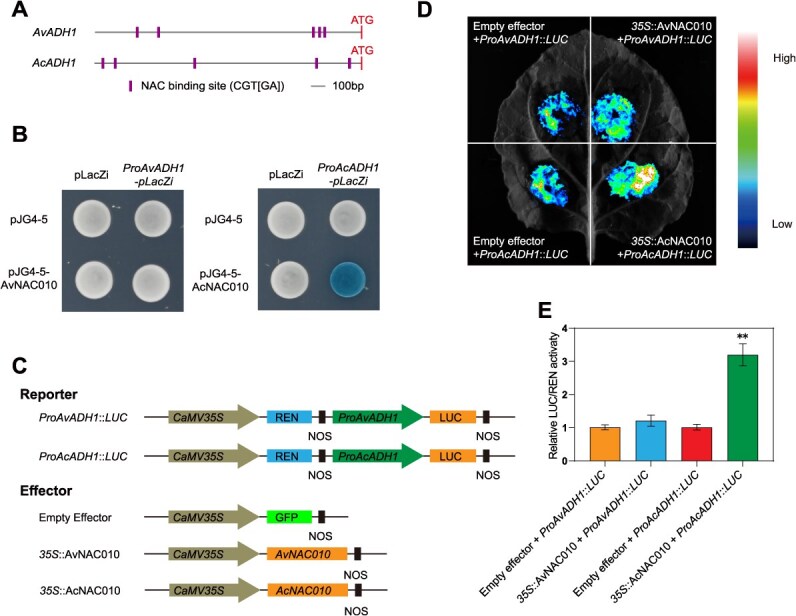
NAC010 can directly bind to the promoter of ADH1 and activates its transcription. (A) Schematic diagram of the promoter regions of *AcADH1* and *AvADH1.* The upstream regions of the start codon (ATG) are indicated by a gray line. (B) Yeast one-hybrid assays showed that AcNAC010 directly binds to the promoter of *AcADH1*, and AvNAC010 cannot binds to the promoter of *AvADH1*. The yeast cells co-expressing the indicated plasmid pairs were plated on SD/-Trp/-Ura medium containing raffinose, galactose, and X-gal. (C) Schematic diagrams of the reporter and effector vectors utilized in the dual-luciferase reporter assay. (D) Dual-luciferase reporter assays indicated that AcNAC010 could transcriptionally activate the expression of *AcADH1*, while AvNAC010 shows no significant activation or inhibition effect on *AvADH1*. The *Agrobacterium* GV3101 harboring the indicated plasmids was co-injected into *Nicotiana benthamiana* leaves, and images were captured 36 to 48 h after infiltration. (E) The relative LUC/REN ratio confirmed that AcNAC010 enhanced the expression of *AcADH1*. Data are presented as means ± SD of three independent experiments. Asterisks represent significant differences (^**^P < 0.01, Student’s *t*-test).

The NAC TF was reported to recognize the NAC binding sites (CGT[GA]) present in the promoters of target genes [96]. Promoter analysis using PlantPans [97] showed that there are five NAC binding sites within the promoter regions located 2000 bp upstream of the coding sequences (CDS) of *AcADH1* and *AvADH1*, respectively (Fig. 6A), suggesting that AcNAC010 or AvNAC010 may activate *AcADH1* or *AvADH1* expression by directly binding to the *cis*-elements (Fig. 5B). To verify this possibility, the promoter regions of *AcADH1* and *AvADH1* were isolated and cloned into pLacZi vector to generate *ProAcADH1*-*pLacZi* and *ProAvADH1*-*pLacZi* constructs, respectively. Subsequently, the yeast one-hybrid (Y1H) assay was conducted. As shown in Fig. 6B, the interaction between AcNAC010 or AvNAC010 and the promoter of *AcADH1* or *AvADH1* was tested by selective medium (-Trp/-Ura/+Gal/+Raf/+X-Gal) culturing. Blue-white screening showed that AcNAC010 was able to bind the specific regions on the *AcADH1* promoter at the NAC binding sites, but not in the negative control pLacZi. However, the interaction signal was not detected between AvNAC010 and the promoter region of *AvADH1* (Fig. 6B). To further investigate the impact of NAC TF on the activity of the *ADH1* promoters, we performed a dual-luciferase (LUC) reporter assay in *Nicotiana benthamiana* leaves. The *AcADH1* and *AvADH1* promoters were cloned into the upstream of the firefly luciferase (LUC) reporter gene to construct the reporter vectors (*ProAcADH1*::*LUC* and *ProAvADH1*::*LUC*) (Fig. 6C). Each vector was subsequently co-transfected with either an empty vector or a *35S*::*AcNAC010* or a *35S*::*AvNAC010* effector vector. Although an addition of either AcNAC010 or AvNAC010 could enhance the fluorescence intensity via activating their own promoters using the empty vector as control, much stronger activation signal was observed in *AcNAC010*/*ProAcADH1*::*LUC* combination than that in *AvNAC010*/*ProAvADH1*::*LUC* (Fig. 6D and E). There was no significant enhancement effect between the experimental group 35S::*AvNAC010* + *ProAvADH1*::LUC and the control group Empty effector+*ProAvADH1*::LUC. Collectively, these results suggest that NAC TF is able to bind to the promoter of hypoxia-responsive *ADH1* gene and directly activate its expression.

## Discussion

Our study presents the first haplotype-resolved chromosome-scale assembly of a male allohexaploid *A. valvata* plant that has been predominantly used as rootstocks. We provided comprehensive genomic data and analysis demonstrating the *A. valvata* genome is a hexaploid constituting two distinct subgenomes, an autotetraploid highly homologous to *A. macrosperma* and a diploid highly homologous to *A. polygama* (Fig. 3A and C). Our detailed annotation of sex-determining genes (*SyGl* and *FrBy*) (Fig. S2C) indicates that a sex-determining region (SDR) was identified only in hap1 of the hexaploid genome and resided at the same location on the same chromosome (Chr25) as *A. polygama* and *A. chinensis* [27, 39]. Significantly, the arrangement of *SyGl* and *FrBy* as well as SDR orientation in *A. valvata* ‘DE’ are identical to the *A. polygama*, but just opposite to *A. chinensis* (Fig. S2C). Consistently, we additionally sequenced and assembled the chloroplast genome of the *A. valvata* ‘DE’ and constructed a phylogenetic tree showing that its chloroplast genome is most closely clustered with that of *A. polygama*, further suggesting that *A. polygama* is the potential paternal ancestor (Fig. S5). These analyses together infers that the sequenced *A. valvata* individual could be evolved from chromosome doubling/WGD of an initial triploid hybrid derived from an ancestral autotetraploid female (subgenome B) cross-pollinated by a distinct diploid male (subgenome A) (Fig. 3F). This hypothesis is supported by several previous investigations. The chloroplast of kiwifruit is inherited paternally. Phylogenetic relationship reconstruction using 29 independent chloroplast genomes’ sequences derived from 25 *Actinidia* taxa demonstrated that *A. valvata* shares the closest lineage with *A. polygama* [98], suggesting the chloroplast genome of our sequenced *A. valvata* ‘DE’ was inherited from *A. polygama*, i.e. the male ancestor. Paternal mode of chloroplast inheritance was first reported in the cultivated hexaploid species *A. deliciosa* by using diverse SSR markers [99], and subsequently this mode was described in many other *Actinidia* species including *A. polygama* [100, 101]. In fact, the natural geographical distribution areas of *A. valvata* and the two potential ancestral species (*A. polygama* and *A. macrosperma*) defined in present study have a large overlapping distribution area in the middle and lower reaches of the Yangtze River in China [102] (Fig. S18), indicating that hybridization events between the ancestors are possible. Evidence was provided to show that interspecific hybridization is common among plants of the genus *Actinidia*, and the complex dynamics of gene flow have formed a pattern of reticulate evolution [103]. Nevertheless, a concatenated tree constructed using genome-wide SNPs derived from second-generation sequencing data showed *A. kolomikta* was clustered with *A. macrosperma* to form an independent branch [103], raising an issue of which of these two species is more closely related to our *A. valvata* ‘DE’ assemblies. By integrating the second-generation sequencing data of *A. kolomikta* and *A. macrosperma* [103], we reconstructed a phylogenetic tree (Fig. S19) showing subgenome B from *A. valvata* ‘DE’ was clustered with *A. macrosperma*, instead of *A. kolomikta*, further suggesting *A. macrosperma* is more likely the female tetraploid ancestor of *A. valvata* ‘DE.’

Calculations of *K_S_* values for 137 227 paralogous gene pairs suggest two recent WGD events occurred ~16.8 ~ 20.6 Mya (A*d-α*) and ~ 73.7 Mya (A*d-β*), respectively (Fig. 3D). These findings well align with previous investigations in *A. chinensis* [104, 105], *A. deliciosa* [40] and *A. arguta* [35]. *K_S_* estimates for either allelic gene pairs or paralogous gene pairs derived from the six haplotypes suggest that the time for tetraploidization of the *A. macrosperma* ancestor is about 0.88 Mya (proximately close to that of tetraploid *A. arguta* [35]) and the time for hexaploid formation is about 0.44 Mya (Fig. 3E right panel).

We also provide evidence showing that the deploidization of allopolyploid at the level of either subgenome size or individual genes occurs in an equilibrated manner. Large variations in genome size and genes content were observed within or between the two distinct subgenomes (Fig. 3A, C and Fig. S2B). The genome size and genes count of the four haplotypes that make up the tetraploid ancestor are apparently smaller than that of the two haplotypes that constitute the diploid ancestor (Table 1), suggesting the genome of tetraploid ancestors began to condense before the formation of the hexaploid. In addition, within subgenome A (containing hap1 and hap2), the entire SDR including *SyGl* and *FrBy* was absent in hap2, probably due to male gametes containing a single copy of SDR were in equilibrium and had a developmentally competitive advantage over those containing redundant SDR during meiosis of microspore mother cells and fertilization (Fig. S2C). We postulated that hap2 initially had SDR during hexaploidization but got lost by abnormal meiosis usually happened in polyploid reproduction or by deletion mediated through transposase [39, 50]. To test this hypothesis, we reassembled a haplotype-resolved male *A. polygama* genome utilizing the data with accession number of DRR393523 [39] and obtained phased (X and Y) genomic sequences on chromosome 25. Comparison in the genomic structures of chromosome 25 between *A. valvata* hap2 and *A. polygama* indicated that hap2 was largely syntenic with Y chromosome (instead of X chromosome) of *A. polygama* except the missing SDR, and multiple inversions were detected between *A. polygama* X chromosome and *A. valvata* hap2 (Fig. S20). Considering that the inversions could inhibit recombination between X and Y chromosomes, the *A. valvata* hap2 without SDR is not likely to originate from an ancestral X chromosome of *A. polygama*.

In addition, our transcriptome analysis showed that both the tested genotypes of *A. chinensis* ‘HY’ and *A. valvata* ‘DE’ were evolved into rapidly responding to waterlogging stress by activating expression of genes key to anaerobic metabolism and flooding survival (Fig. 4), although *A. chinensis* has been characterized as susceptible and *A. valvata* as resistant [12–14]. It is worth noting that the expression of anaerobic respiratory pathway genes, such as *trehalose-6- phosphate synthase* (*TPS*), *PDC*, and *ADH*, and their direct upstream TFs, such as ERF, MYB, WRKY, and NAC, was significantly increased with waterlogging stress in ‘HY,’ while they were just slightly triggered by the stress and subsided during the prolonged flooding in ‘DE’ (Fig. 5). TFs including ERF, bHLH, WRKY, and MYB have been previously demonstrated to directly activate expression of hypoxia responsive genes [7, 17, 22]. In our study, we discovered that NAC TFs might also play a significant role in regulating waterlogging-responsive genes, thereby expanding the adaptive regulatory network of plant roots under flooding stress. Using yeast one-hybrid (Y1H) and luciferase reporter (LUC) assays, we demonstrated that AcNAC010 was able to strongly activate the expression of *AcADH1* and may cooperate with the characterized regulators like ERF, WRKY, and MYB to form a complex transcriptional regulatory network (Fig. 6B and D). These observations are consistent with previous investigations showing that the *A. valvata* confers relatively strong tolerance to floods by compromised changes in physiological activities through a moderate increase in ADH activity and ethanol level. In contrast, the ethanol content in *A. chinensis* increases significantly, with a marked enhancement in ADH activity, leading to excessive ethanol accumulation in the roots and resulting in plant death [7, 22]. Moreover, the enhanced waterlogging resistance observed in *A. macrosperma*, the defined subgenomic tetraploid ancestor in this study, is comparable to or even exceeds that of *A. valvata* [12]. This suggests that the flooding tolerance conferred by *A. valvata* ‘DE’ may largely derive from its autotetraploid *A. macrosperma* ancestry.

## Materials and methods

### Plant materials, library preparation and DNA sequencing

Cuttings of *A. valvata* cv. ‘DE’ was collected and cultured in tissue culture incubators at Anhui Agricultural University in Anhui Province, China, at a temperature of 25°C with a 12-h light and 12-h dark cycle. Fresh and healthy leaves were harvested from three-week-old branches, rapidly frozen in liquid nitrogen, and then stored at −80°C for subsequent PacBio HiFi and Hi-C sequencing. A modified version of the cetyltrimethylammonium bromide (CTAB) technique [106] was used to extract high molecular weight genomic DNA (gDNA) from each individual leaf sample. An Agilent 2100 Bioanalyzer (Agilent Technologies, CA, USA) and a Qubit fluorometer (Thermo Fisher Scientific, MA, USA) were utilized to evaluate the purity and concentration of the extracted gDNA, respectively. Following the manufacturer’s guidelines, a SMRTbell library for PacBio HiFi sequencing was constructed using 50 μg of gDNA and the SMRTbell Express Template Prep Kit 2.0. The library was sequenced on the PacBio Sequel II platform (Pacific Biosciences, CA, USA). The preparation and sequencing of the Hi-C library followed a standardized procedure previously described in the literature [107].

### Assembly and evaluation of the genome

The sequencing output from the PacBio Sequel II system was processed using the SMRT Analysis software suite (version 5.1.0; available at https://www.pacb.com/products-and-services/analytical-software/smart-analysis/). The CCS subprogram (accessible at https://github.com/PacificBiosciences/ccs) was used with default settings to generate consensus HiFi reads. Unitig level assembly was obtained using hifiasm (v0.16.1) [24] software utilizing HiFi and Hi-C reads with default parameters. Then the unitigs were phased into six haplotypes following a modified ALLHiC (v0.9.13) [25] pipeline as follows. Hi-C reads were mapped to the unitigs using BWA (v0.7.17) [108] and samtools (v1.15.1) [109] software, then the alignments were filtered using ‘PreprocessSAMs.pl’ and ‘filterBAM_forHiC.pl’ script given in ALLHiC pipeline. Genes on the unitigs were identified by GMAP (v2021-12-17) [110] software referring to the primary haplotype of *Actinidia chinensis* diploid T2T genome [27]. The allelic unitigs are identified using script ‘gmap2AlleleTable.py,’ a minorly adjusted script of ‘gmap2AlleleTable.pl’ given in ALLHiC pipeline for compatibility with annotation format. For unitigs with no ortholog gene found, minimap2 (v2.24) [111] software was used to align them with reference genome. These unitigs were assigned to the best-matched chromosomes utilizing custom script ‘globle_rescue_alt.py,’ acting as an alternative of ALLHiC global rescue. After that, using script ‘partition_gmap.py’ given in ALLHiC pipeline, the unitigs and Hi-C alignments are split into 29 chromosome groups to reduce the complexity of large genome. For unitigs in each chromosome group, ‘ALLHiC_prune’ were applied to remove the Hi-C contact signal between allelic unitigs. Then the unitigs with strong Hi-C contact signal were clustered into haplotype groups using ‘allhic partition.’ To address the issue that ‘ALLHiC_rescue’ works not well for polyploid kiwifruits, we made an alternative method ‘rescue_alt.py’ working as follows. The minimap2 software was used to generate pairwise alignment between unitigs. If two unitigs in a haplotype group have large overlaps, the one with less Hi-C link density will be removed from this group. After that, all ungrouped unitigs were tested with in-group unitigs in order of Hi-C contact signal from strong to weak. If the tested ungrouped unitig has no large overlap between any unitigs in that group, it will be placed in that group. Next, the order and orientation of unitigs in each haplotype group were adjusted using ‘allhic optimize,’ then scaffolded by ‘ALLHiC_bulid.’ These resultant scaffolds were further validated and manually curated via Juicebox (v1.11.08) [112] software. Finally, the resultant genome was obtained by ‘juicebox_assembly_converter.py’ given in Juicer package (v1.6) [113]. The custom scripts used in alternative methods and the complete pipeline has been deposited in GitHub (https://github.com/Echoring/ScriptArchive). The quality of the assembly underwent multiple assessments using different approaches. The phasing of haplotypes was confirmed through the KAT program (v2.4.1) [26] with its standard parameters. The BUSCO program (v5.2.1) [28], leveraging the Embryophyta OrthoDB v10 dataset (https://www.orthodb.org), was employed to assess the completeness of the genome assembly. The continuity of the genome assembly was assessed by unitig N50 metrics and the assembly quality was assessed by LAI program in LTR_retriever (v2.9.0) [29]. Also, the alignment of HiFi and Hi-C reads against the assembly was generated to calculate the mapping ratio using BWA (v0.7.17) [108], thus verifying the reliability of the genome assembly. The risk of collapse was detected via mapping depth comparison utilizing mosdepth (v0.3.8) [114]. Meanwhile, the chloroplast genome was assembled utilizing Oatk (v1.0) [63] with HiFi reads.

### Repeat identification and gene annotation

The annotation of transposable elements (TEs) was performed using the EDTA pipeline (v2.0.0) [115], employing its preset configurations. TRF software (v4.09) [116] was utilized to identify tandem repeats (TRs) with the parameters set as 2 7 7 80 10 50 500 -f -d -m. RepeatMasker software (v4.1.2) [117] was used for soft-masking all six haplotype assemblies.

For the annotation of gene structure, BRAKER (v3.0.0) [118] was employed, integrating *ab initio*, transcriptome-based and homologous-protein-based prediction. A total of 12 *A. valvata* RNA-seq datasets were referred to enhance gene prediction accuracy (Table S21). Gene function annotation was conducted using the eggNOG-mapper tool (v2.1.11) against multiple protein sequence databases [30]. GO and KEGG enrichment analysis were facilitated by the R package clusterProfiler (v4.10.0) [119]. Additionally, OrthoFinder (v2.5.4) [37] was used to identify gene families across six alternative haplotypes.

### Telomere and centromere identification

The positions of telomeres and centromeres were determined using the quarTeT (v1.2.0) toolkit [31]. The consensus telomere repetitive sequence AAACCCT, found within a 50 kb range at the end of each chromosome, was designated as a telomere. Regions rich in tandem repeats were considered as indicative of centromere locations. Simultaneously, the dominant monomer inside each centromere region was classified as the centromeric monomer.

### Comparative analysis of genome synteny

Pairwise genome comparisons across the six haplotype assemblies were performed using the MUMmer software suite (v4.0.0beta2) [120], with parameters set as ‘-maxmatch -c 500 -b 200 -l 100’. After alignment, the results were refined using the delta-filter tool with the provided parameters (-m -i 90 -l 100), and the tool show-snps was used to extract SNP and InDel data. The mummerplot tool was subsequently employed to visualize these genomic comparisons using dot plots. Additionally, the Synteny and Rearrangement Identifier (SyRI) (v1.6) [121] was employed to identify collinear orthologs, structural variants, and sequence discrepancies based on the alignments generated by MUMmer.

### Transcriptome library construction and sequencing

RNA-sequencing was performed on samples collected at 0, 1, 2, and 3 days after root flooding (DAF) of two species of kiwifruit (*A. chinensis* cv. ‘Hongyang’ and *A. valvata* cv. ‘DE’). Three biological replicates were obtained for each time point. Total RNA was extracted from the root of these samples, and mRNA libraries were constructed then sequenced using the Illumina NovaSeq 6000 system (Table S22). Raw paired-end RNA-seq reads were first quality controlled and filtered using fastp (v0.23.4) [122] with default parameters. The resulting clean reads were aligned to the reference genome Avhap1 using Hisat2 (v2.1.0) [123]. SAM files were converted to sorted BAM files using samtools (v1.16.1) [109]. GFF3 annotation files were converted to GTF format with gffread (v0.12.7) [124]. Gene counts were generated with featureCounts (v2.0.6) [125] using paired-end mode and the GTF annotation. The resulting counts were combined into a matrix using TrinityRNASeq (v2.8.6)’s [126] script “abundance_estimates_to_matrix.pl.” FPKM and TPM values were calculated in R using edgeR (v4.0.16)’s [127] rpkm function and a standard TPM transformation. Differential expression analysis was performed with DESeq2 [128] via TrinityRNASeq’s script “run_DE_analysis.pl.” Genes with adjusted *P*-value <0.05 and |log2FoldChange| > 1 were considered significantly differentially expressed.

### Identification of allele-specific expressed genes

Alignment blocks covering the entire genome for the six haplotypes were initially retrieved from the results of the synteny analysis. Subsequently, the jcvi.compara.catalog program of the JCVI toolkit (v1.3.6) [129] was then used to identify orthologous gene pairs among different haplotypes with parameters set to ‘--cscore=0.7 --dist=20 --min_size=4’. Syntenic blocks were further filtered to retain only one-to-one allelic gene pairs. All 24 RNA-seq datasets listed in Table S21 were mapped to a haplotype-aware reference genome consisting of all six haplotypes using Hisat2 (v2.1.0) [123] with default settings then assigned to the best-aligned haplotype. Allele-Specific Expressed Genes (ASEGs) were identified based on criteria that |log2FoldChange| values of TPM between two alleles exceed 1 and *P*-value <0.05.

### Phylogenetic and gene-family analysis

Orthologous clusters were identified among 14 selected species using OrthoFinder (v2.5.4) [37]. The species included are *Actinidia arguta* (Aa) [35], *A. chinensis* Hongyang v4.0 (Ac) [27], *A. deliciosa* Acd (Ad) [40], *A. eriantha* MD (Ae) [34], *A. hemsleyana* (Ah) [41], *A. latifolia* KY (Al) [42], *A. polygama* (Ap) [39], *A. rufa* (Ar) [39], *Camellia sinensis* TGY [44], *Solanum lycopersicum* ITAG5.0 [45], *Vitis vinifera* v2.1 [46], *Arabidopsis thaliana* TAIR10 [47], *Oryza sativa* v7.0 [48], and *A. valvata* DE (Av) (this study). Divergence times among these species were estimated using the r8s software (v1.81) [38], incorporating time estimates from TimeTree (http://www.timetree.org) [130]. The expansion and contraction of gene families were analyzed using the CAFÉ5 (v1.1, *K* = 2) tool [131], with the divergence times tree as its input.

Additionally, OrthoFinder (v2.5.4) [37] aided in the discovery of *A. valvata*-specific genes by comparing the full protein sequences of eight Actinidia species (Aa, Ac, Ad, Ae, Ah, Al, Ap, Ar). The specific genes were further validated by mapping them against other haplotypes to check whether a homologous gene exists. In addition, alignment blocks covering the entire genome were extracted from the six haplotypes after synteny analysis, and JCVI (v1.3.6) [61] was used to identify allelic gene pairs with the highest coding protein sequence similarity. *K_S_* values were calculated using ParaAT (v2.0) [132] and KaKs_Calculator (v2.0) [133]. The peak *K_S_* values were then converted to divergence times using the formula *T* = *K_S_*/(2*r*), where T represents divergence time and r denotes the neutral substitution rate (*r* = 3.39 × 10^−9^) [134].

Chloroplast genomes of *Actinidia* spp. were downloaded from NCBI database, including *A. arguta* (NC_034913.1), *A. chinensis* (NC_026690.1), *A. deliciosa* (NC_026691.1), *A. eriantha* (NC_034914.1), *A. kolomikta* (NC_034915.1), *A. macrosperma* (MN520000.1) and *A. polygama* (NC_031186.1), with *A. thaliana* (NC_000932.1) as outgroup. These genomes are aligned by HomBlocks (v1.0) [135], then the phylogenetic tree was constructed utilizing RAxML (v8.2.12) [136] with 1000 bootstraps.

### Analysis of ERF gene family and gene co-expression network construction

We performed domain annotation for all protein sequences of *A. valvata* using the PfamScan tool (https://www.ebi.ac.uk/Tools/pfa/pfamscan/). Proteins containing the AP2 domain (PF00847) and ERF superfamily (PF04404) were identified as potential AP2/ERF superfamily candidates, and proteins containing a single AP2 domain were identified as ERF gene family candidates. Additionally, we employed the Search tool in InterPro (https://www.ebi.ac.uk/interpro/search/sequence/) to re-validate the domains of the candidate genes.

The DEGs of different waterlogging periods (DAF0, DAF1, DAF2, DAF3) were used to construct a weighted gene co-expression network of *A. chinensis* and *A. valvata* using WGCNA package (v1.73) [137]. Co-expression modules were identified using the automatic Network Building function (blockwiseModules) with a minimum module size of 30 and a module shear height of 0.25. Module signature genes are used to describe the most common gene expression models in each module. The KME value was calculated based on the Pearson correlation coefficient between the expression level and the module characteristic genes. We then selected shared genes in the two *Actinidia* species with gene significance (GS) ≥ 0.8 to analyze hubgenes in the gene co-expression network. For module-trait associations, *P*-values were adjusted using the FDR method (threshold: 0.05) to control false discovery rates. The network is built using Cytoscape software (v3.10.2) [138]. The *cis*-acting elements in the promoter region were searched by PlantPan (http://plantpan.itps.ncku.edu.tw/index.html) [97].

### Root activity, RNA extraction, and qRT-PCR

The plant roots activity was measured via TTC (2,3,5-Triphenyltetrazolium chloride) staining following the manufacturer’s instructions (Solarbio, BC5270). Total RNA was isolated using the FastPure Universal Plant Total RNA Isolation Kit (Vazyme). First-strand cDNA was synthesized with the HiScript II Q RT SuperMix (Vazyme). qPCR analysis was performed using the ChamQ Universal SYBR qPCR Master Mix (Vazyme). Relative transcript levels were quantified following the method described by Zheng *et al*. [139] Primer sequences are provided in Table S22. Kiwifruit Actin [22] was used as the endogenous reference.

### Plasmid construction and plant transformation

The coding sequences (CDS) of *AcNAC010* and *AvNAC010* were cloned into the pJG4-5 vector respectively. The 2000-bp fragments upstream of the start codons for *AcADH1* or *AvADH1* were cloned into the pLacZi vector. The CDS of *AcNAC010* or *AvNAC010* were cloned into the pCAMBIA2300-35S-eGFP vector to generate the *35S*::*AcNAC010-eGFP* or *35S*::*AvNAC010-eGFP* vectors. The full-length sequence of the *AcADH1* and *AvADH1* promoter (−2000 to −1) was cloned into the pGreenII 0800-LUC vector to generate the *ProAcADH1*::*LUC* and *ProAvADH1*::*LUC* vectors. All primers used for cloning are listed in Table S22.

### Yeast one-hybrid assay

The yeast one-hybrid assay was performed according to the method of Zhang *et al.* [140]. Different plasmid combinations were co-transformed into the yeast strain EGY48 and cultured on SD/-Trp/-Ura medium for 3 days. Selection was performed on SD/-Trp/-Ura/Gal/Raf/X-Gal medium.

### Dual-luciferase reporter assay

The dual-luciferase reporter assay was conducted according to the method of Di *et al.* [141]. The *35S*::*AvNAC010*-*eGFP* vector and *ProAvADH1*::*LUC* vector as well as *35S*::*AcNAC010*-*eGFP* vector and *ProAcADH1*::*LUC* vector was transferred into *Agrobacterium* GV3101 and transiently expressed in tobacco leaves for 36–48 h, respectively. Images were captured using a cold CCD imaging system (4600SF, Tanon), and luciferase activity was detected using a dual-luciferase reporter assay kit (DL101-01, Vazyme).

### Statistical analysis

Significant differences were calculated using Student’s *t*-test, where ^*^ denotes *P* < 0.05 and ^**^ denotes *P* < 0.01. Statistical analysis details and sample sizes for each experiment are indicated in the figure legends.

## Abbreviations

*A. arguta: Actinidia arguta; A. chinensis: Actinidia chinensis; A. deliciosa: Actinidia deliciosa; A. eriantha: Actinidia eriantha; A. hemsleyana: Actinidia hemsleyana; A. latifolia: Actinidia latifolia; A. polygama: Actinidia polygama; A. rufa: Actinidia rufa; A. valvata: Actinidia valvata;* ADH: alcohol dehydrogenase; ASE: allele-specific expression; CDS: coding sequences; CTAB: cetyltrimethylammonium bromide; DEG: Differentially expressed gene; GO: Gene ontology; InDel: insertions or deletions; KEGG: Kyoto Encyclopedia of Genes and Genome; *K_S_*: Synonymous substitution rate; LAI: LTR assembly index; LTR: Long Terminal Repeat; LUC: Luciferase; PDC: pyruvate decarboxylase; SDR: sex-determining region; SNP: single-nucleotide polymorphisms; TEs: transposable elements; TPM: transcripts per kilobase per million mapped reads; TPS: trehalose-6- phosphate synthase; TRs: tandem repeats; WGCNA: weighted gene co-expression network analysis; WGD: whole-genome duplication; Y1H: yeast one-hybrid.

## Consent for publication

All authors hereby consent to publication of the work.

## Supplementary Material

Web_Material_uhag011

## Data Availability

All data generated or analyzed during this study are included in this published article. The raw Hi-Fi, Hi-C and RNA-seq reads, assembled genome, and annotations generated in this study have been deposited in the NGDC database (https://ngdc.cncb.ac.cn/) with the accession number PRJCA030394.
