# Supplementary material for: Haplotype-resolved genome assembly provides insights into the evolutionary origin of waterlogging-tolerant Actinidia valvata hexaploid
Source: Hortic Res. 2026 Jan 9;13(4):uhag011. doi: 10.1093/hr/uhag011 (PMC13103481; doi:10.1093/hr/uhag011)
Supplement: Web_Material_uhag011 [file web_material_uhag011.zip › 02.supplementaryFigureRevised_R2.docx]

**Supplementary Figures**


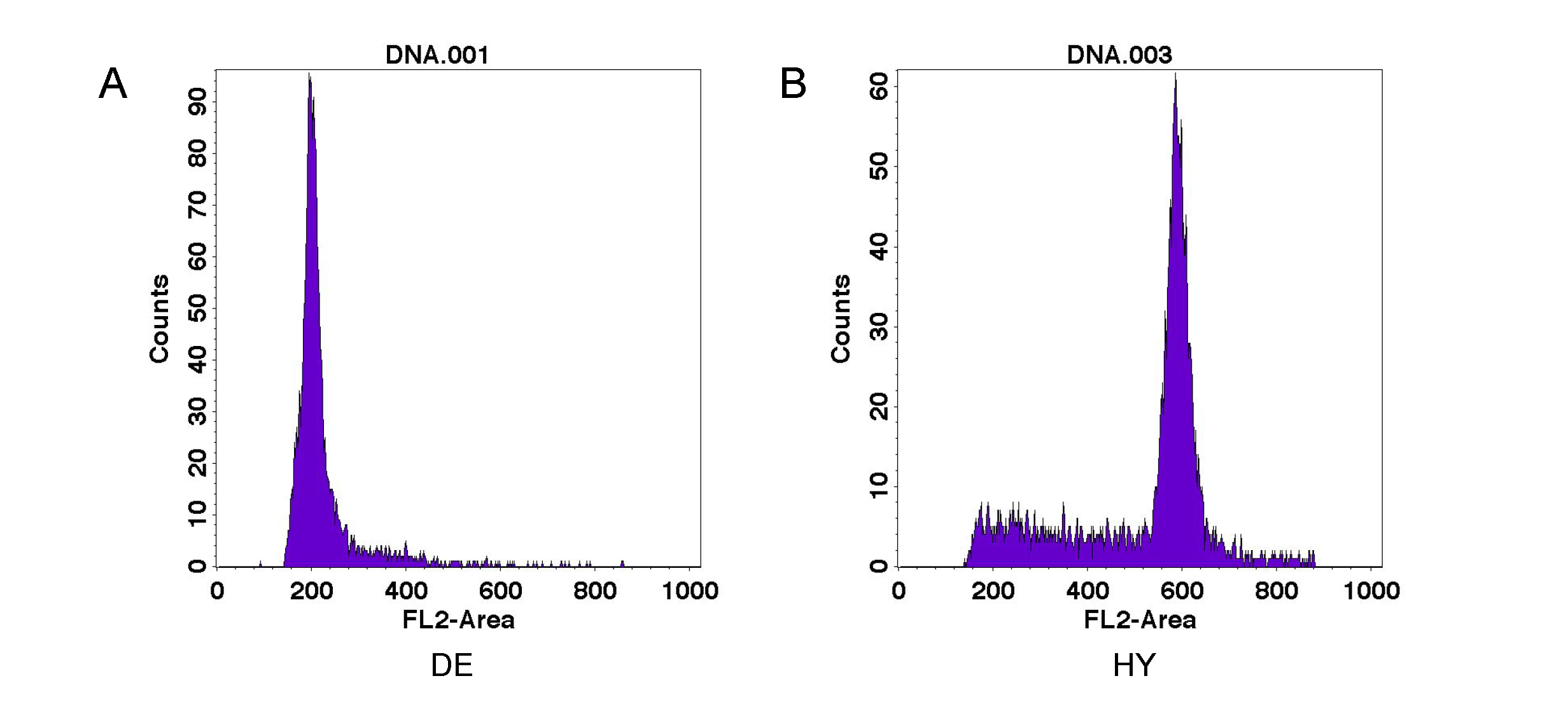


**Figure S1**. Identification of kiwifruit ploidy by flow cytometry. *Actinidia chinensis* cv. ‘Hongyang’ (A) is diploid and *Actinidia valvata* cv. ‘DE’ (B) is hexaploid.


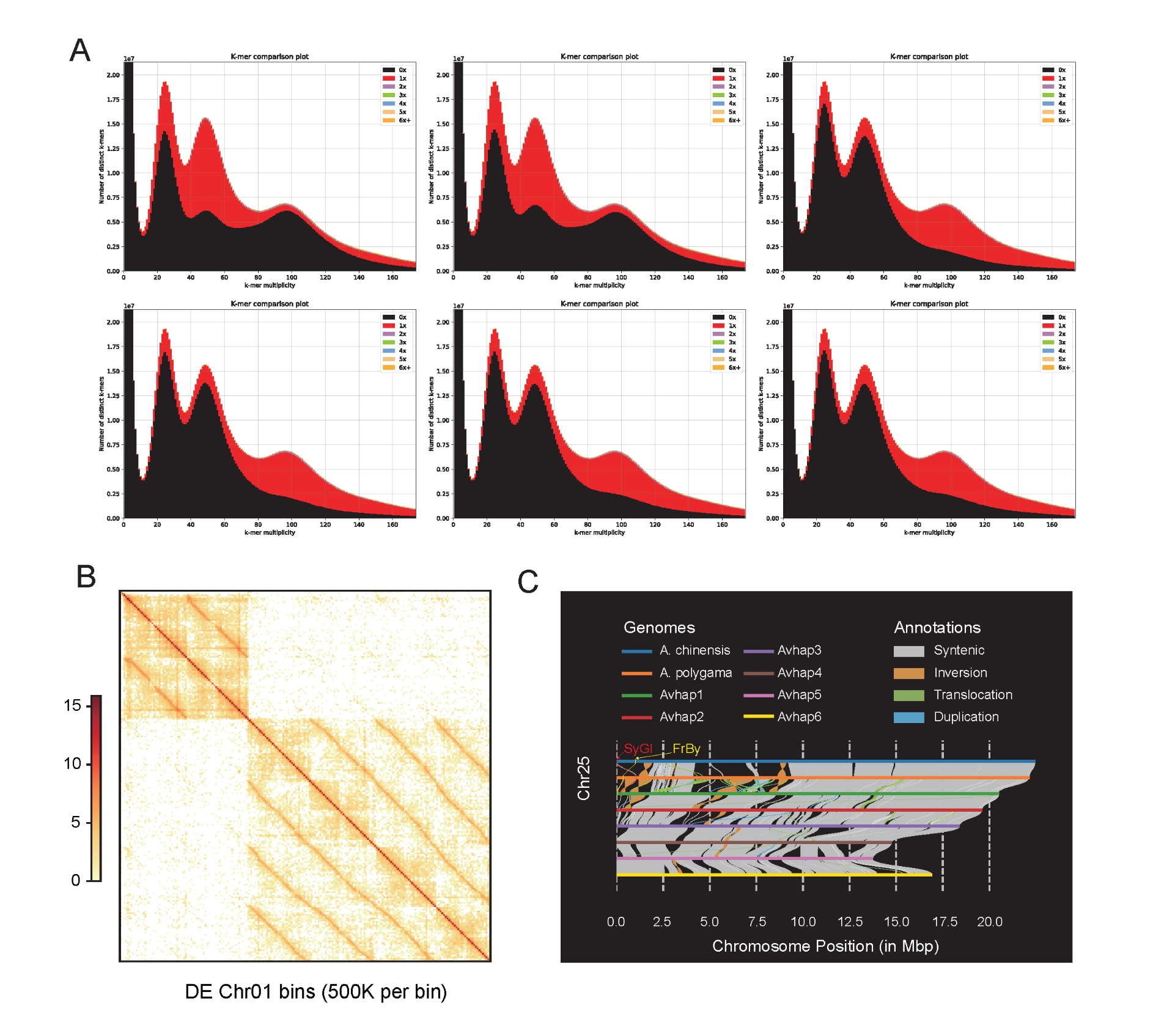


**Figure S2.** Ploidy and phasing validation of *Actinidia valvata* cv. ‘DE’. (A) Comparison of the distinct K-mers frequency between the six individual haplotypes of the *A. valvata* assembly and the raw HiFi reads, respectively. (B) The Hi-C contact heatmap illustrates the signals among six haplotypes. (C) Collinearity between male *A. chinensis* and *A. valvata* on the sex chromosome (Chr25) The red and yellow lines mark the location of the *SyGl* and *FrBy* genes.


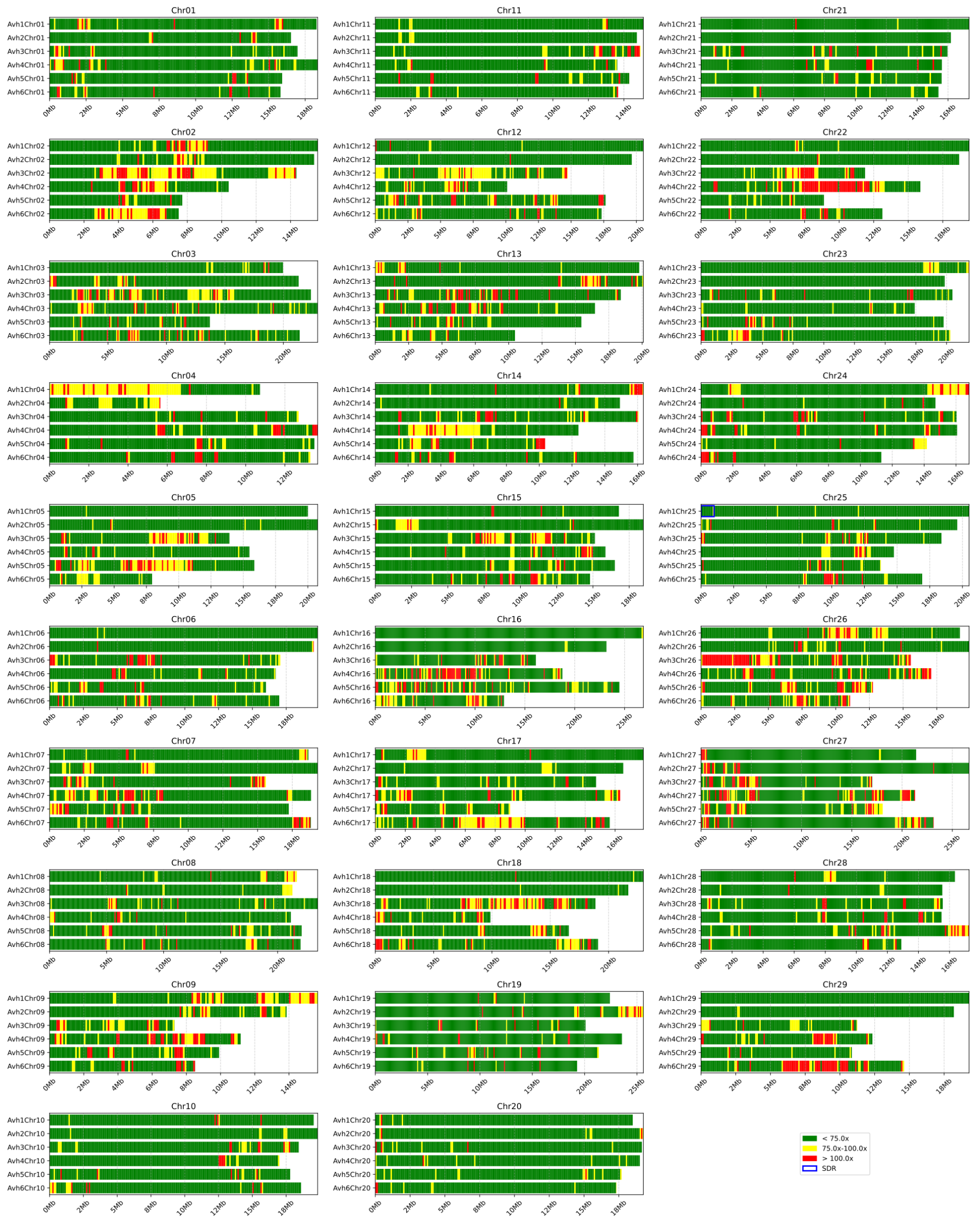


**Figure S3.** Mapping depth distribution of HiFi raw reads against whole genome. The sequencing depth of genome is 48.3-fold. Colored blocks from green to red indicate the risk of collapse from low to high. Sex-determining region (SDR) are highlighted in blue.


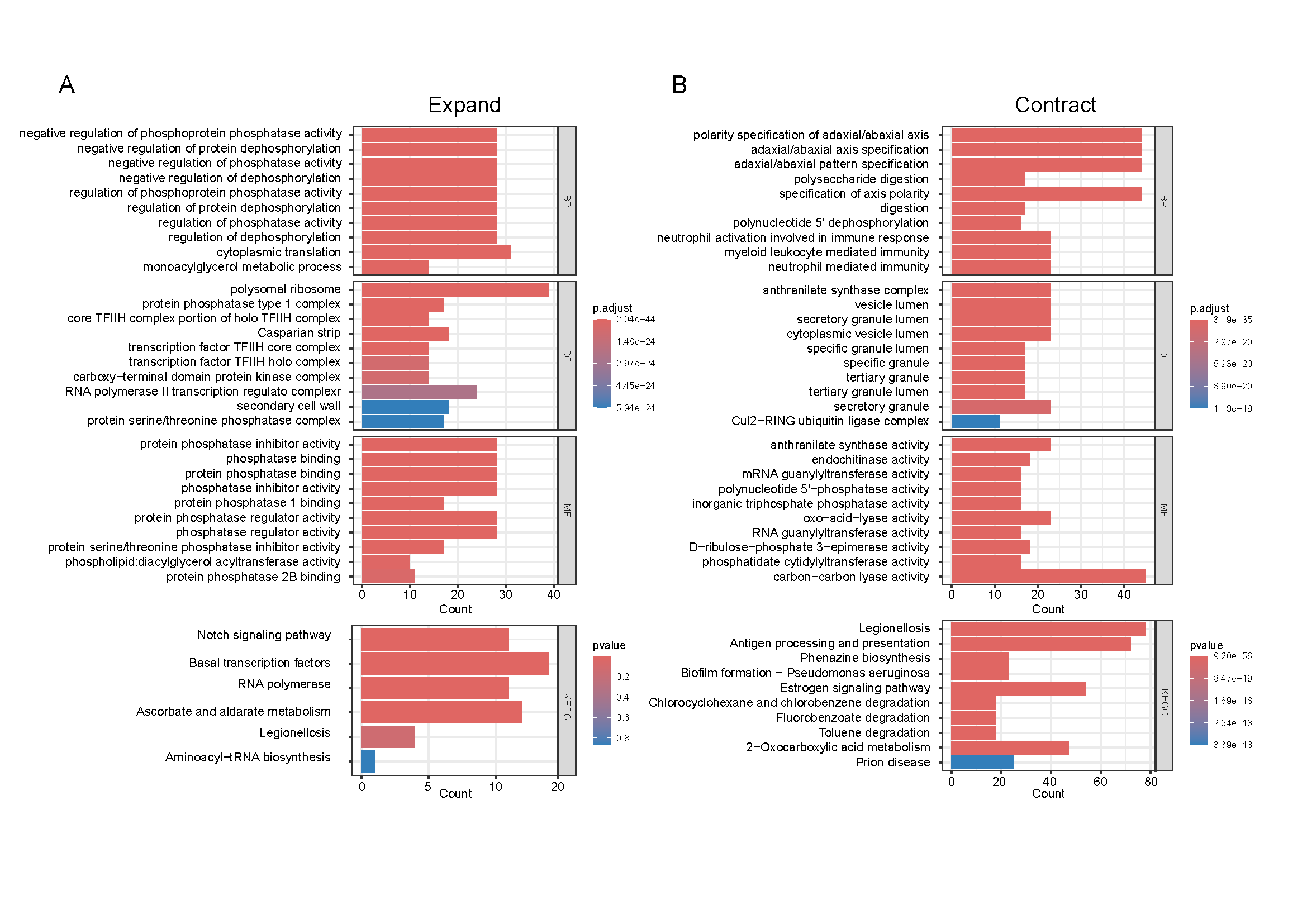


**Figure S4.** Gene Ontology (GO) and Kyoto Encyclopedia of Genes and Genomes (KEGG) enrichment of expanded (A) and contracted (B) gene families in *Actinidia valvata.*


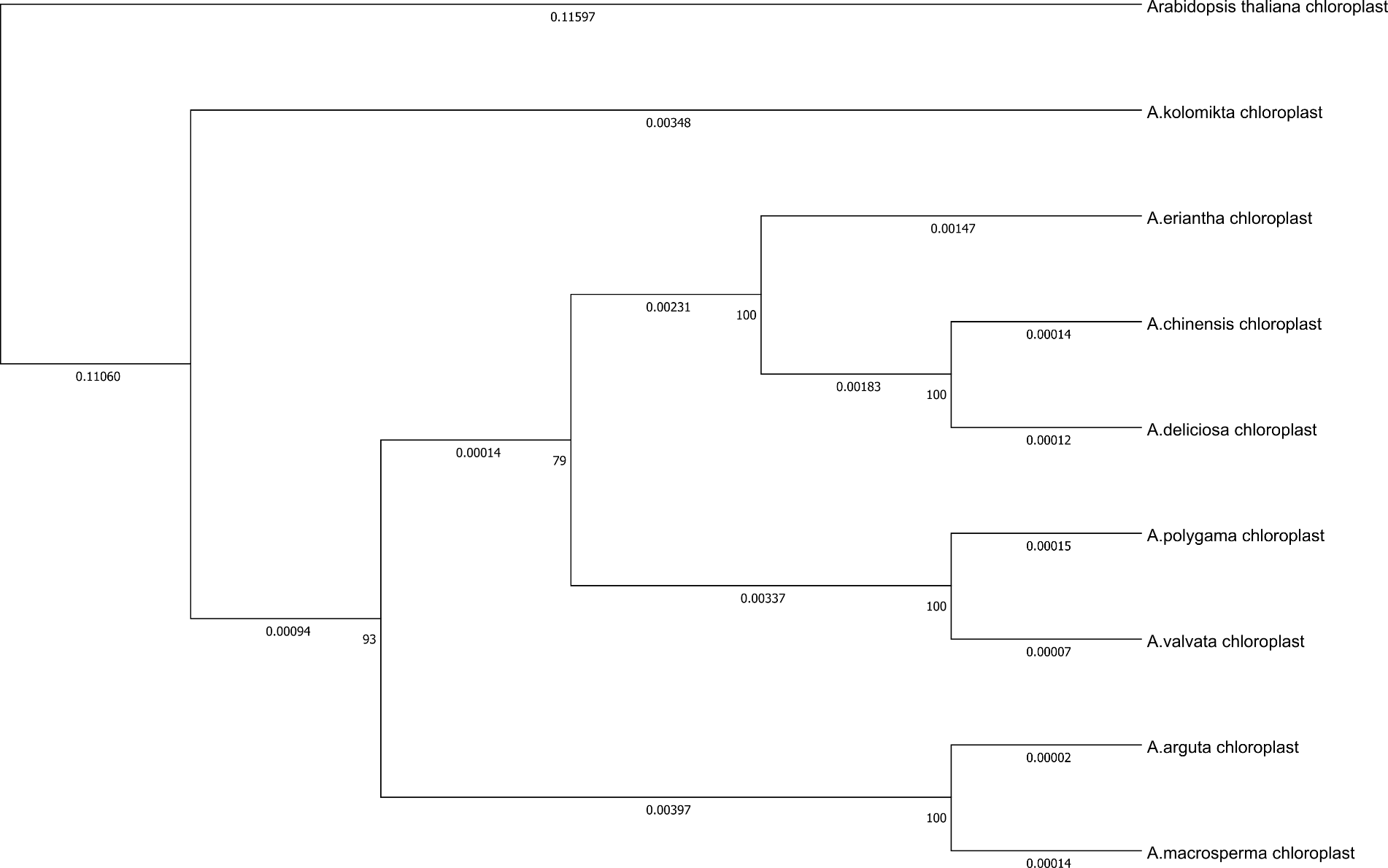


**Figure S5.** Phylogenetic tree of chloroplast genome of *Actinidia* species. Numbers on node indicate bootstraps support value, while numbers on branch demonstrate evolutionary distance.


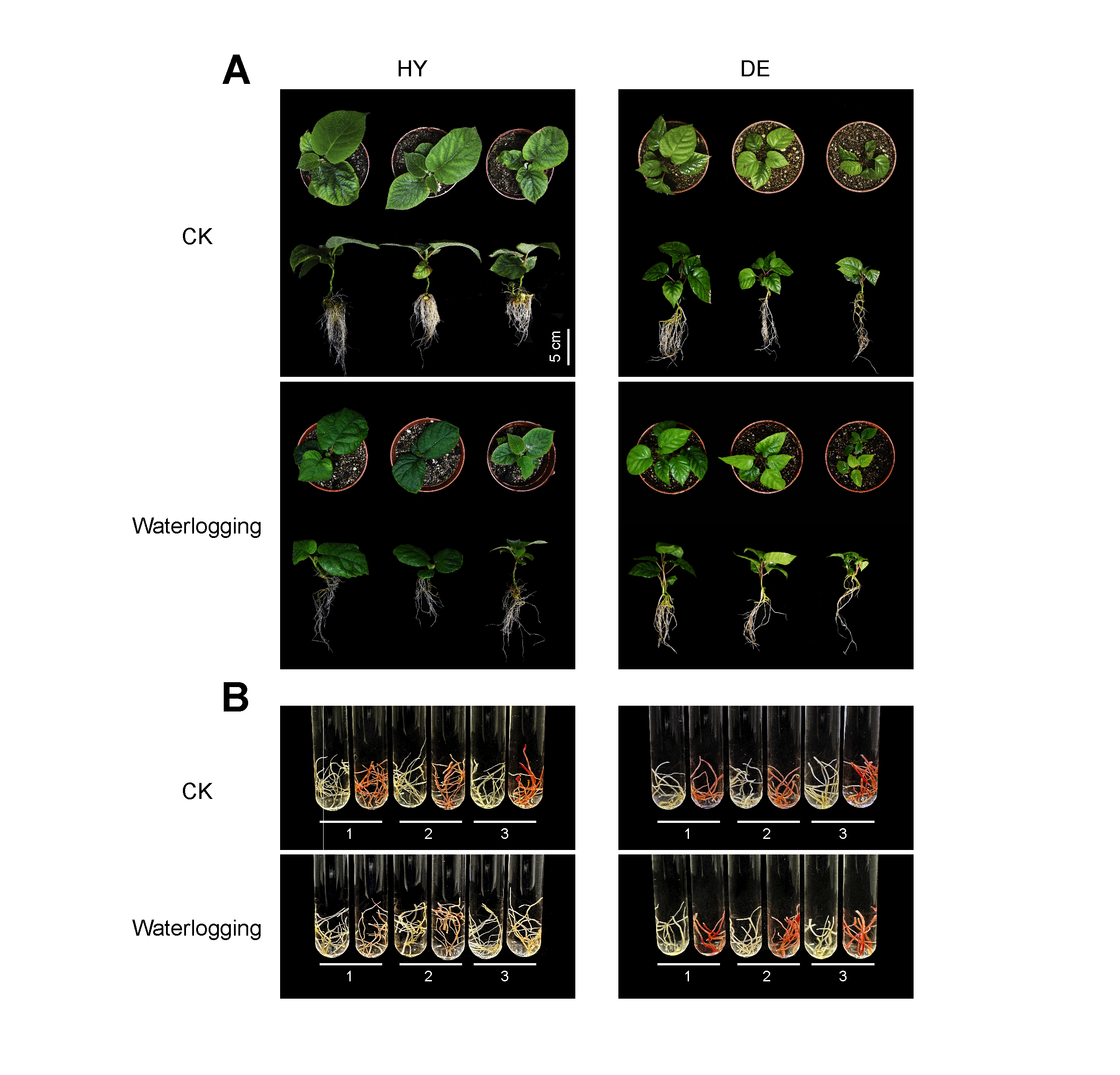


**Figure S6.** Comparison of waterlogging responses and root activity between *Actinidia chinensis* cv. ‘Hongyang’ (‘HY’) and *A. valvata* cv. ‘DE’ after 3 days of flooding stress. (A) Photographs showing the leaf and root growth status of ‘HY’ and ‘DE’ under normal (CK) and waterlogged conditions. (B) Root images of TTC staining of ‘HY’ and ‘DE’. In each biological replicate (labeled with Arabic numerals), the left tube represents the blank control, and the right tube contains samples subjected to a 4 h incubation reaction.

**
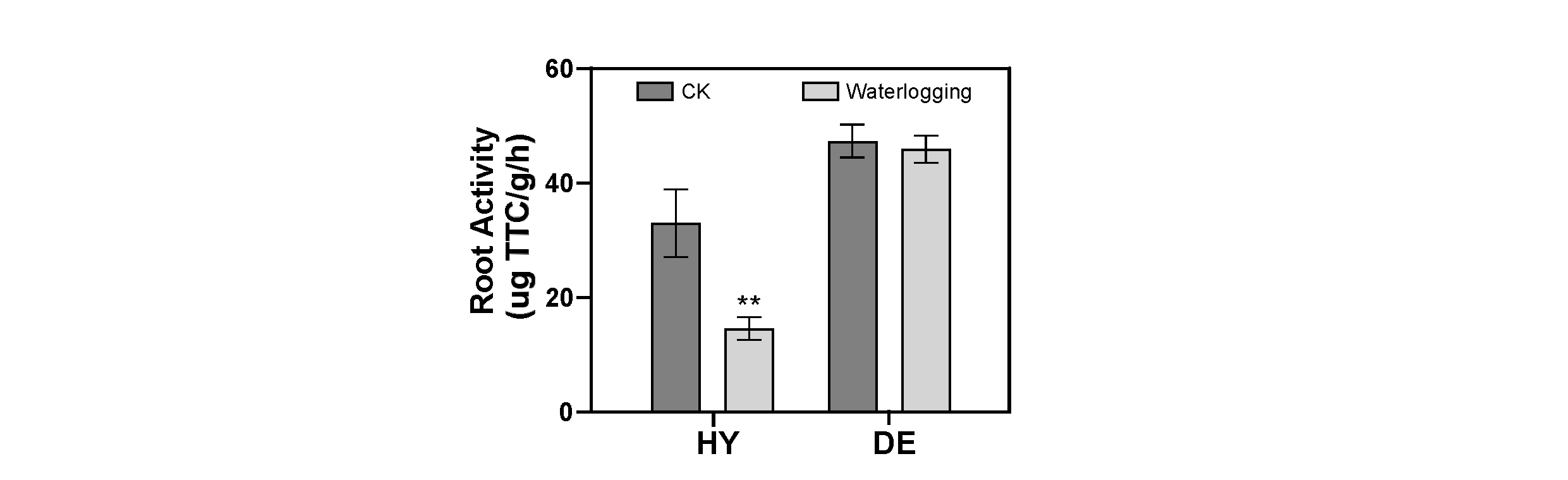
**

**Figure S7.** Comparison of the root activities between *Actinidia chinensis* cv. ‘Hongyang’ (HY) and *A. valvata* cv. ‘DE’. CK represents control, while WL represents waterlogging treatment. 0d and 3d represent the days after treatment. Statistical significance was determined using Student’s t-test, with ** indicating P < 0.01.


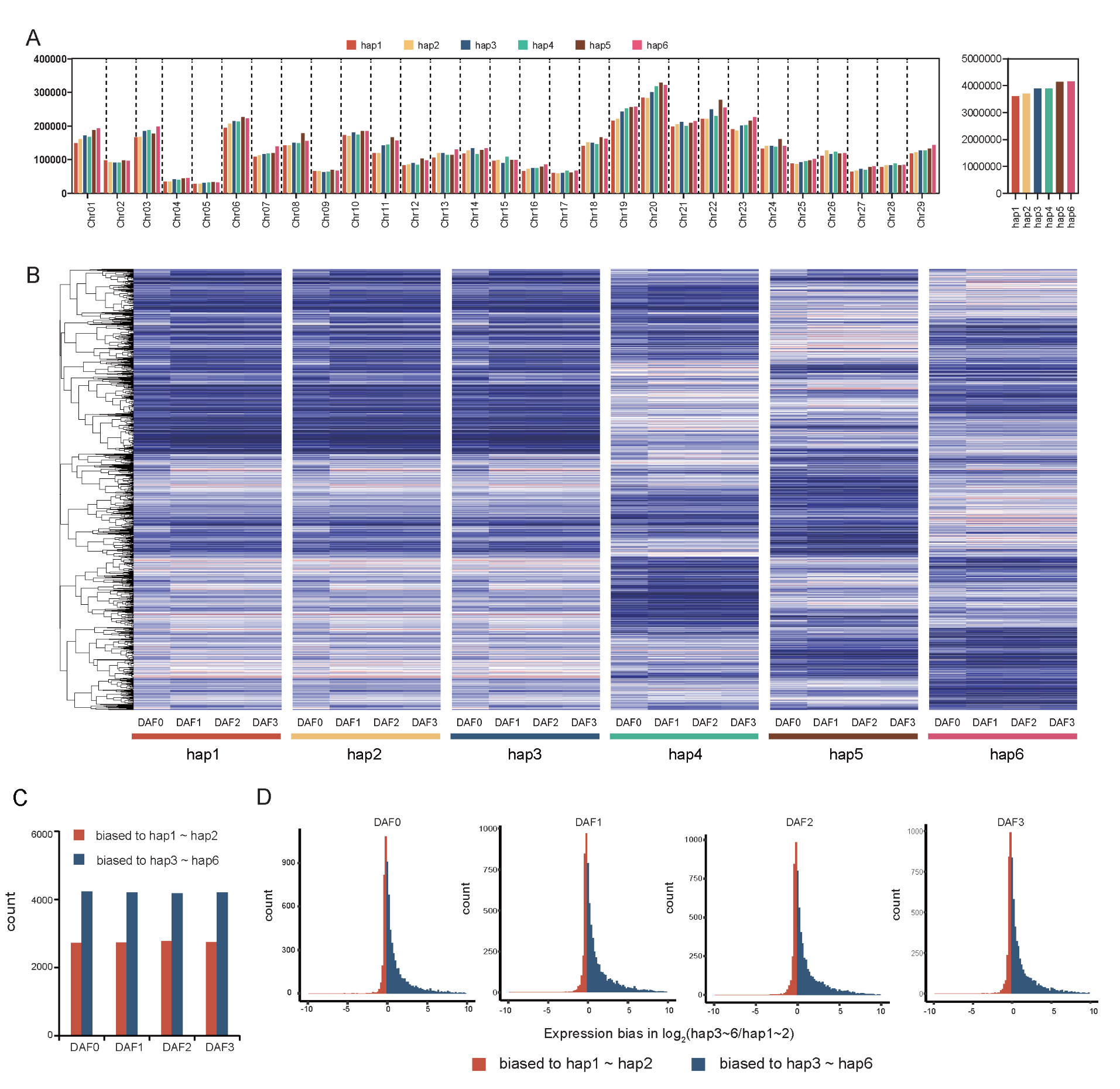


**Figure S8.** Genomic expression patterns of *Actinidia valvata.* (A) The accumulation of gene expression levels for the genes exhibiting six alleles. Expression levels are quantified in transcripts per kilobase per million mapped reads (TPM). (B) Expression patterns of allele-specific expressed genes (ASEGs) among the six haplotypes at various flooding times (0, 1, 2, and 3 days after root flooding, DAF). (C) The count of unbalanced sextuple-allelic gene groups. (D) Frequency distribution of gene expression Log2FoldChange values.


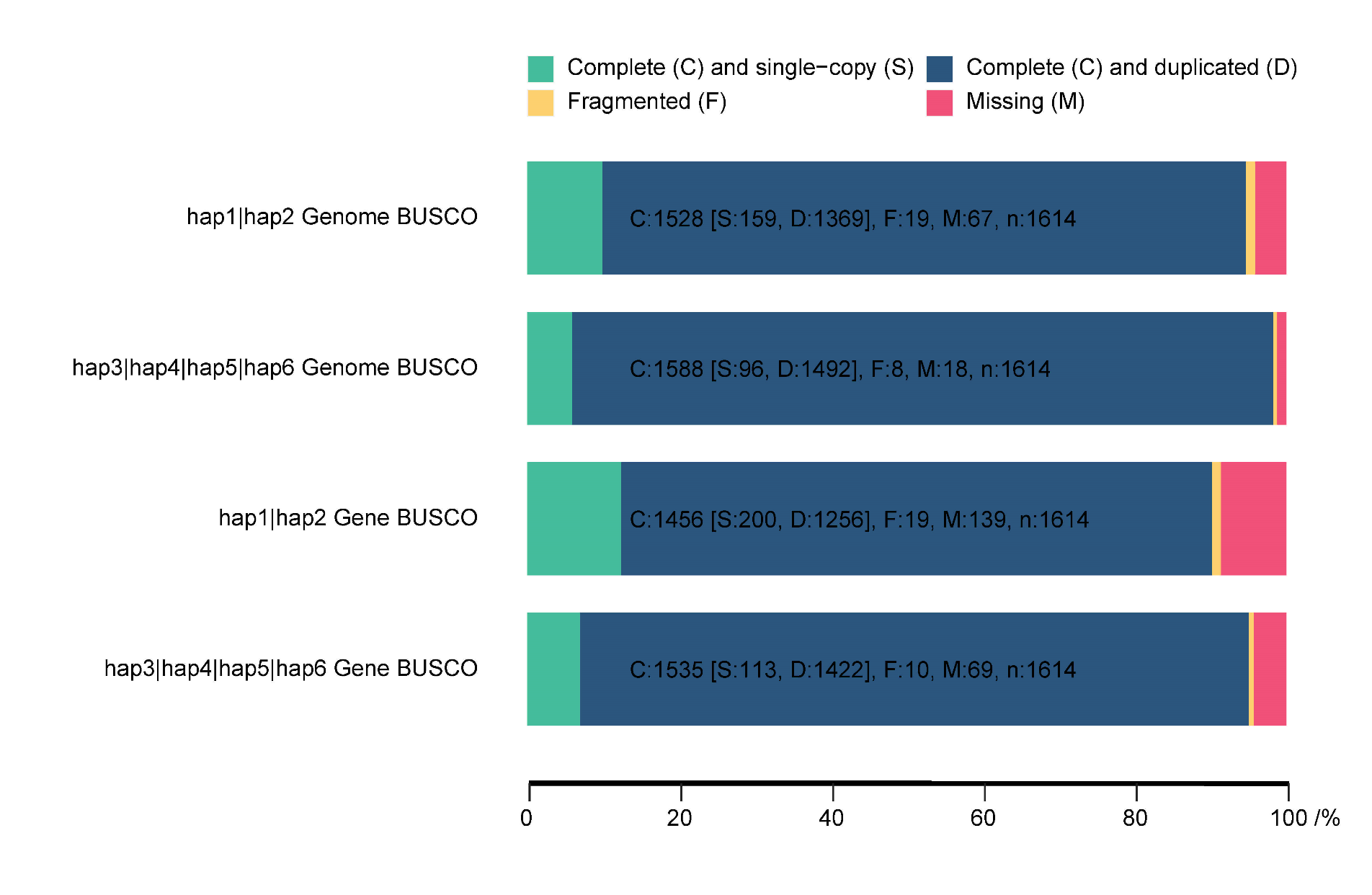


**Figure S9.** Subgenome-specific genome BUSCO and gene BUSCO assessments showing proportions classified into categories of complete and single-copy (S, green), complete and duplicated (D, blue), fragmented (F, yellow), and missing (M, pink).

**
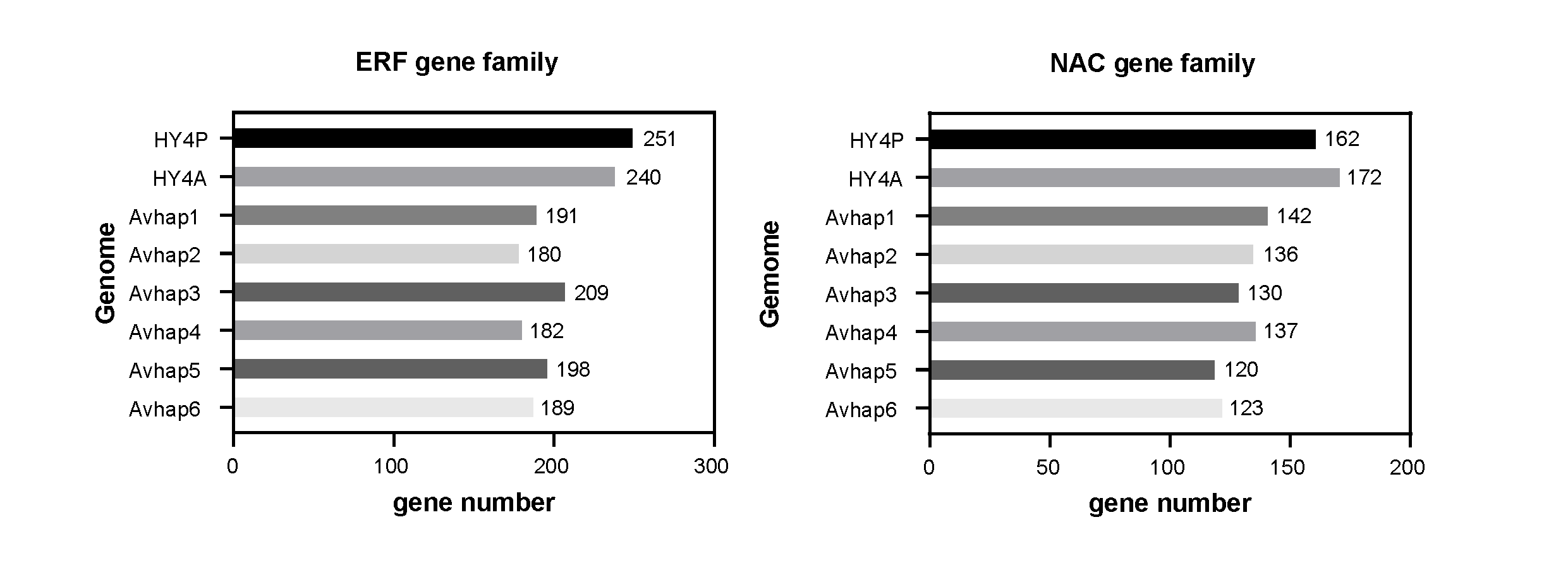
**

**Figure S10.** Genome-wide identification of ERF and NAC gene family in *Actinidia chinensis* and *A. valvata*.


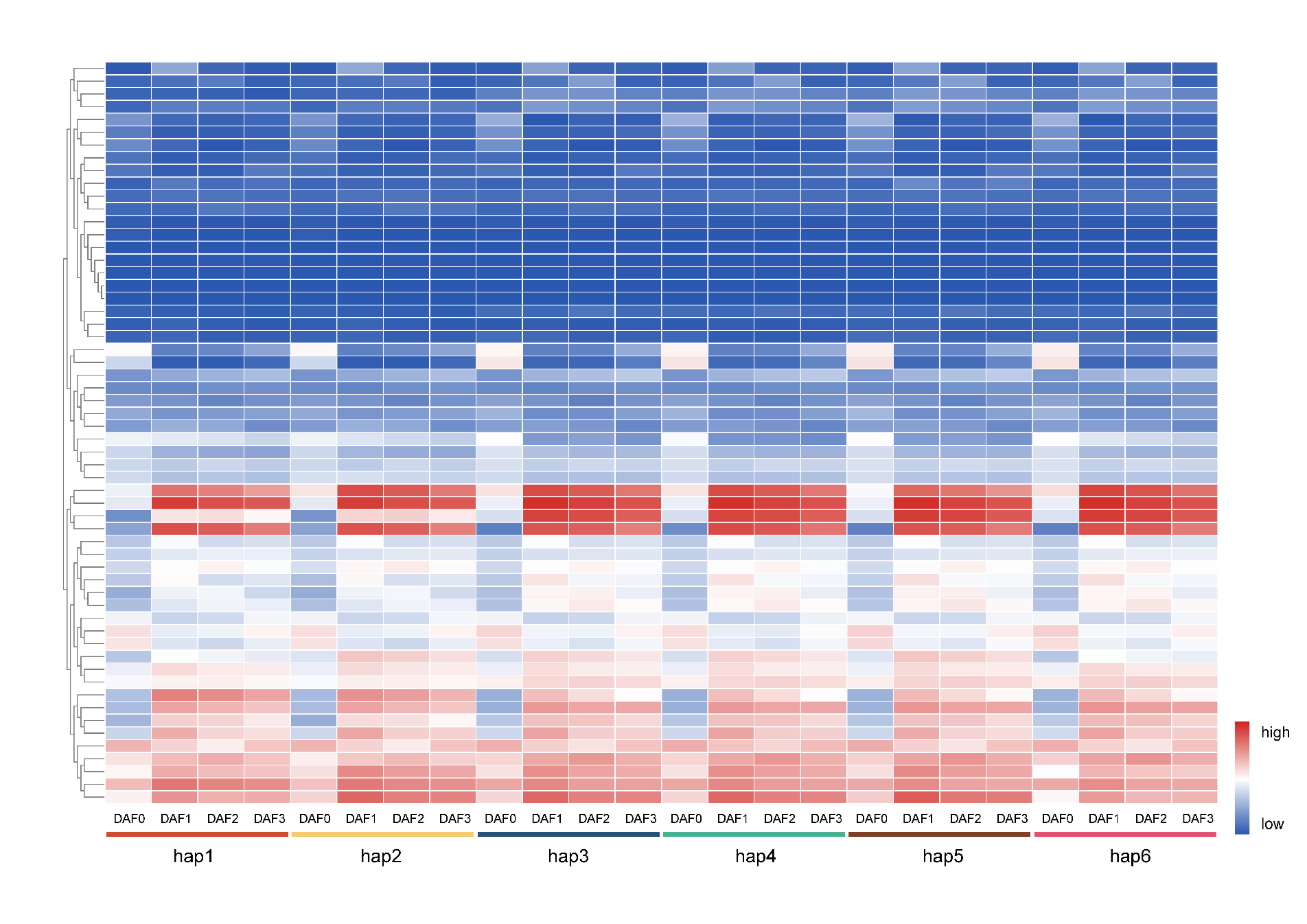


**Figure S11.** The expression patterns of the ERF family genes which showed allele-specific expression (ASE) under varying durations of waterlogging treatment in Actinidia valvata.


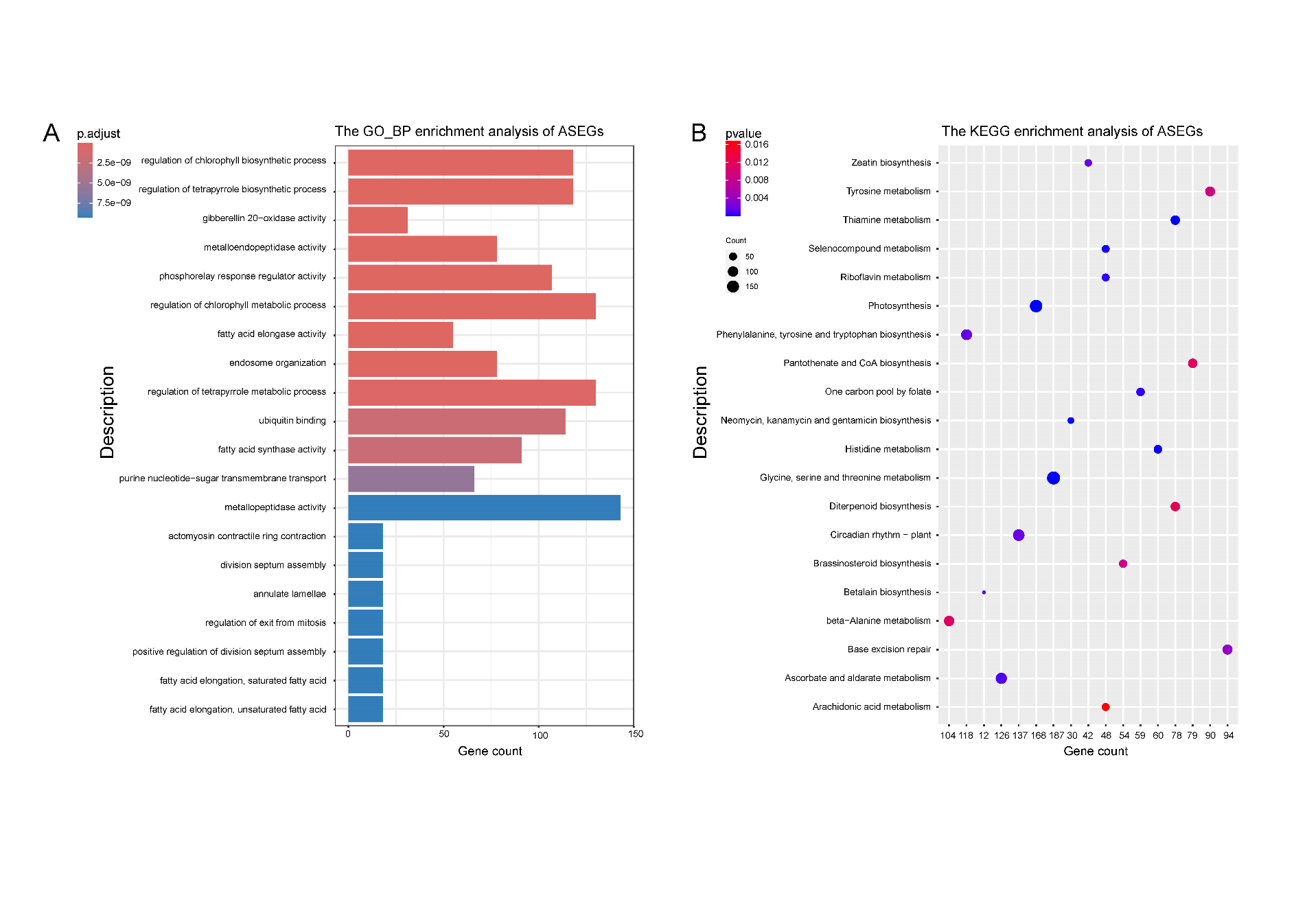


**Figure S12.** Enrichment analysis of GO and KEGG pathways for allele-specific expressed genes (ASEGs) in *Actinidia valvata*. (A) Enriched GO terms associated with ASEGs in the biosynthetic process. (B) Enriched KEGG pathways associated with ASEGs.


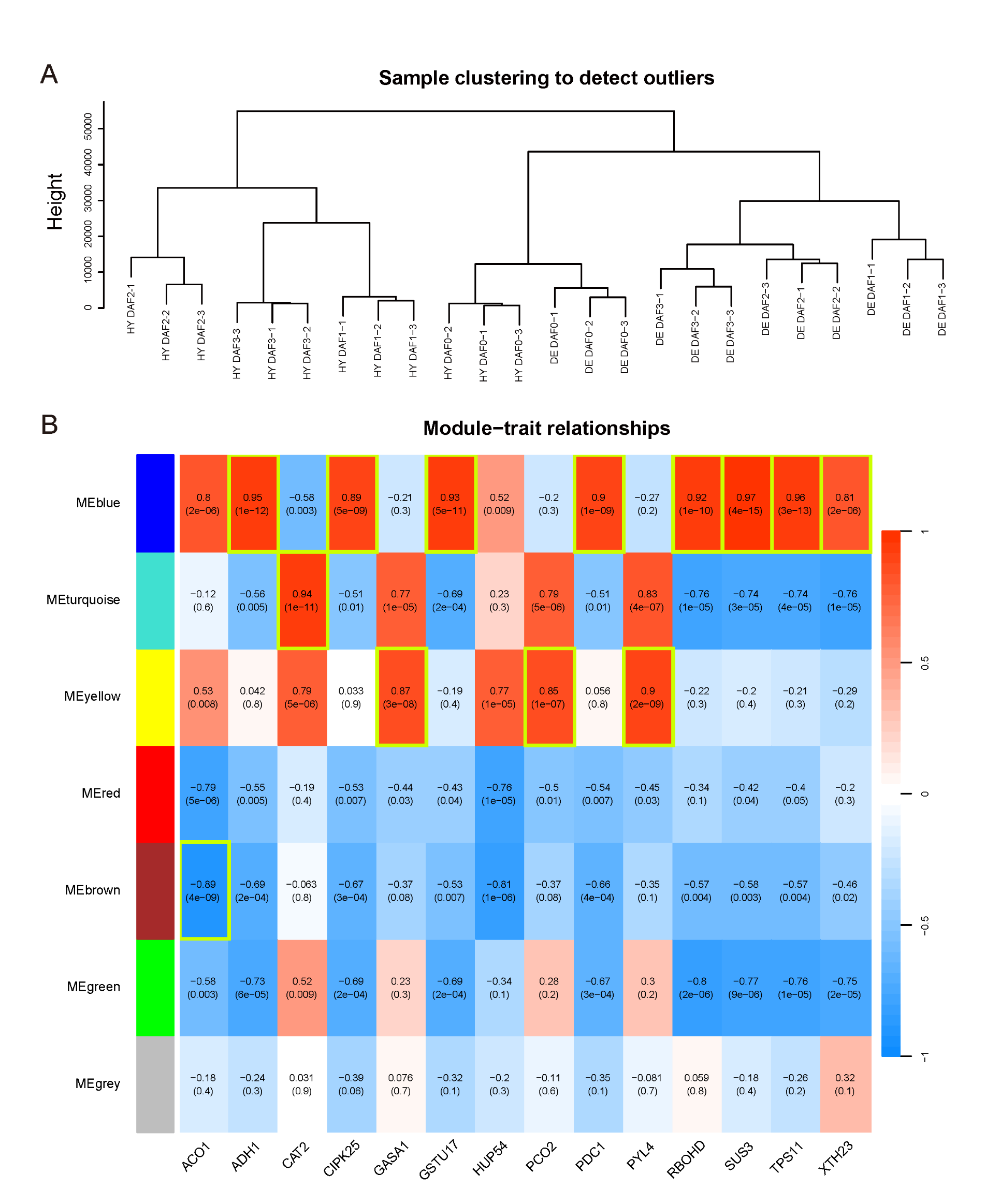


**Figure S13.** Weighted gene co-expression network analysis (WGCNA) using the expression patterns of waterlogging-related genes under waterlogging stress. (A) Sample clustering dendrograms of *Actinidia chinensis* and *A. valvata* samples for RNA-seq based on co-expression network analysis. (B) The heatmap indicated the correlation between the unigenes clustered in each module and the expression level of waterlogging related differentially expressed genes (DEGs). The numbers in each cell represent the module-sample correlation coefficient and the corresponding P-value.


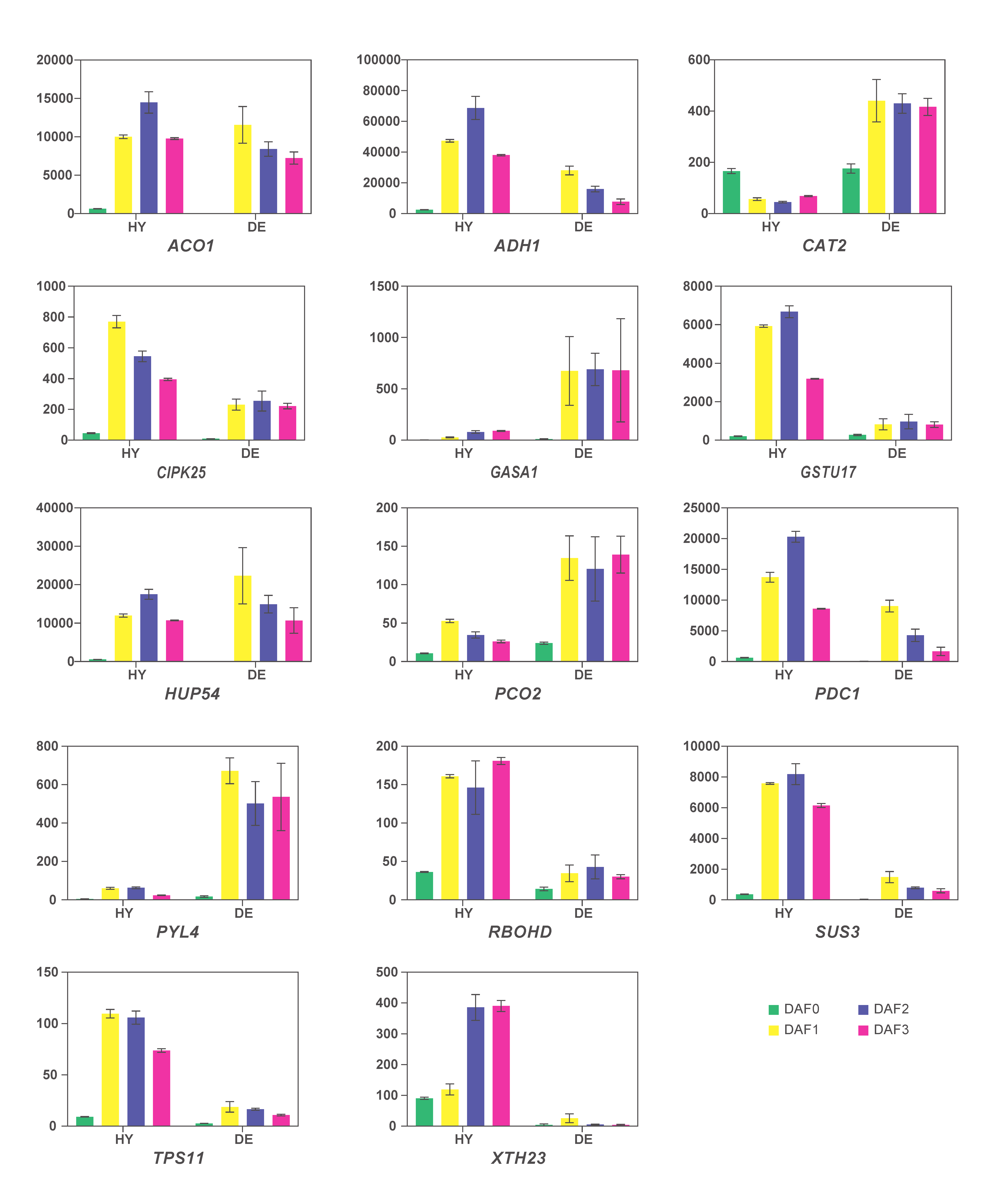


**Figure S14.** Bar plots of expression levels of focused waterlogging related genes in *Actinidia chinensis* ‘HY’ and *A. valvata* ‘DE’ at 0, 1, 2 or 3 days after root flooding (DAF). Expression levels are quantified in.TPM.


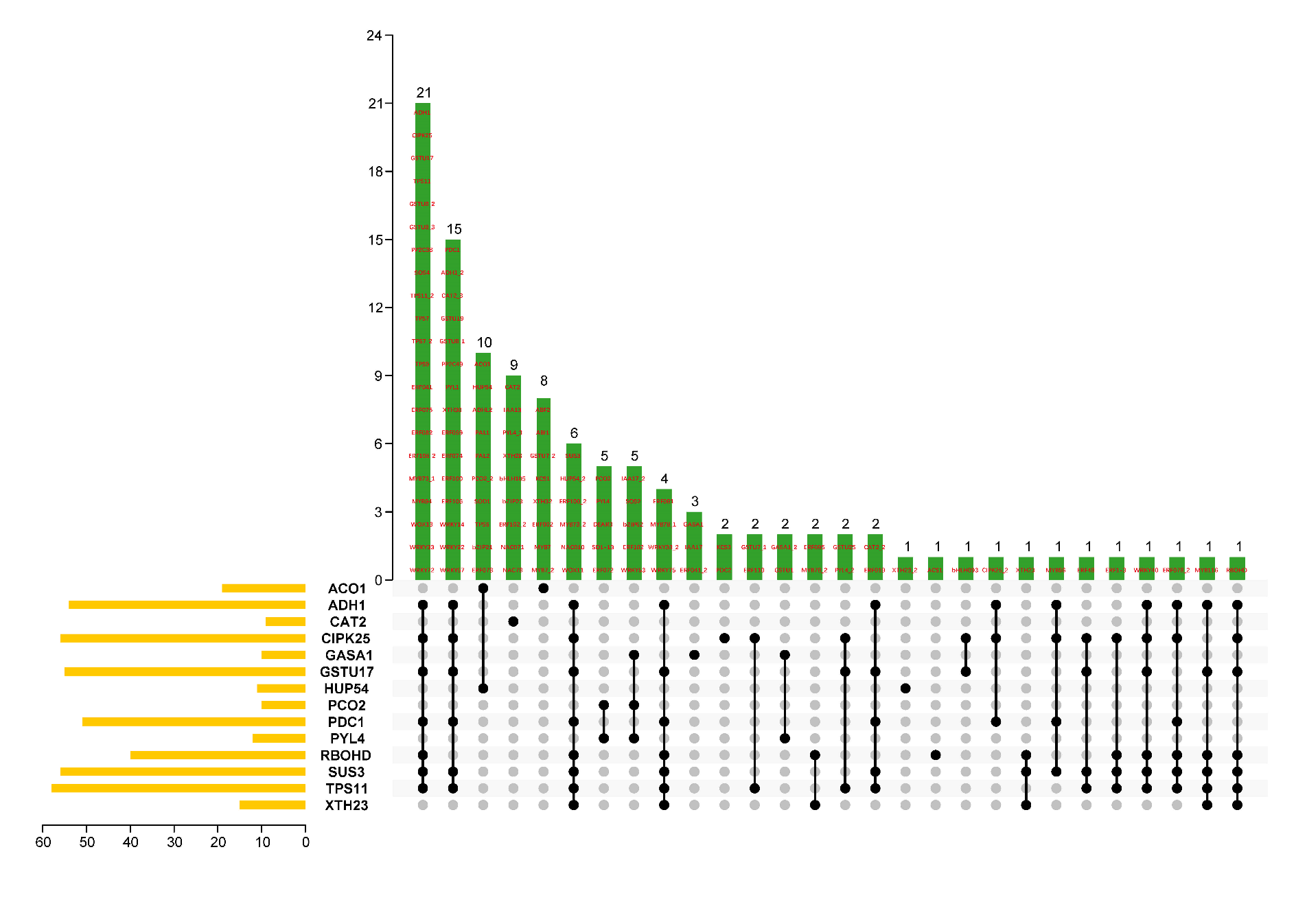


**Figure S15.** The upset diagram showing intersection of transcription factors and gene set in co-expression group that correlation coefficient exceeds 0.85 related to waterlogging resistance. The green or yellow histogram represents the number of genes and transcription factors in each intersection or co-expression group.


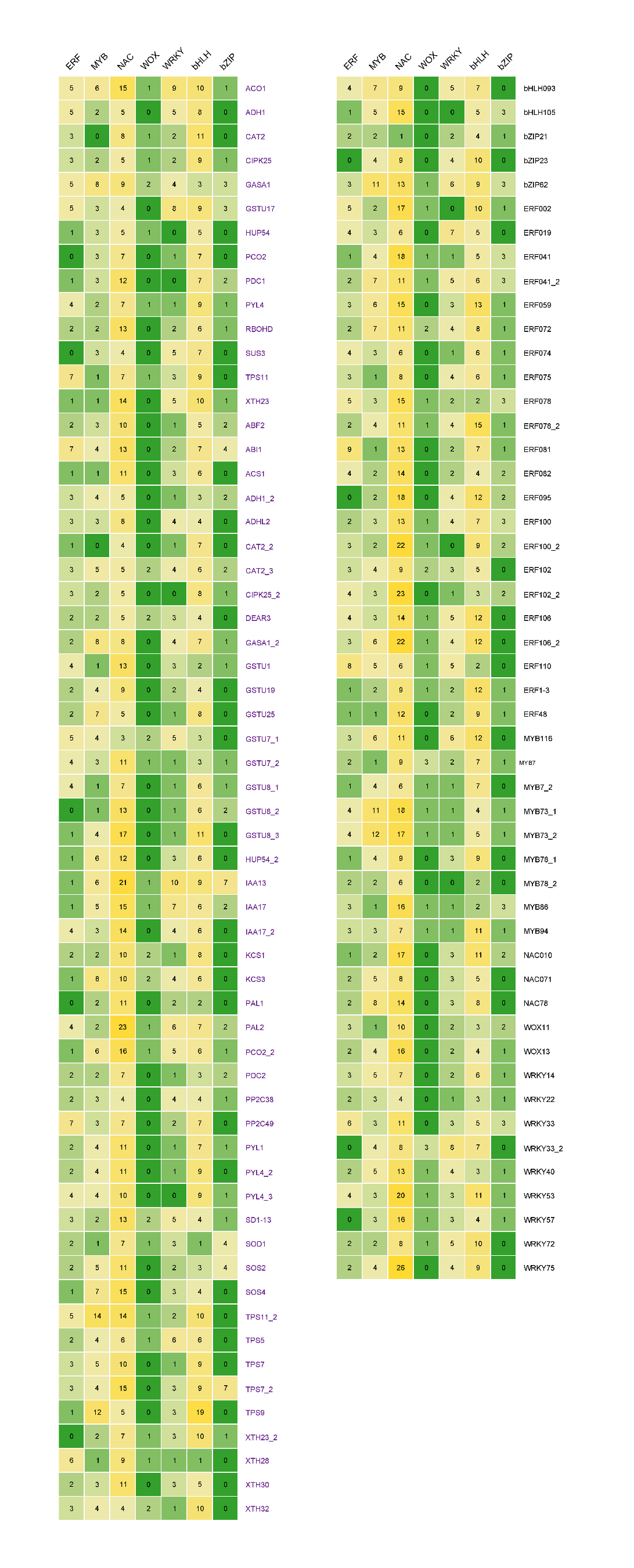


**Figure S16.** Analysis of transcription factor binding sites in the promoter regions of waterlogging-related transcription factors and structural genes. The red text represents structural genes, while the black text represents transcription factors; the number of binding sites is indicated by both color and numerical values.

**
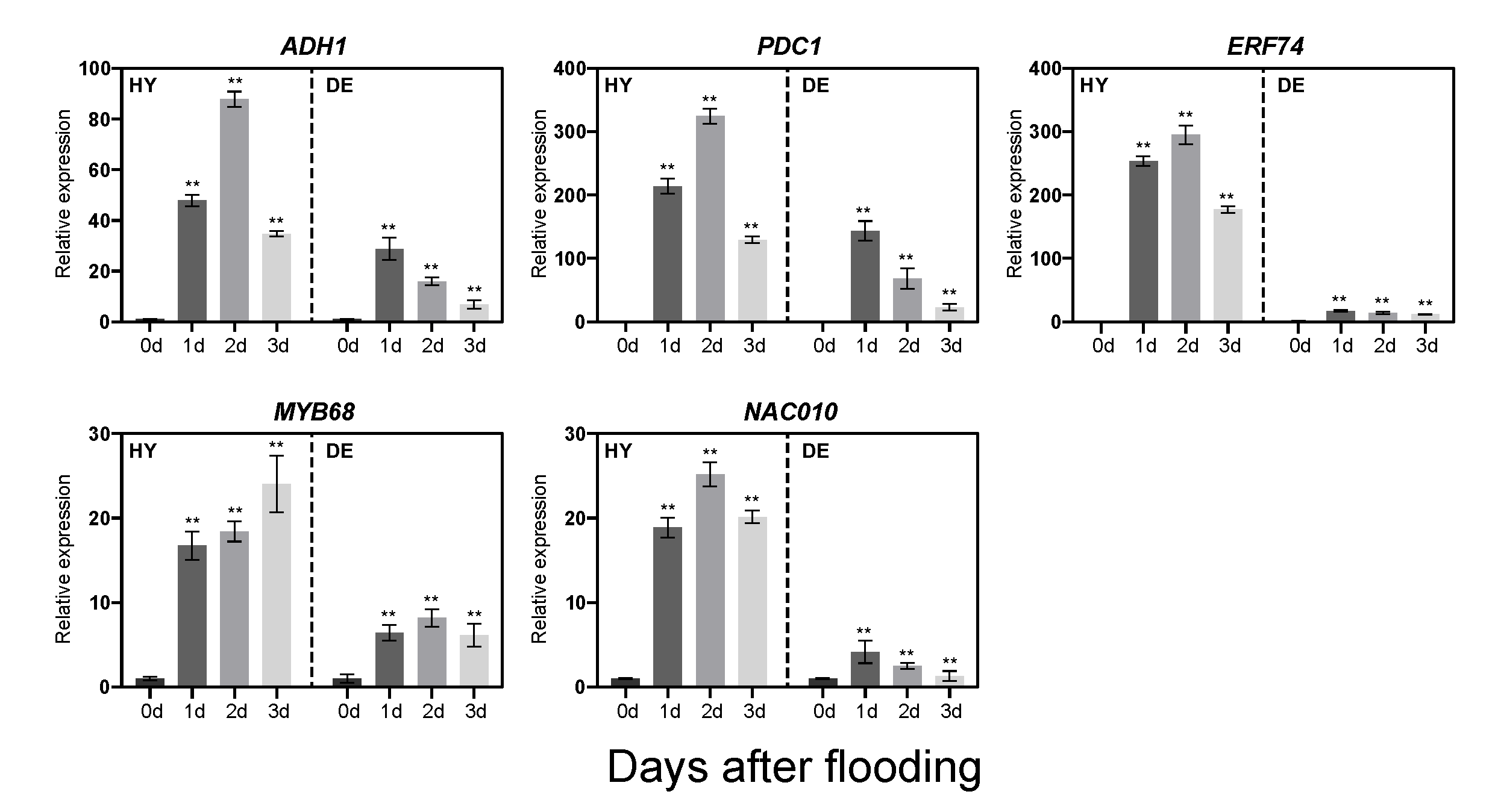
**

**Figure S17**. Expression of focused waterlogging-responsive genes under flooding stress. Data are presented as means ± SD of three independent experiments. Asterisks represent significant differences (** P < 0.01, Student’s t-test).


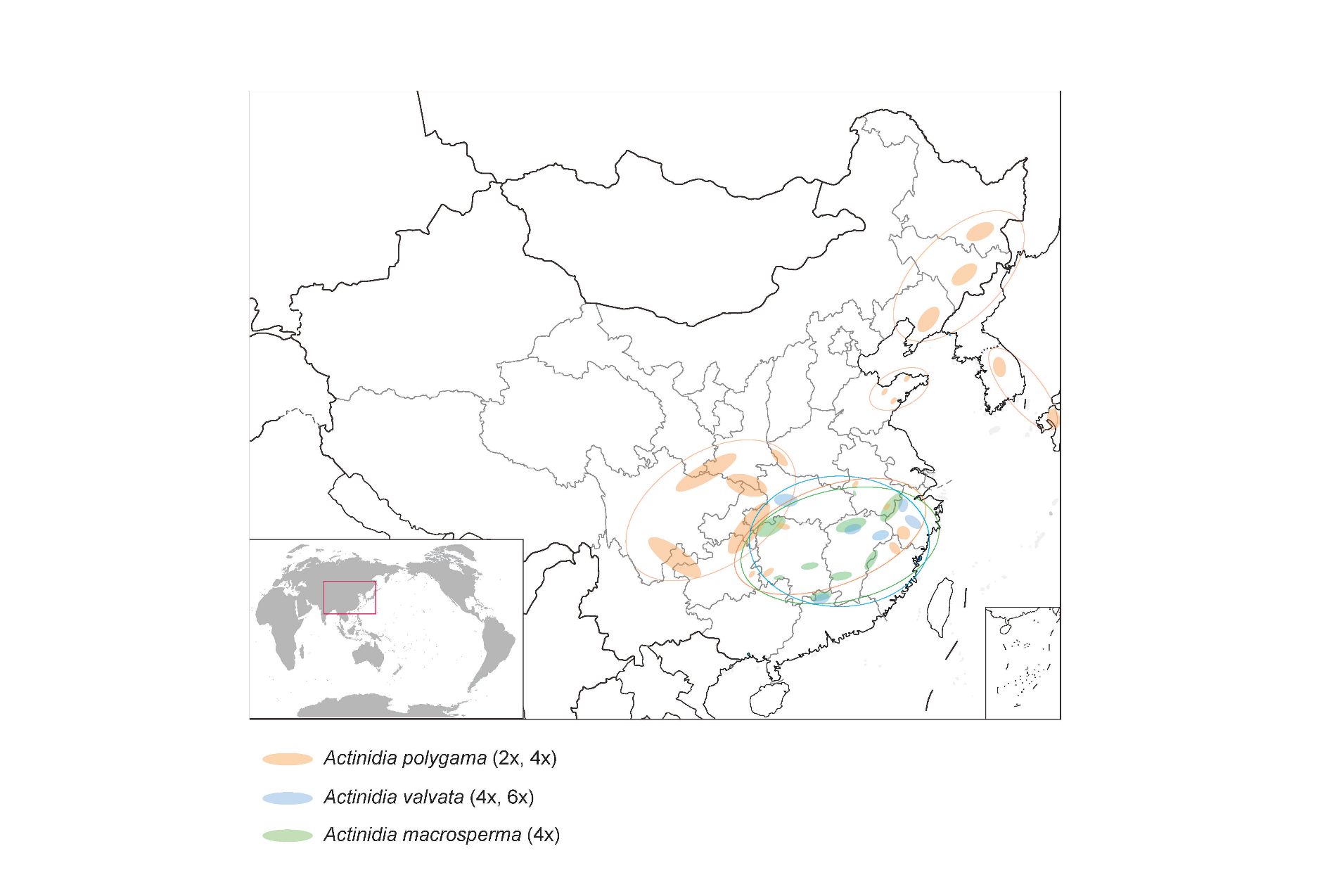


**Figure S18.** The natural geographical distribution areas of *Actinidia valvata (*tetraploid and hexaploid) and the two ancestral species *A. polygama* (diploid and tetraploid) and *A. macrosperma* (tetraploid).


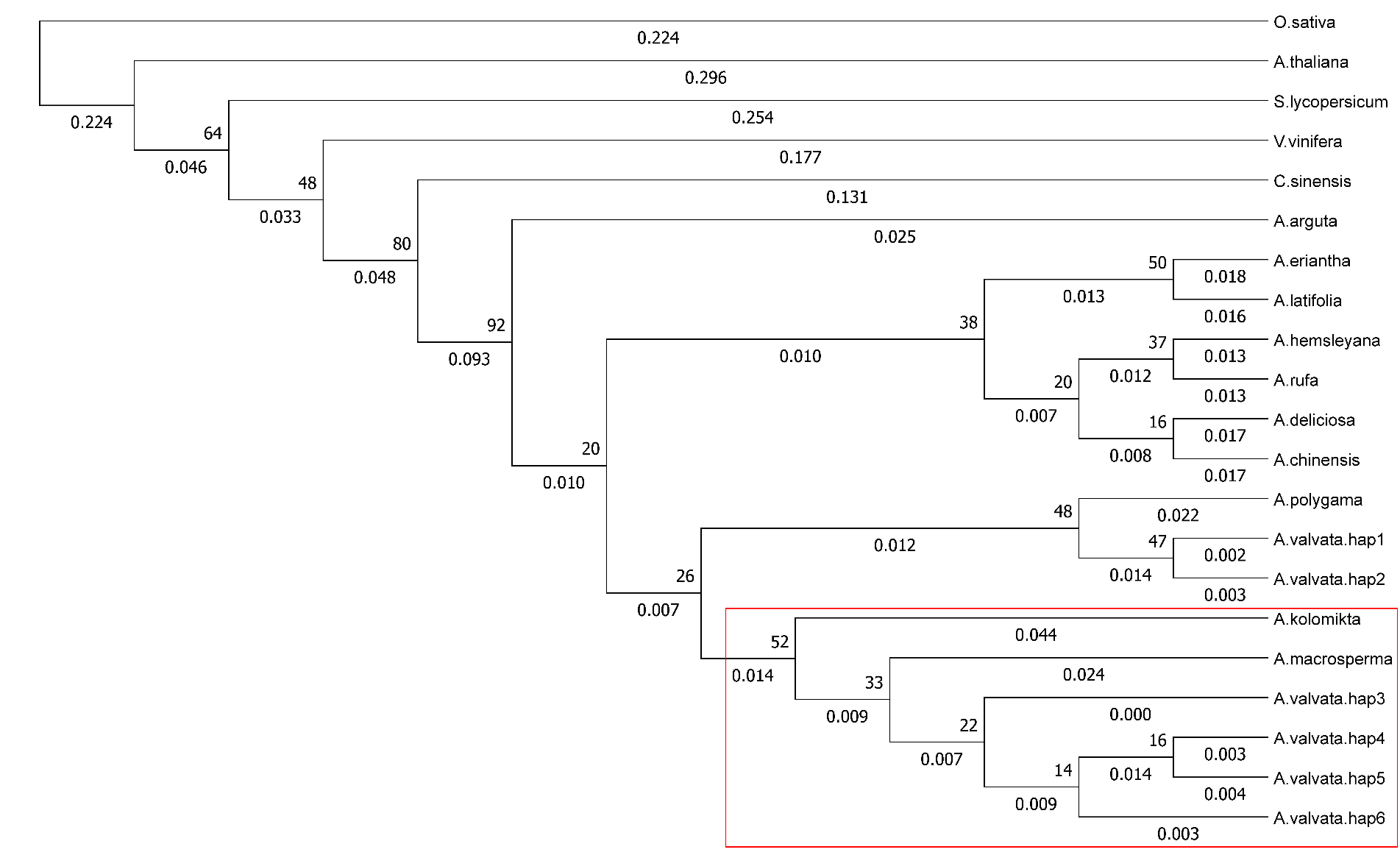


**Figure S19.** The phylogenetic tree of *Actinidia valvata* and other 15 species including re-sequenced *Actinidia kolomikta* and *Actinidia macrosperma*. The number at the node refers to bootstraps support value, and the number under branch indicates evolution distance.


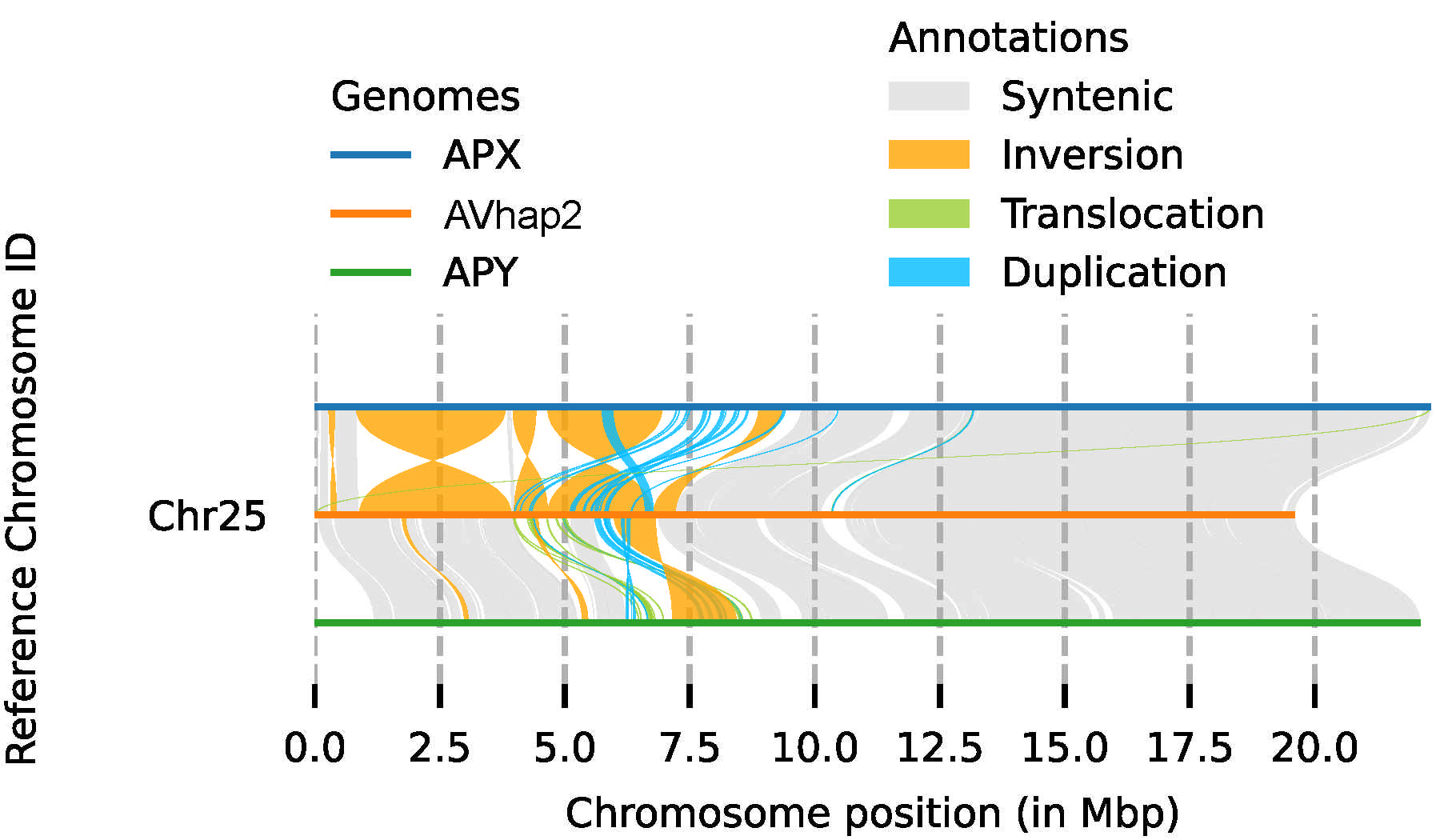


**Figure S20.** Collinearity between *Actinidia valvata* ‘DE’ haplotype 2 (AVhap2) and both X and Y chromosome of a male *A. polygama* (APX and APY).
