# Supplementary material for: Haplotype-resolved genome assembly provides insights into the evolutionary origin of waterlogging-tolerant Actinidia valvata hexaploid
Source: Hortic Res. 2026 Jan 9;13(4):uhag011. doi: 10.1093/hr/uhag011 (PMC13103481; doi:10.1093/hr/uhag011)
Supplement: Web_Material_uhag011 [file web_material_uhag011.zip › FigureS16.pdf]

ERF	MYB	NAC	WOX	WRKY	bHLH	bZIP	
5	6	15	1	9	10	1	ACO1
5	2	5	0	5	8	0	ADH1
3	0	8	1	2	11	0	CAT2
3	2	5	1	2	9	1	CIPK25
5	8	9	2	4	3	3	GASA1
5	3	4	0	8	9	3	GSTU17
1	3	5	1	0	5	0	HUP54
0	3	7	0	1	7	0	PCO2
1	3	12	0	0	7	2	PDC1
4	2	7	1	1	9	1	PYL4
2	2	13	0	2	6	1	RBOHD
0	3	4	0	5	7	0	SUS3
7	1	7	1	3	9	0	TPS11
1	1	14	0	5	10	1	XTH23
2	3	10	0	1	5	2	ABF2
7	4	13	0	2	7	4	ABI1
1	1	11	0	3	6	0	ACS1
3	4	5	0	1	3	2	ADH1_2
3	3	8	0	4	4	0	ADHL2
1	0	4	0	1	7	0	CAT2_2
3	5	5	2	4	6	2	CAT2_3
3	2	5	0	0	8	1	CIPK25_2
2	2	5	2	3	4	0	DEAR3
2	8	8	0	4	7	1	GASA1_2
4	1	13	0	3	2	1	GSTU1
2	4	9	0	2	4	0	GSTU19
2	7	5	0	1	8	0	GSTU25
5	4	3	2	5	3	0	GSTU7_1
4	3	11	1	1	3	1	GSTU7_2
4	1	7	0	1	6	1	GSTU8_1
0	1	13	0	1	6	2	GSTU8_2
1	4	17	0	1	11	0	GSTU8_3
1	6	12	0	3	6	0	HUP54_2
1	6	21	1	10	9	7	IAA13
1	5	15	1	7	6	2	IAA17
4	3	14	0	4	6	0	IAA17_2
2	2	10	2	1	8	0	KCS1
1	8	10	2	4	6	0	KCS3
0	2	11	0	2	2	0	PAL1
4	2	23	1	6	7	2	PAL2
1	6	16	1	5	6	1	PCO2_2
2	2	7	0	1	3	2	PDC2
2	3	4	0	4	4	1	PP2C38
7	3	7	0	2	7	0	PP2C49
2	4	11	0	1	7	1	PYL1
2	4	11	0	1	9	0	PYL4_2
4	4	10	0	0	9	1	PYL4_3
3	2	13	2	5	4	1	SD1-13
2	1	7	1	3	1	4	SOD1
2	5	11	0	2	3	4	SOS2
1	7	15	0	3	4	0	SOS4
5	14	14	1	2	10	0	TPS11_2
2	4	6	1	6	6	0	TPS5
3	5	10	0	1	9	0	TPS7
3	4	15	0	3	9	7	TPS7_2
1	12	5	0	3	19	0	TPS9
0	2	7	1	3	10	1	XTH23_2
6	1	9	1	1	1	0	XTH28
2	3	11	0	3	5	0	XTH30
3	4	4	2	1	10	0	XTH32

ERF	MYB	NAC	WOX	WRKY	bHLH	bZIP	
4	7	9	0	5	7	0	bHLH093
1	5	15	0	0	5	3	bHLH105
2	2	1	0	2	4	1	bZIP21
0	4	9	0	4	10	0	bZIP23
3	11	13	1	6	9	3	bZIP62
5	2	17	1	0	10	1	ERF002
4	3	6	0	7	5	0	ERF019
1	4	18	1	1	5	3	ERF041
2	7	11	1	5	6	3	ERF041_2
3	6	15	0	3	13	1	ERF059
2	7	11	2	4	8	1	ERF072
4	3	6	0	1	6	1	ERF074
3	1	8	0	4	6	1	ERF075
5	3	15	1	2	2	3	ERF078
2	4	11	1	4	15	1	ERF078_2
9	1	13	0	2	7	1	ERF081
4	2	14	0	2	4	2	ERF082
0	2	18	0	4	12	2	ERF095
2	3	13	1	4	7	3	ERF100
3	2	22	1	0	9	2	ERF100_2
3	4	9	2	3	5	0	ERF102
4	3	23	0	1	3	2	ERF102_2
4	3	14	1	5	12	0	ERF106
3	6	22	1	4	12	0	ERF106_2
8	5	6	1	5	2	0	ERF110
1	2	9	1	2	12	1	ERF1-3
1	1	12	0	2	9	1	ERF48
3	6	11	0	6	12	0	MYB116
2	1	9	3	2	7	1	MYB7
1	4	6	1	1	7	0	MYB7_2
4	11	18	1	1	4	1	MYB73_1
4	12	17	1	1	5	1	MYB73_2
1	4	9	0	3	9	0	MYB78_1
2	2	6	0	0	2	0	MYB78_2
3	1	16	1	1	2	3	MYB86
3	3	7	1	1	11	1	MYB94
1	2	17	0	3	11	2	NAC010
2	5	8	0	3	5	0	NAC071
2	8	14	0	3	8	0	NAC78
3	1	10	0	2	3	2	WOX11
2	4	16	0	2	4	1	WOX13
3	5	7	0	2	6	1	WRKY14
2	3	4	0	1	3	1	WRKY22
6	3	11	0	3	5	3	WRKY33
0	4	8	3	8	7	0	WRKY33_2
2	5	13	1	4	3	1	WRKY40
4	3	20	1	3	11	1	WRKY53
0	3	16	1	3	4	1	WRKY57
2	2	8	1	5	10	0	WRKY72
2	4	26	0	4	9	0	WRKY75
