# Supplementary figures and images for: Haplotype-resolved genome assembly provides insights into the evolutionary origin of waterlogging-tolerant Actinidia valvata hexaploid
Source: Hortic Res. 2026 Jan 9;13(4):uhag011. doi: 10.1093/hr/uhag011 (PMC13103481; doi:10.1093/hr/uhag011)

**ERF gene family**

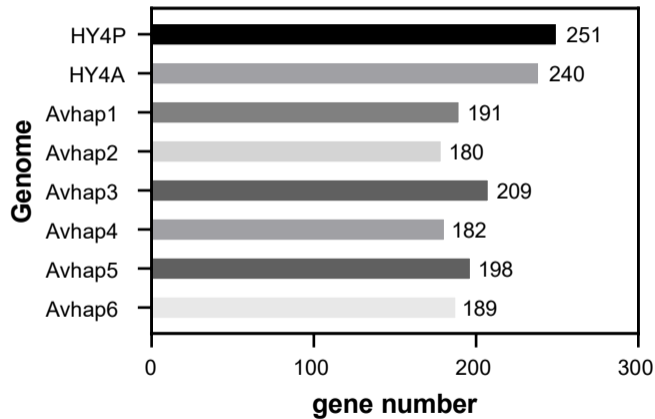

**NAC gene family**

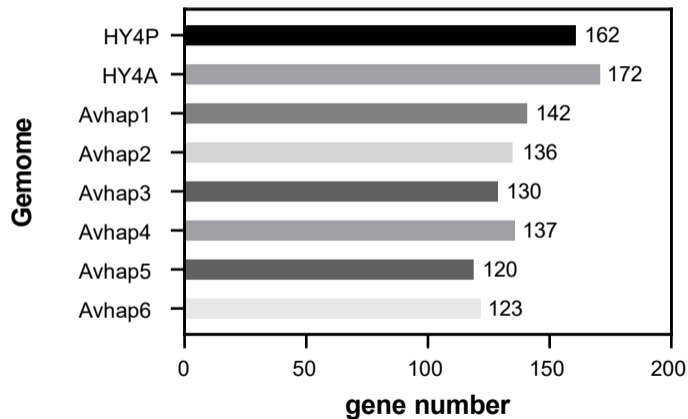

Supplement: Web_Material_uhag011 [file web_material_uhag011.zip › FigureS10_new.pdf]

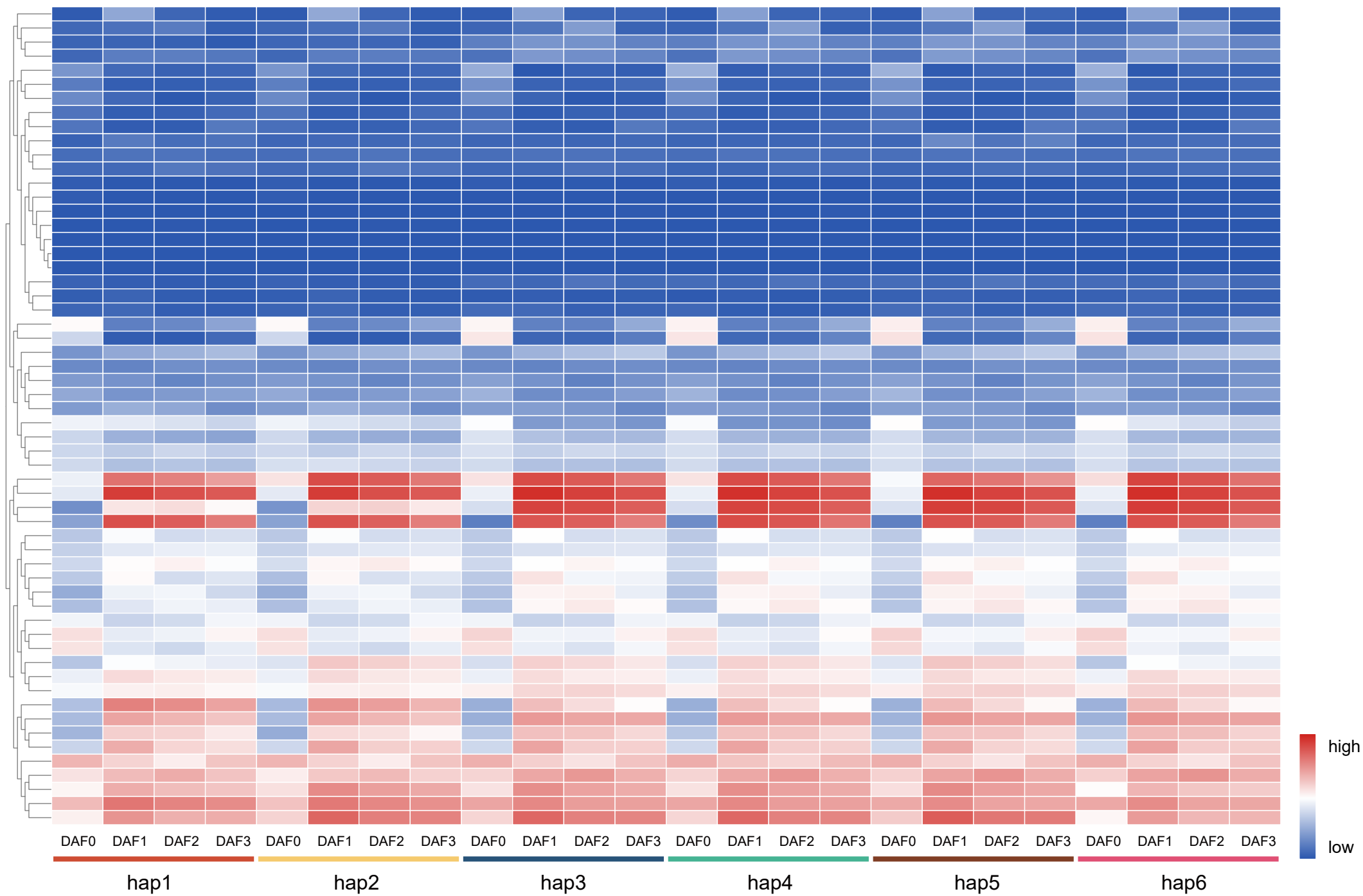

Supplement: Web_Material_uhag011 [file web_material_uhag011.zip › FigureS11.pdf]

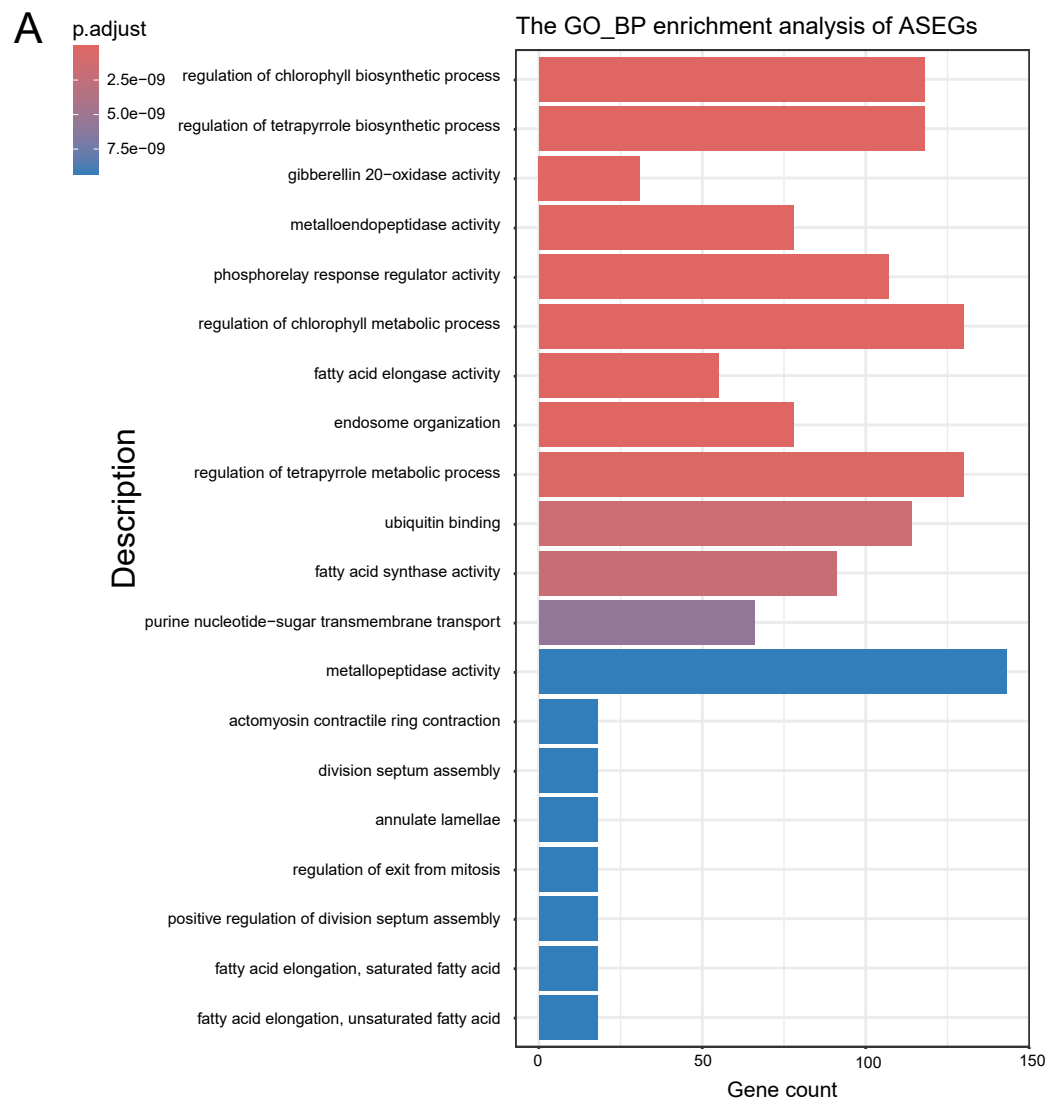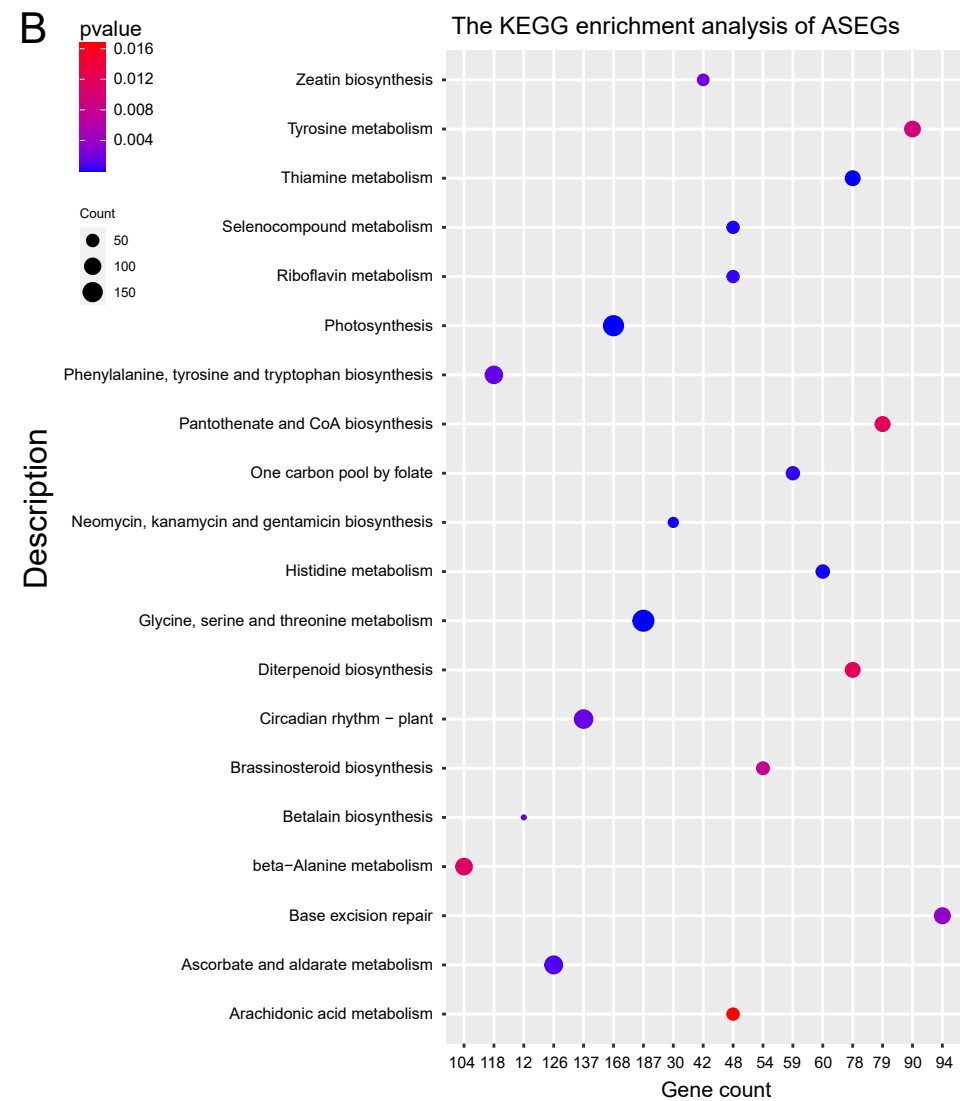

Supplement: Web_Material_uhag011 [file web_material_uhag011.zip › FigureS12.pdf]

A

Sample clustering to detect outliers

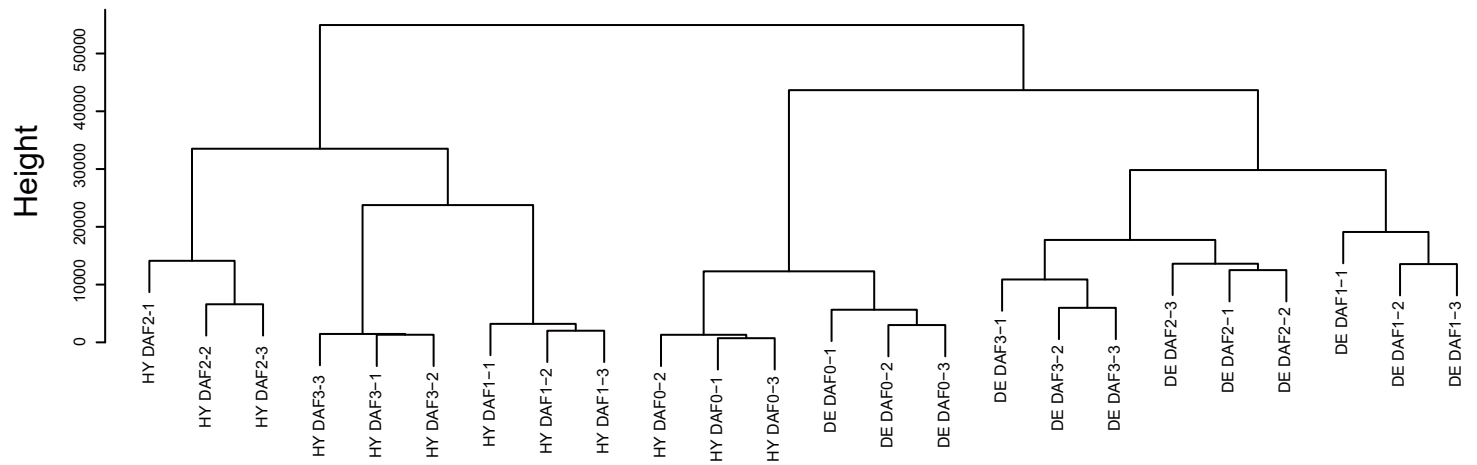

B

Module-trait relationships

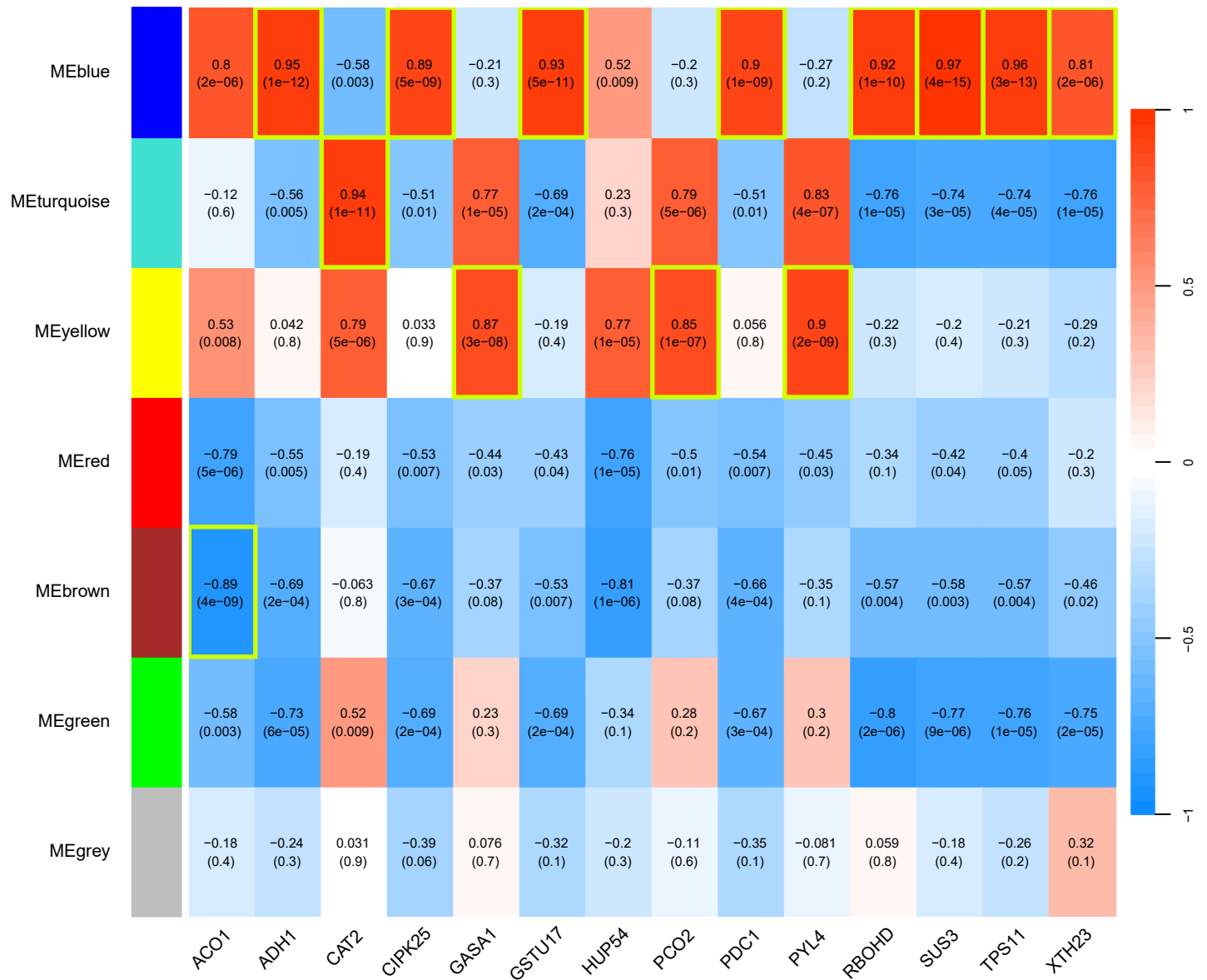

Supplement: Web_Material_uhag011 [file web_material_uhag011.zip › FigureS13.pdf]

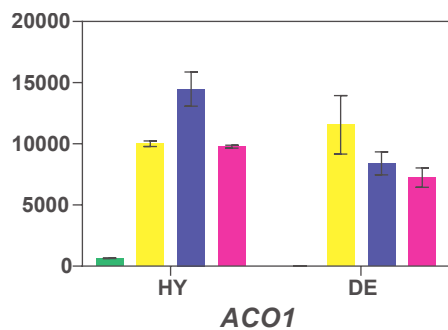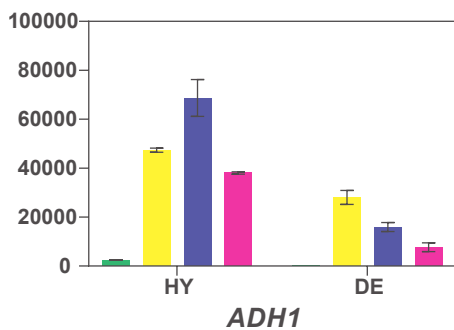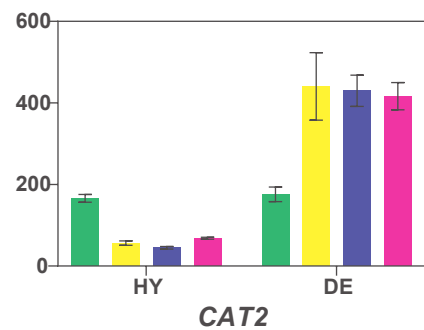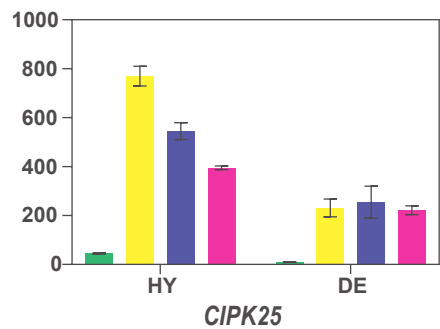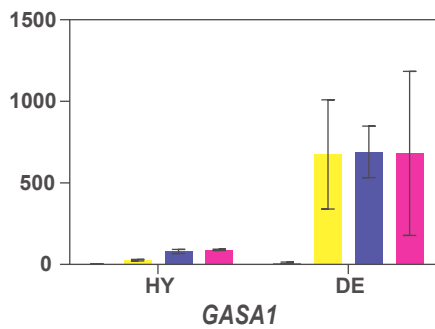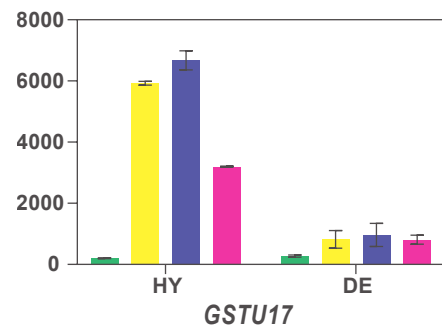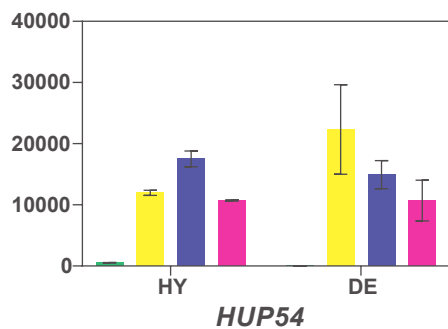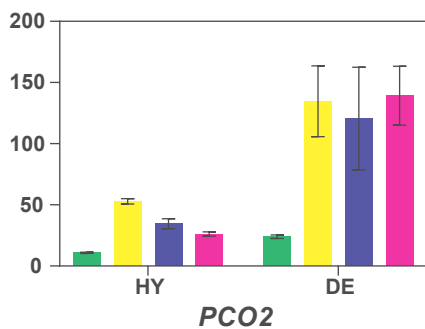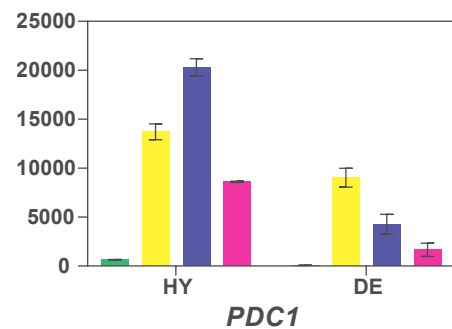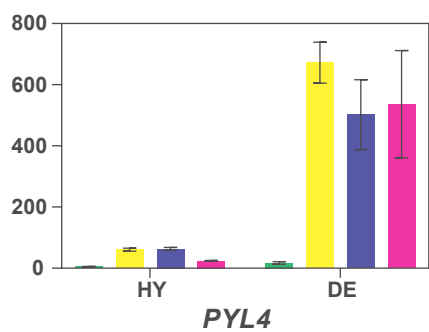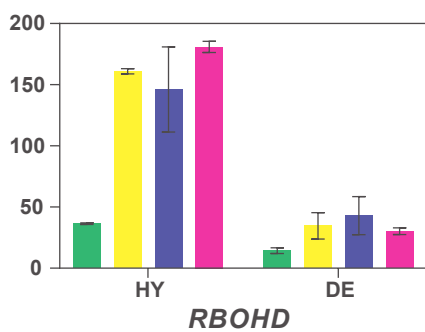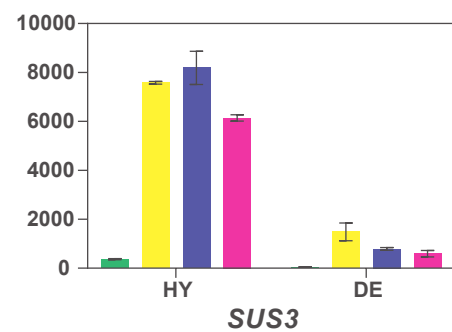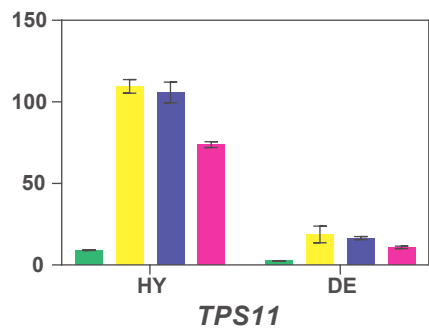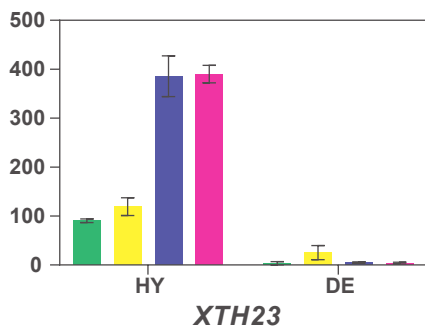

DAF0 DAF2  
DAF1 DAF3

Supplement: Web_Material_uhag011 [file web_material_uhag011.zip › FigureS14.pdf]

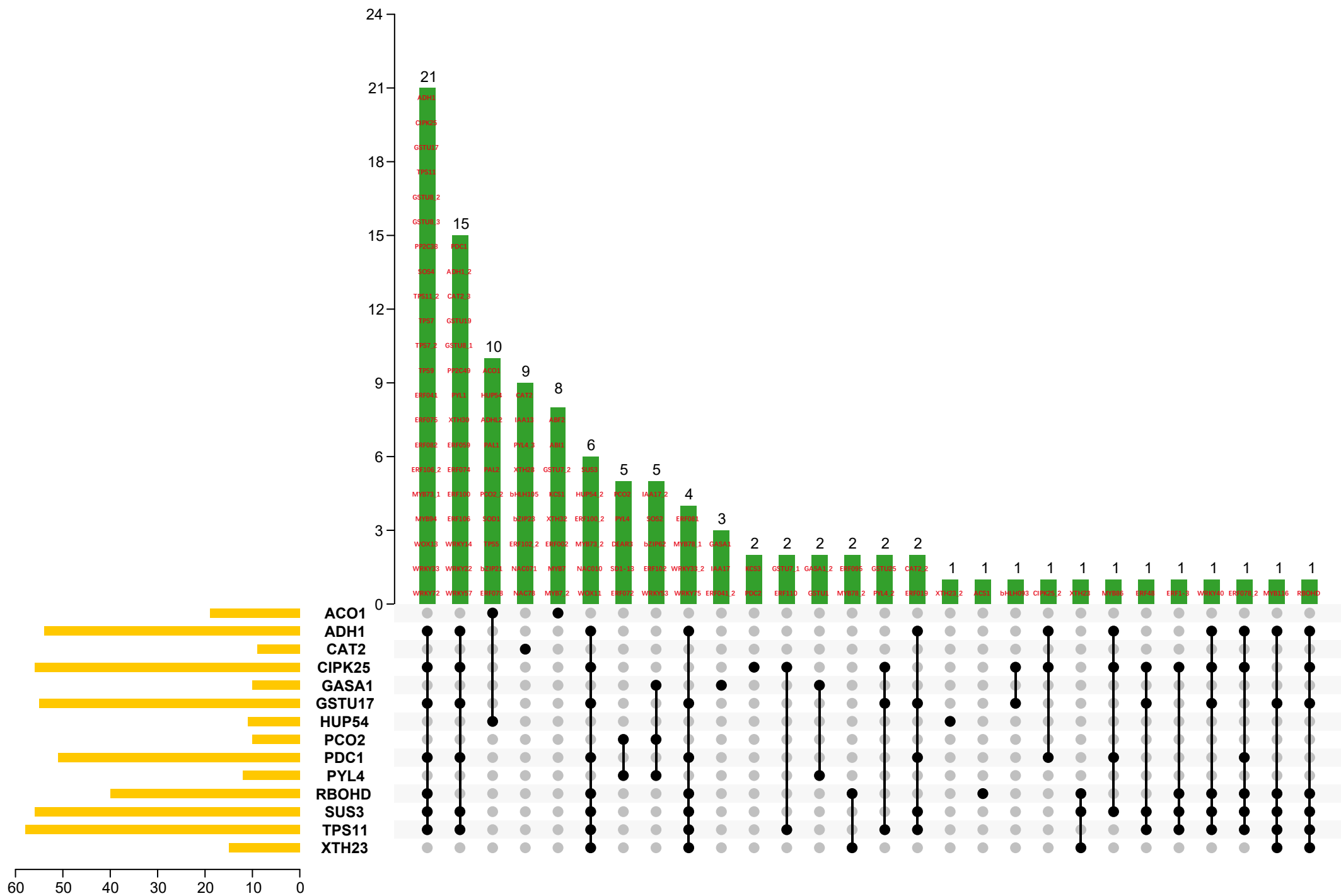

Supplement: Web_Material_uhag011 [file web_material_uhag011.zip › FigureS15.pdf]

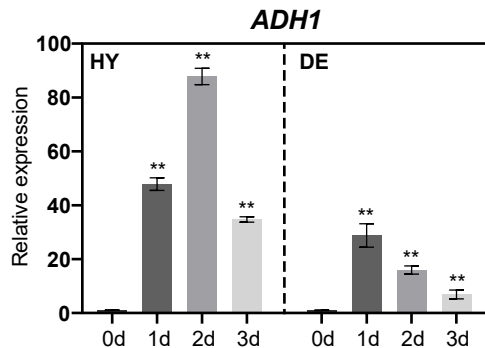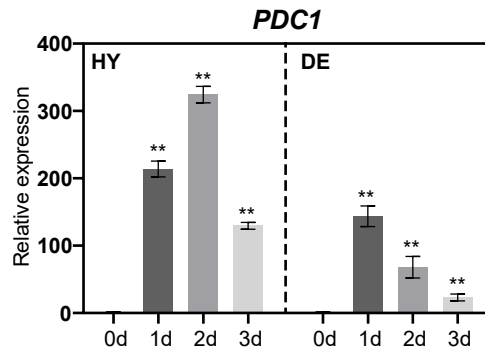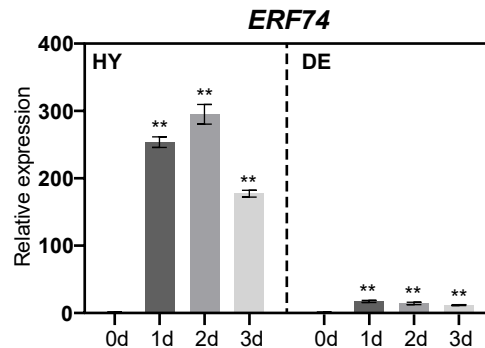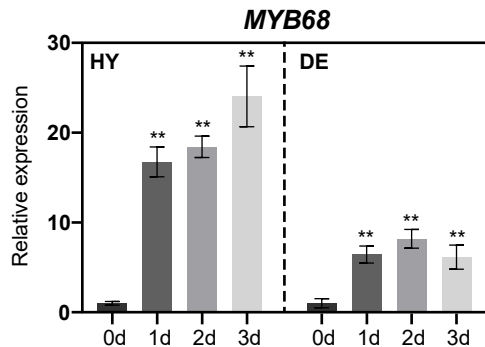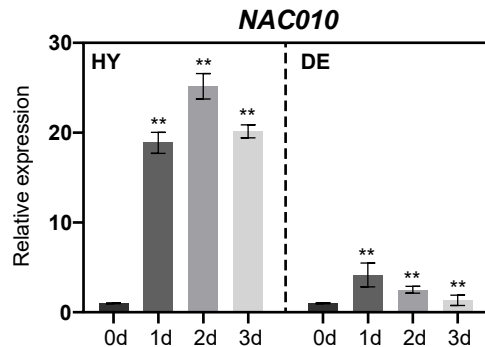

Days after flooding

Supplement: Web_Material_uhag011 [file web_material_uhag011.zip › FigureS17_new.pdf]

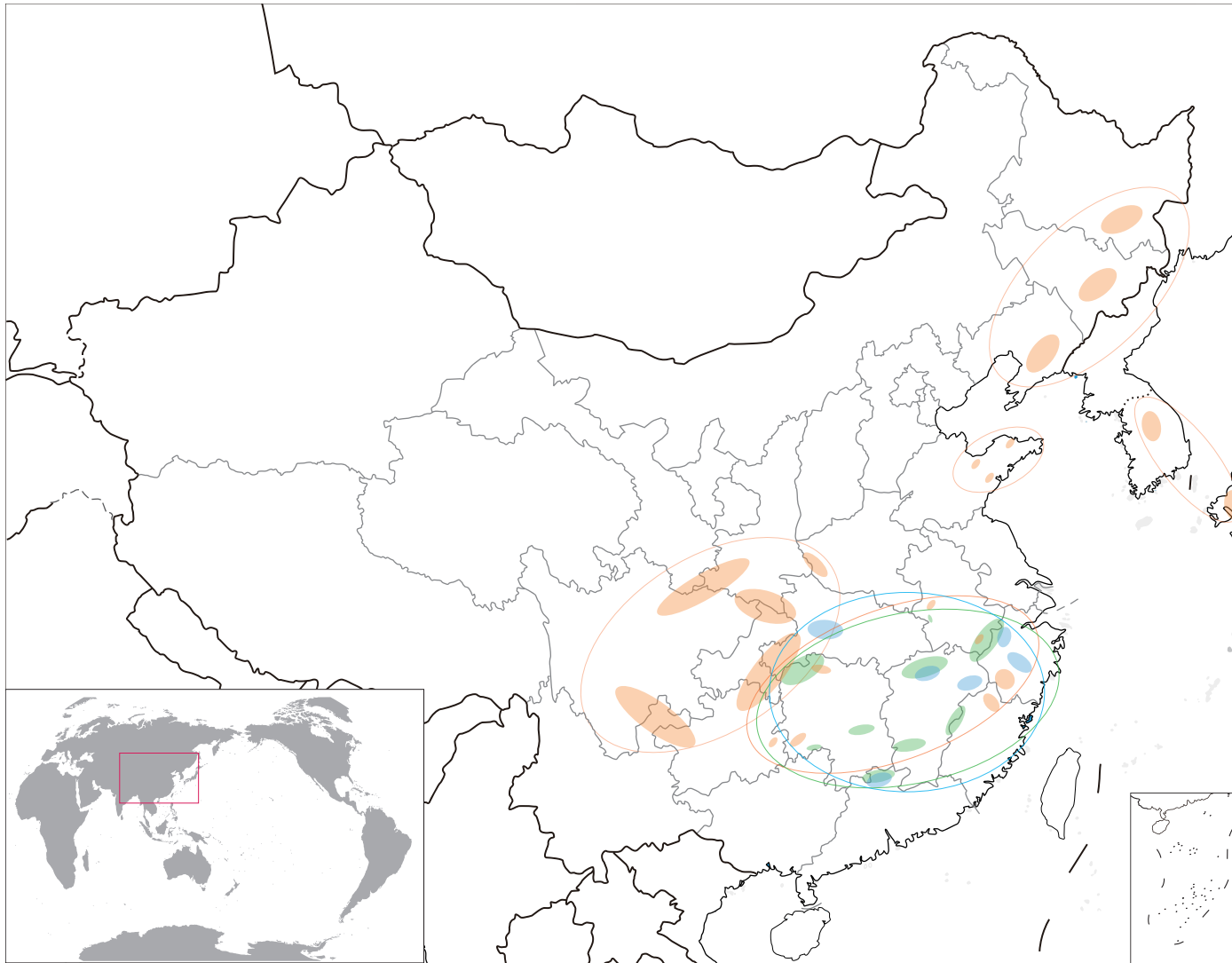

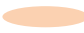 *Actinidia polygama* (2x, 4x)

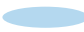 *Actinidia valvata* (4x, 6x)

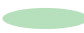 *Actinidia macrosperma* (4x)

Supplement: Web_Material_uhag011 [file web_material_uhag011.zip › FigureS18.pdf]

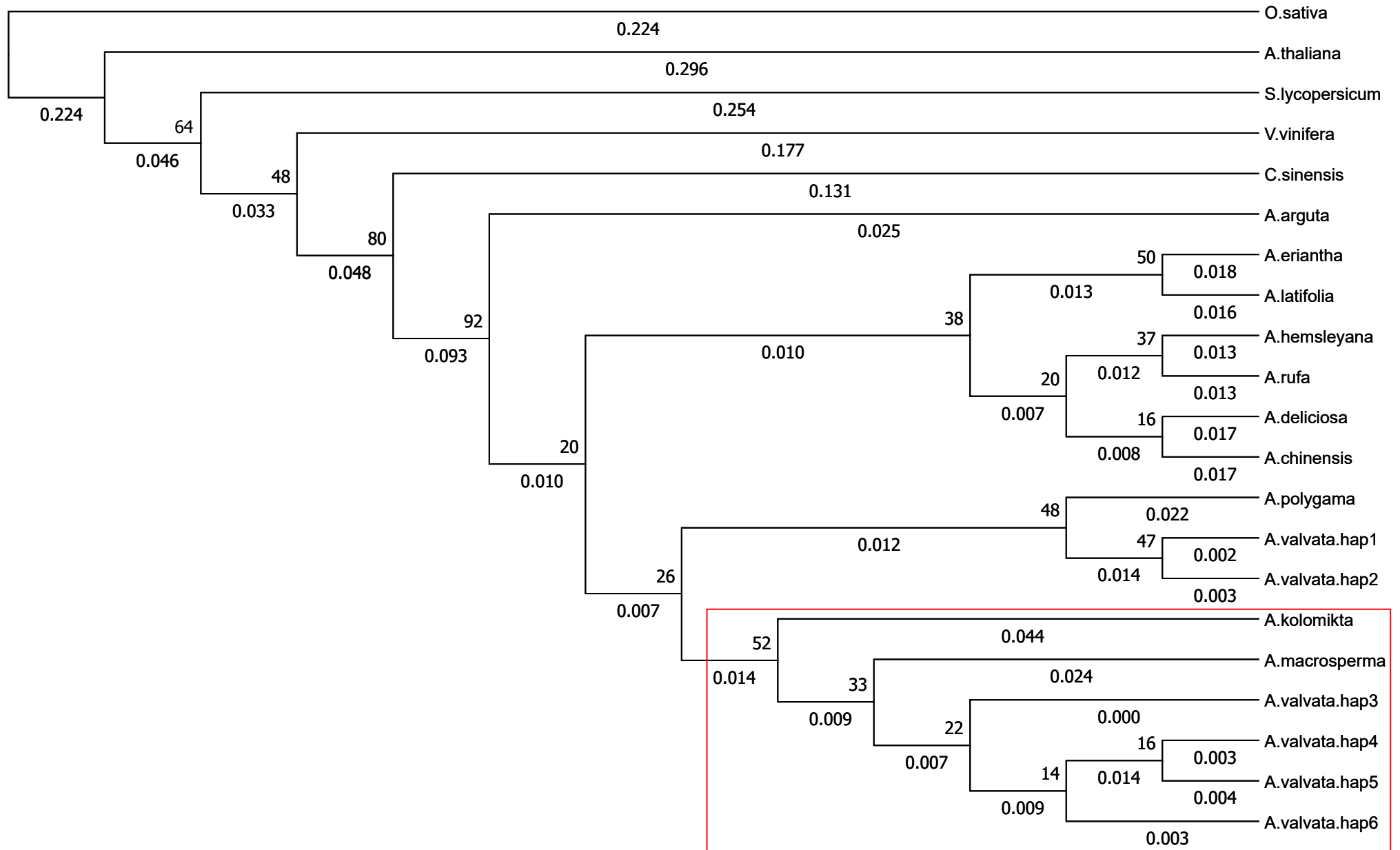

Supplement: Web_Material_uhag011 [file web_material_uhag011.zip › FigureS19.pdf]

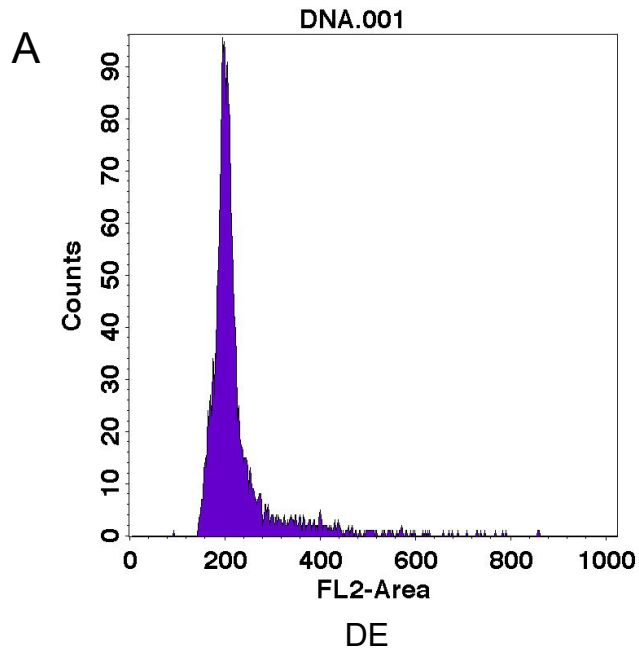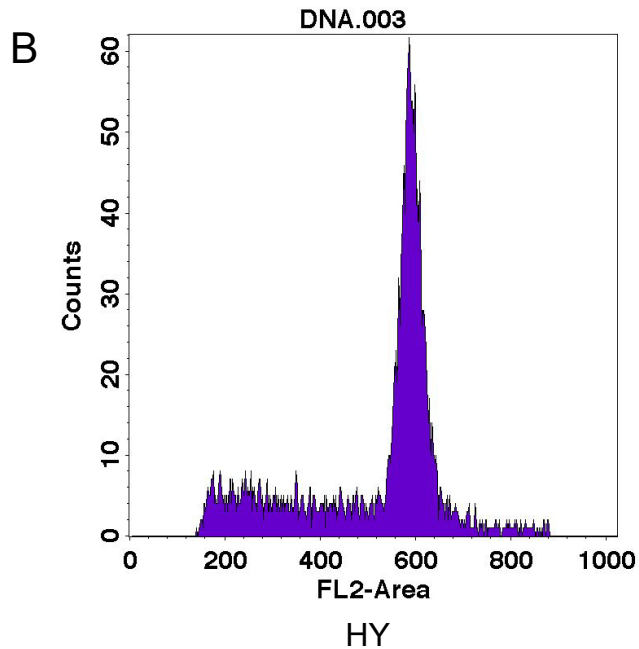

Supplement: Web_Material_uhag011 [file web_material_uhag011.zip › FigureS1_new.pdf]

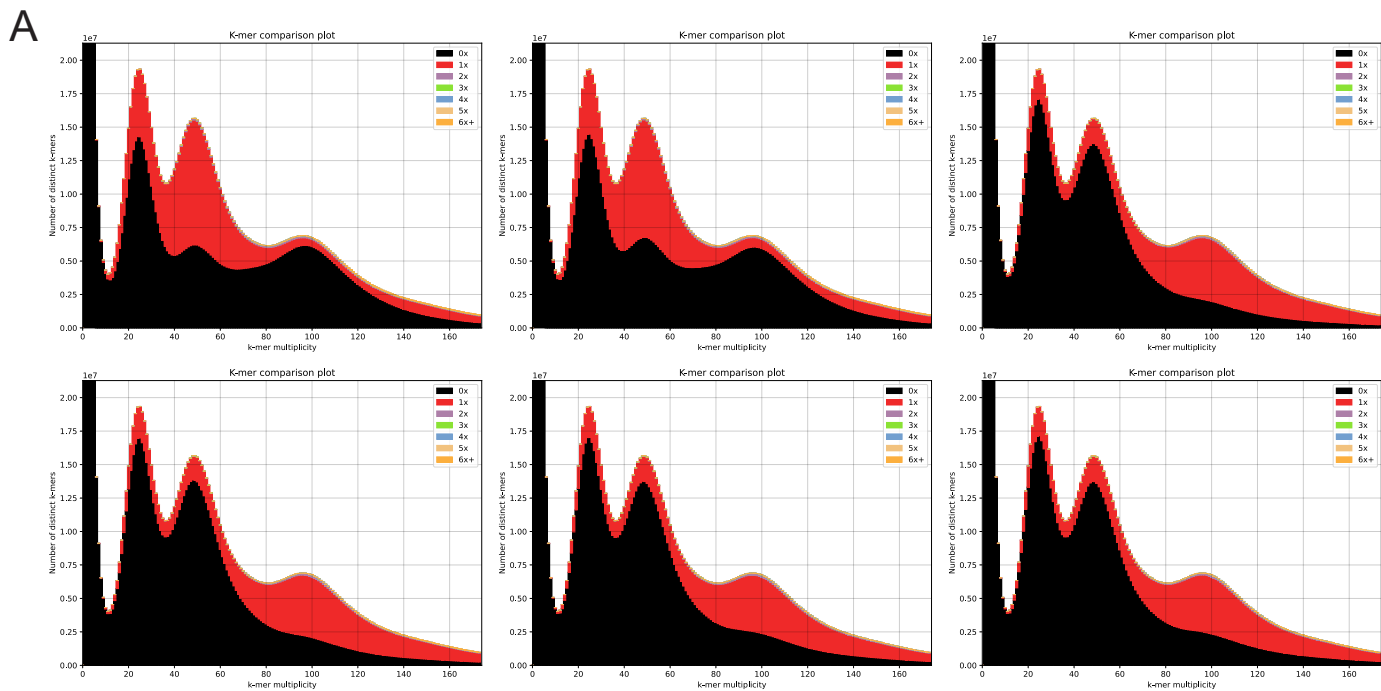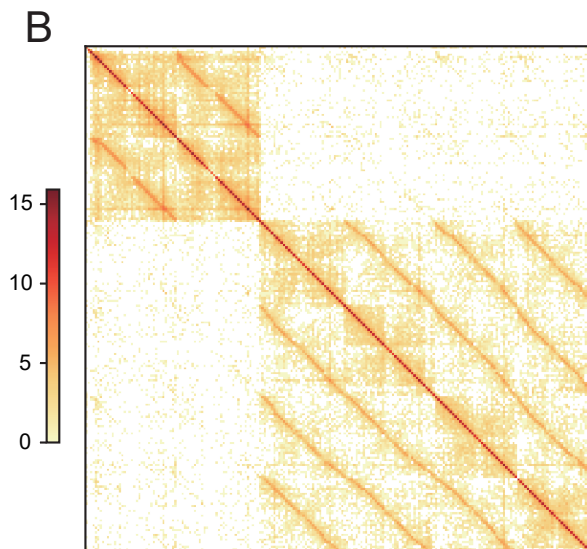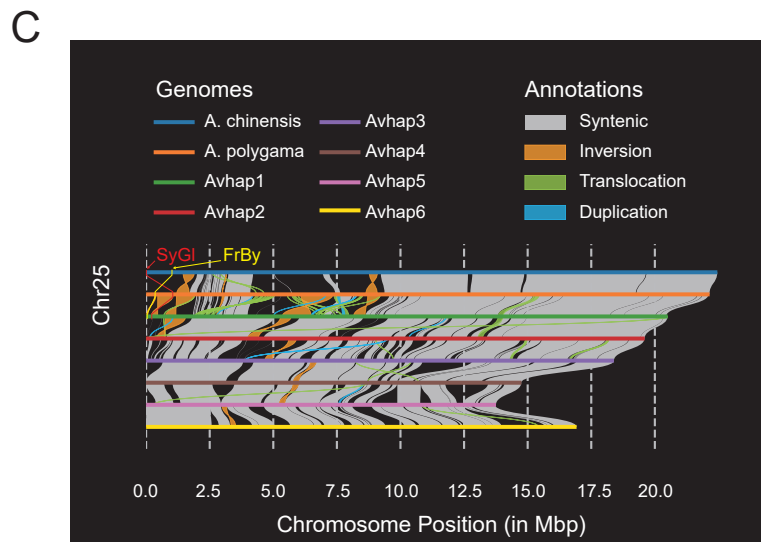

Supplement: Web_Material_uhag011 [file web_material_uhag011.zip › FigureS2.pdf]

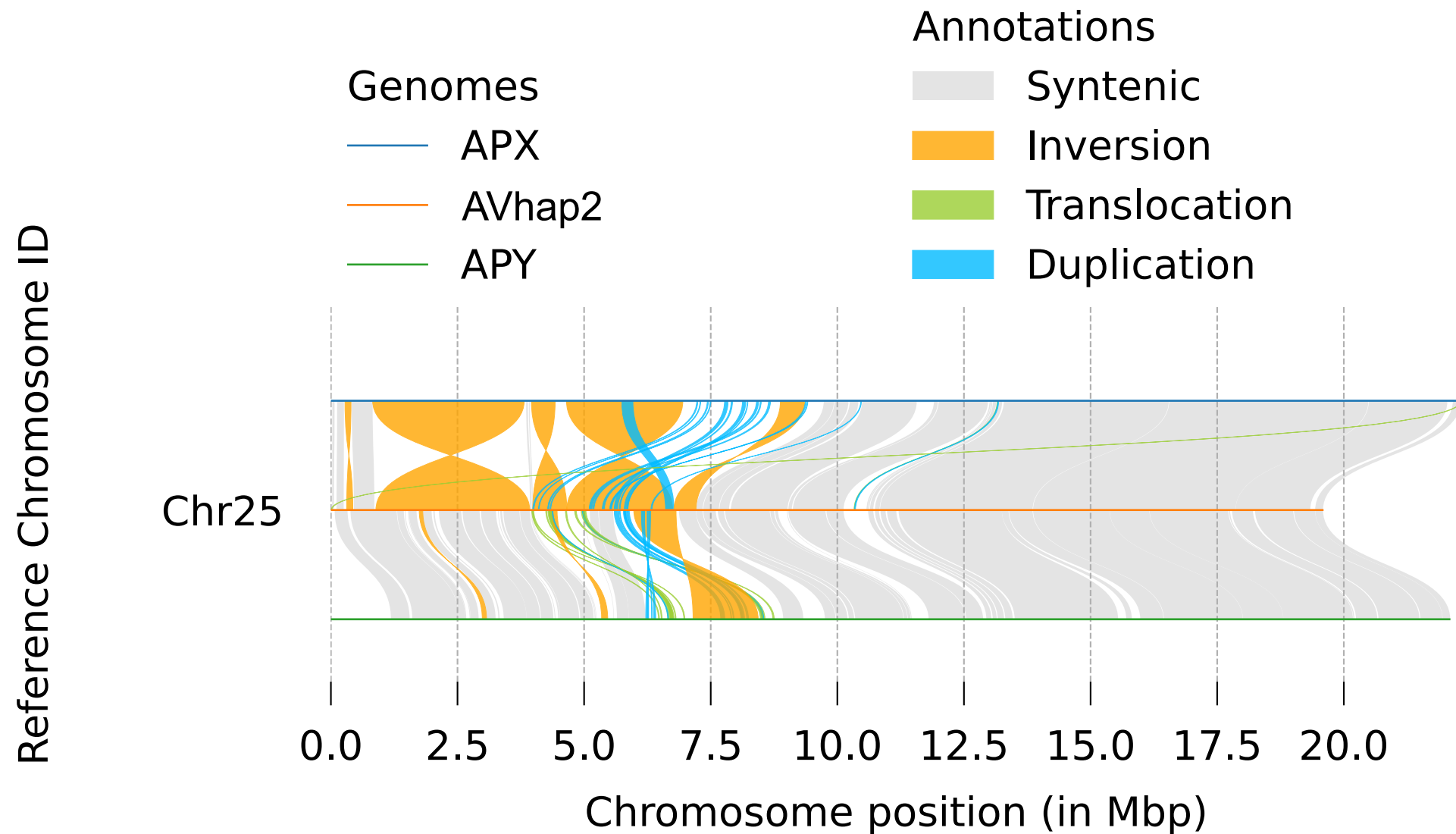

Supplement: Web_Material_uhag011 [file web_material_uhag011.zip › FigureS20.pdf]

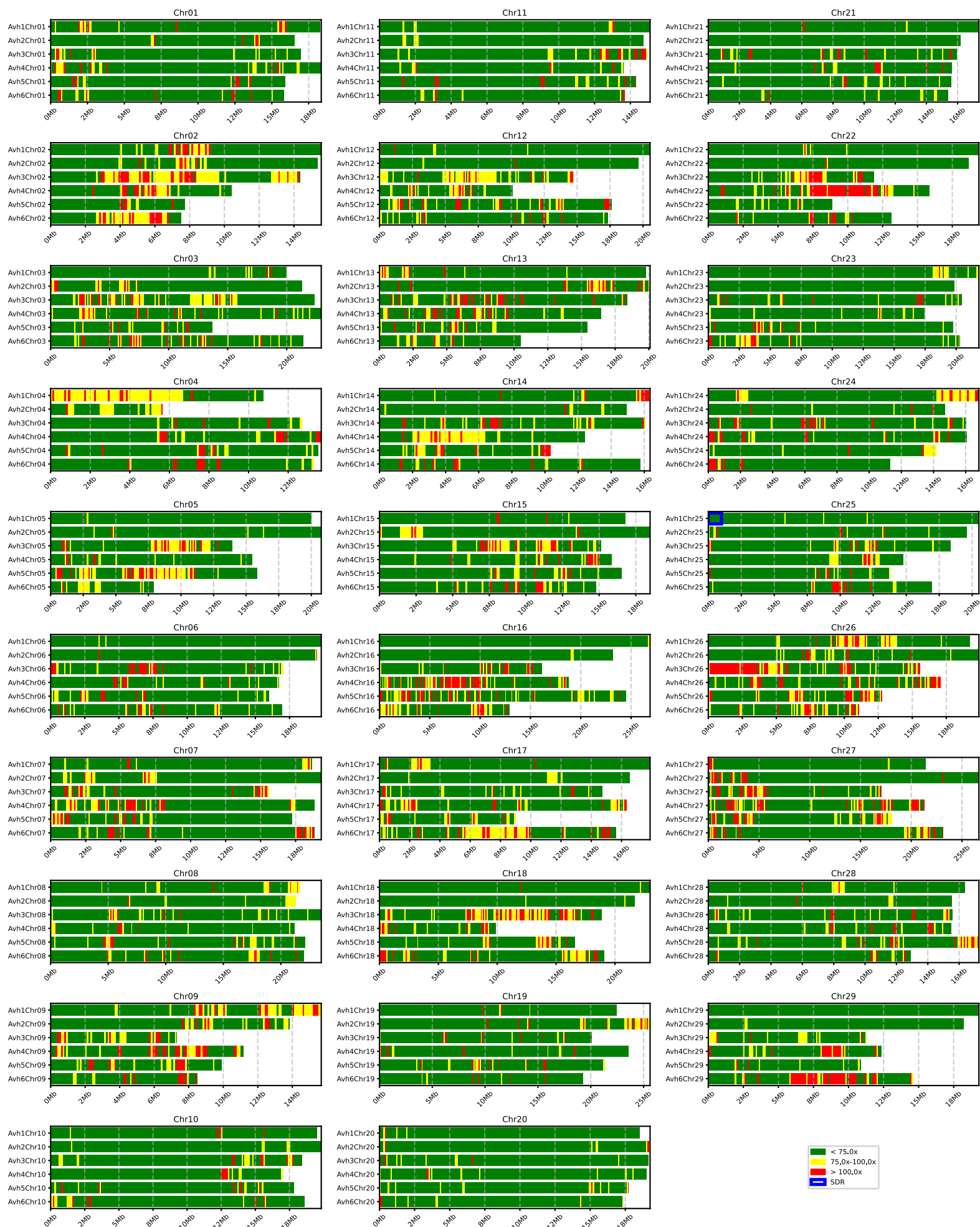

Supplement: Web_Material_uhag011 [file web_material_uhag011.zip › FigureS3.pdf]

A

## Expand

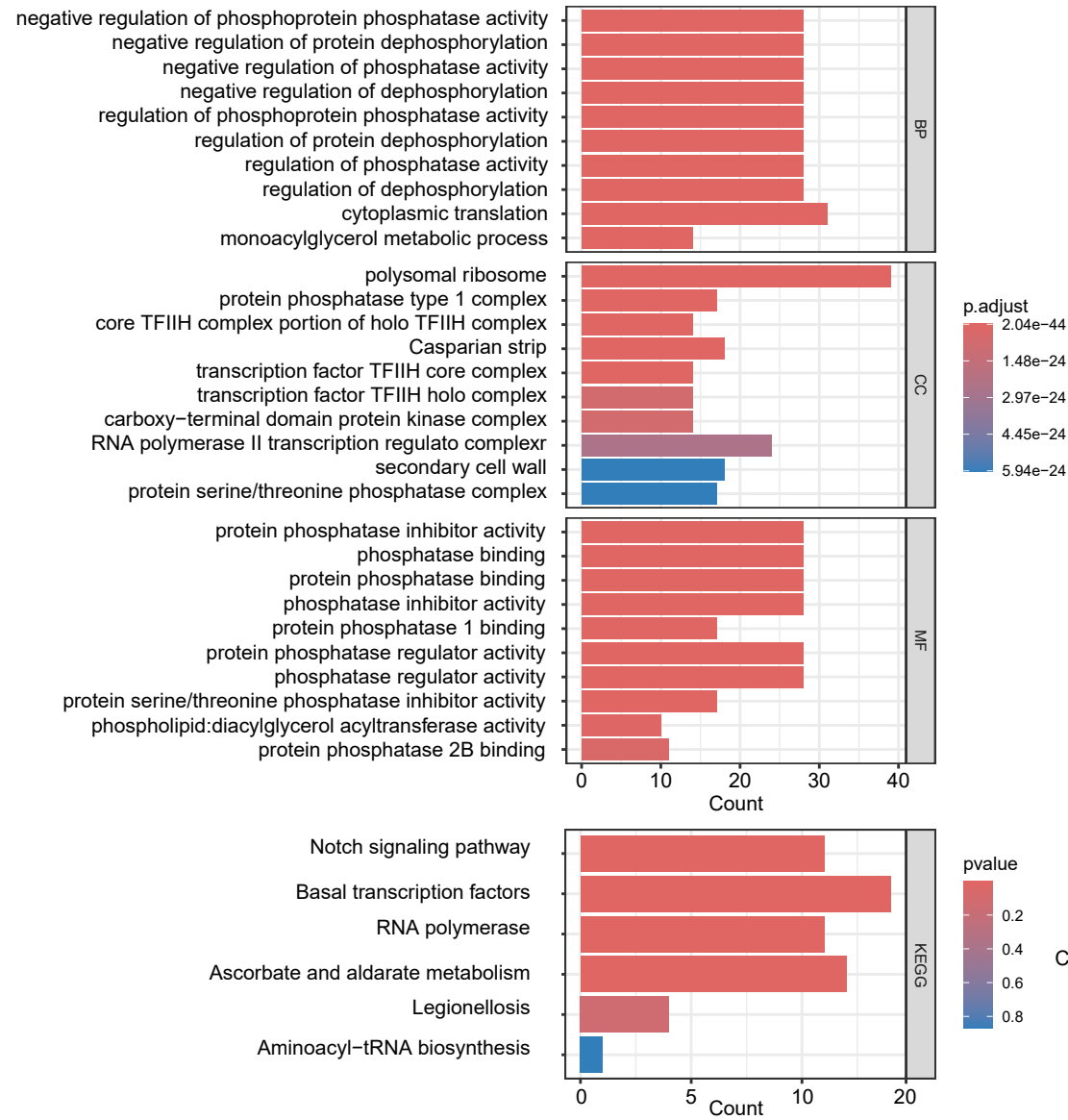

B

## Contract

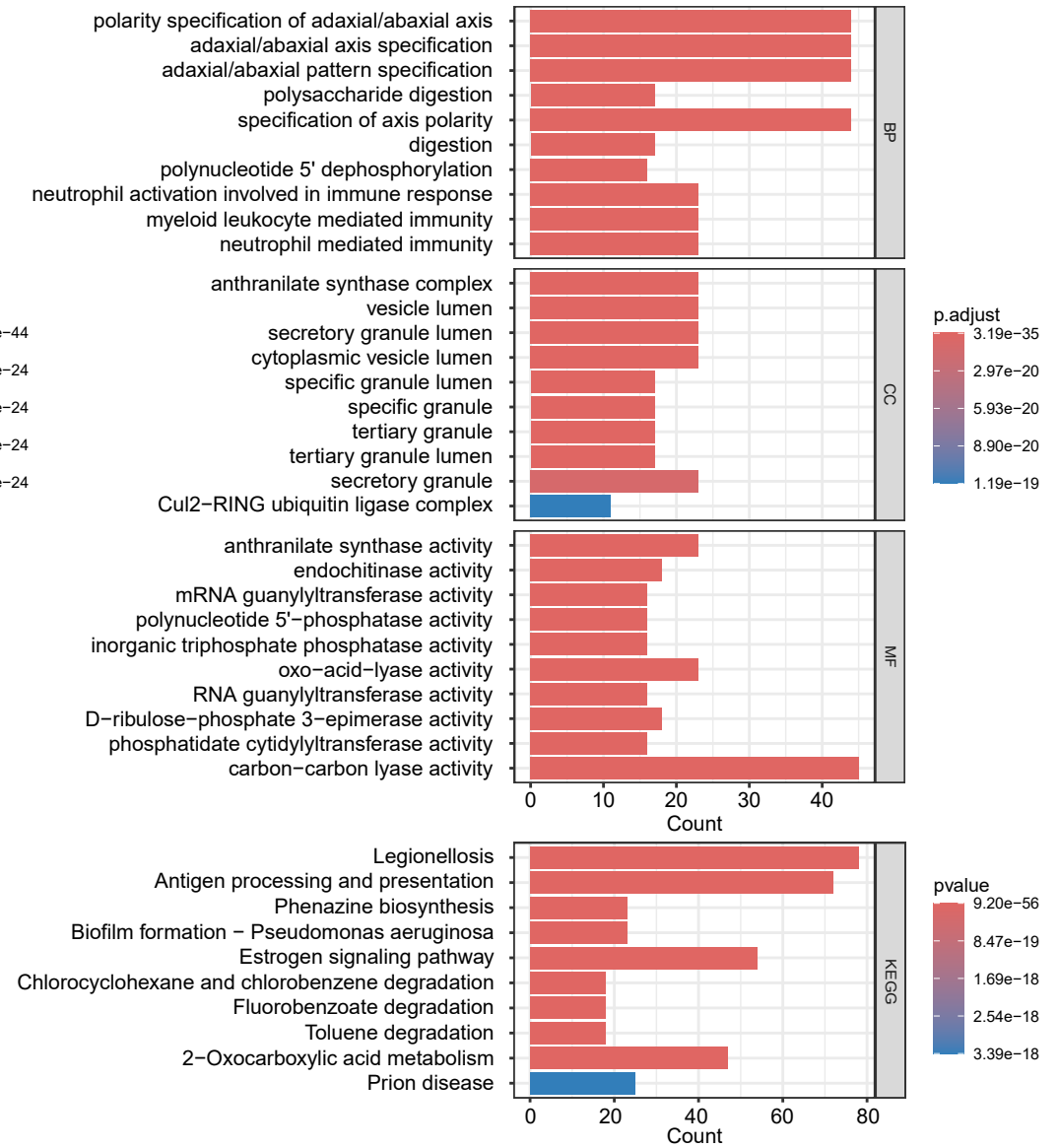

Supplement: Web_Material_uhag011 [file web_material_uhag011.zip › FigureS4.pdf]

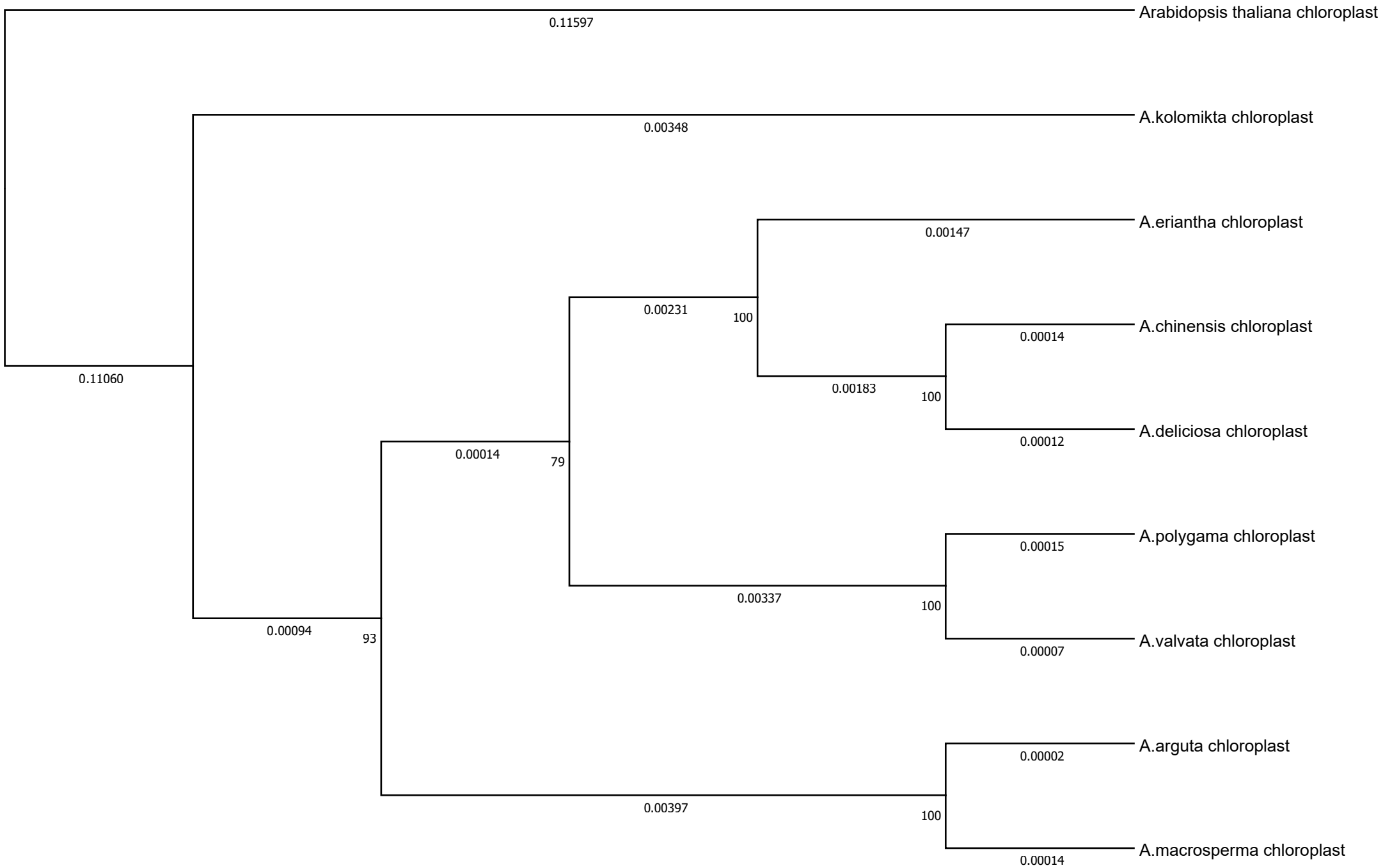

Supplement: Web_Material_uhag011 [file web_material_uhag011.zip › FigureS5.pdf]

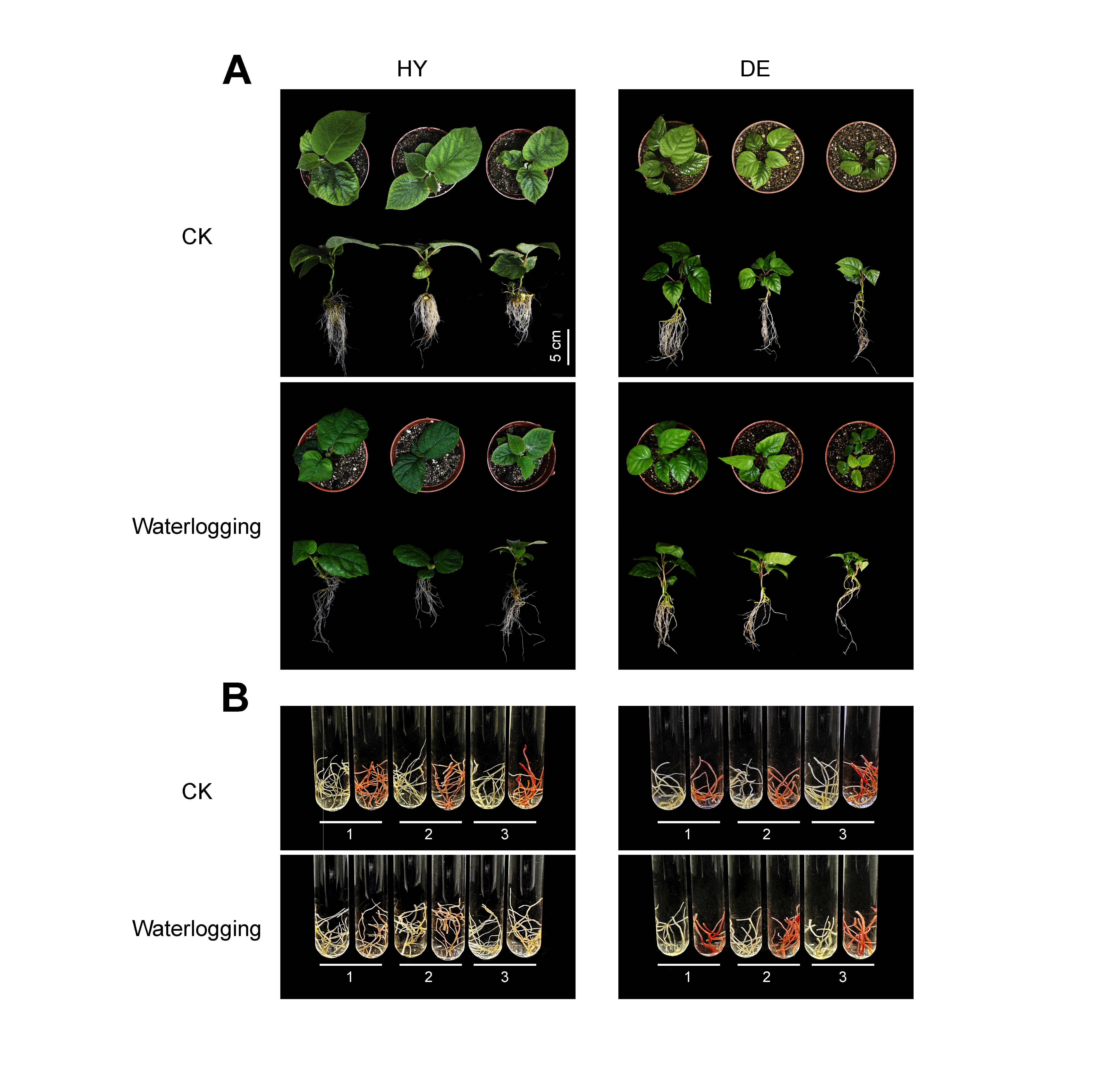

Supplement: Web_Material_uhag011 [file web_material_uhag011.zip › FigureS6_new.tiff]

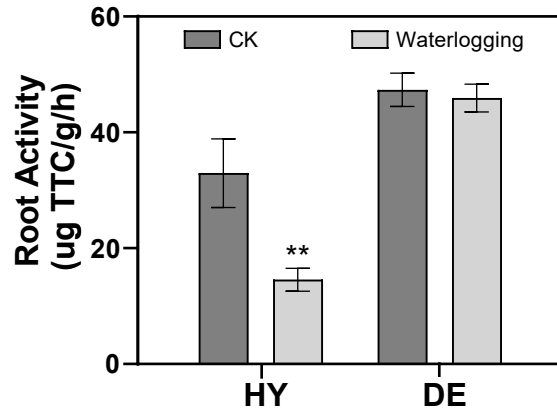

Supplement: Web_Material_uhag011 [file web_material_uhag011.zip › FigureS7_new.pdf]

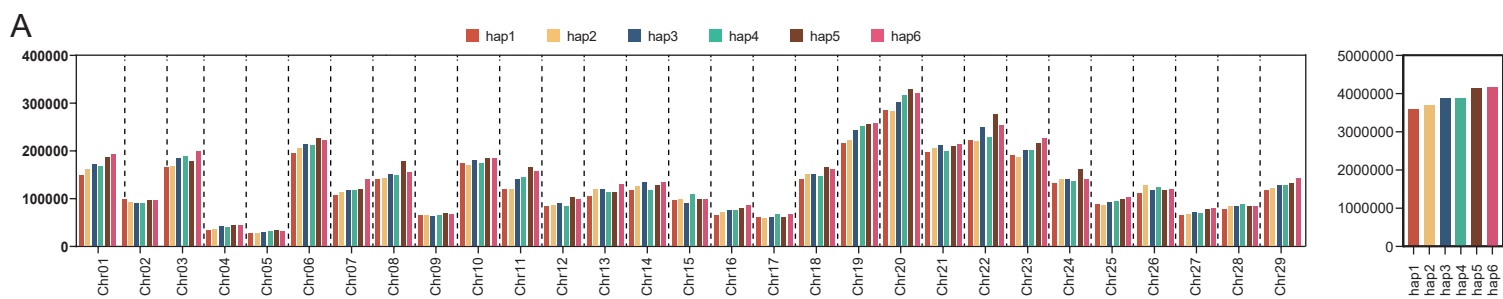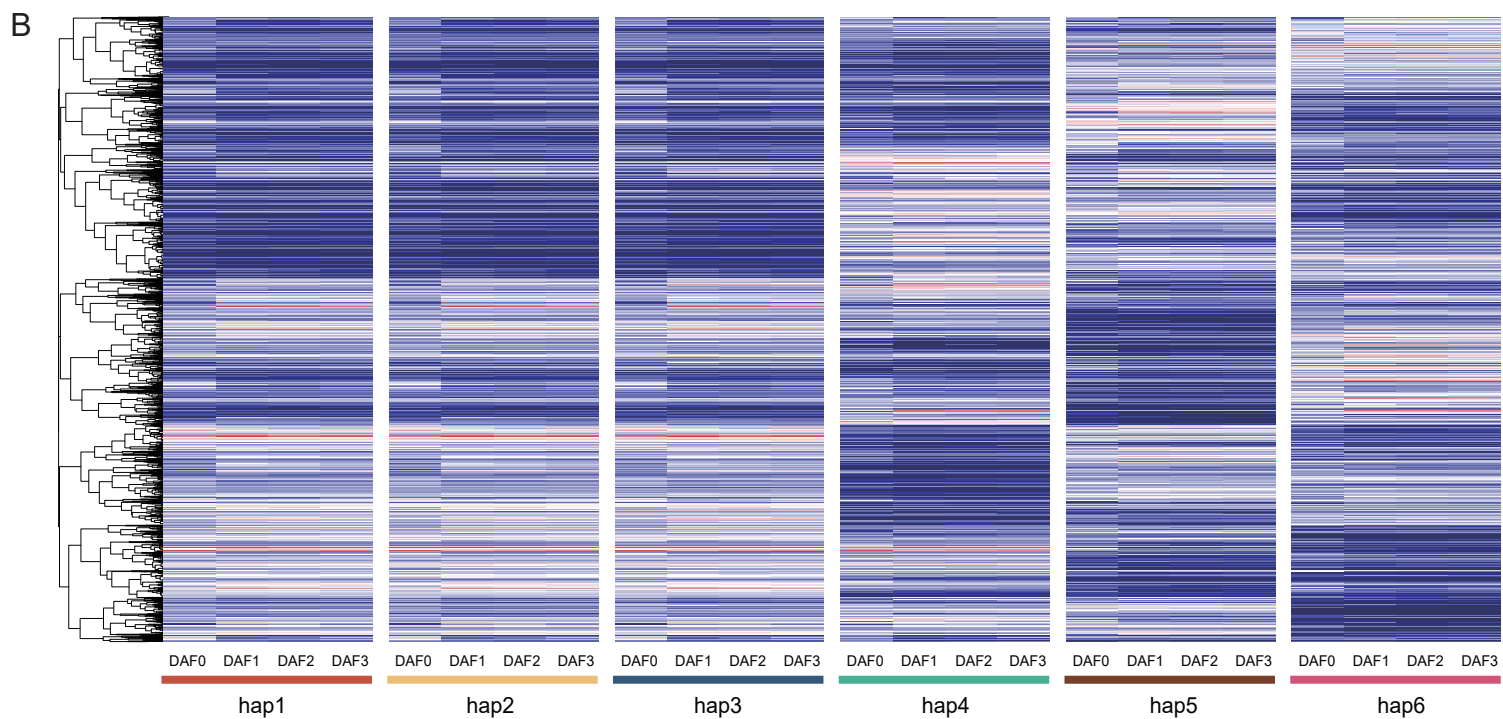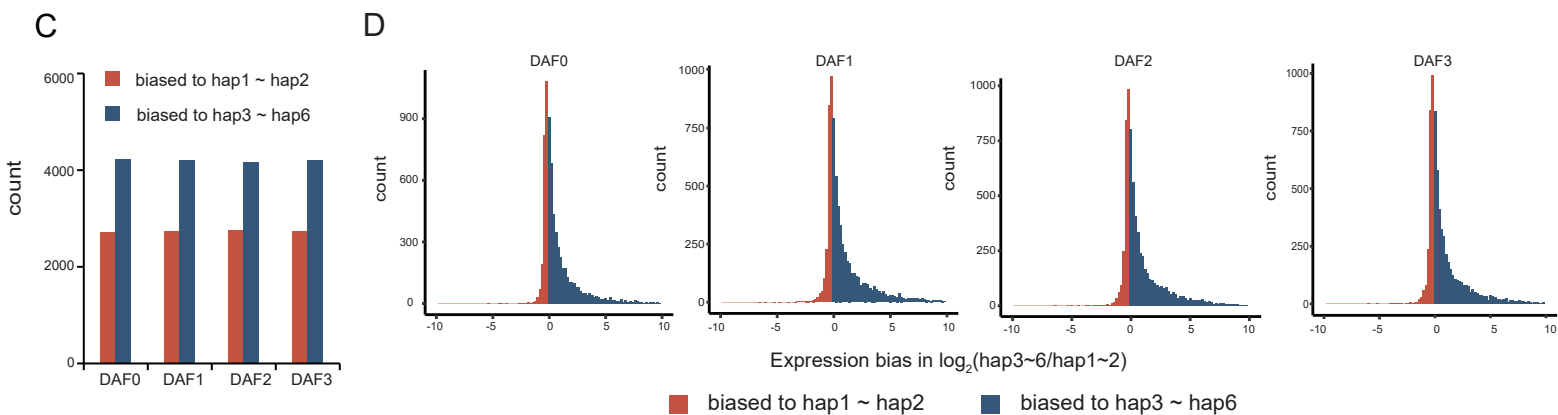

Supplement: Web_Material_uhag011 [file web_material_uhag011.zip › FigureS8.pdf]

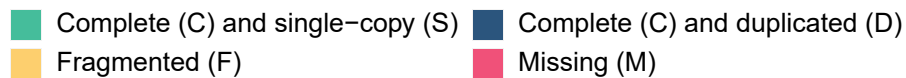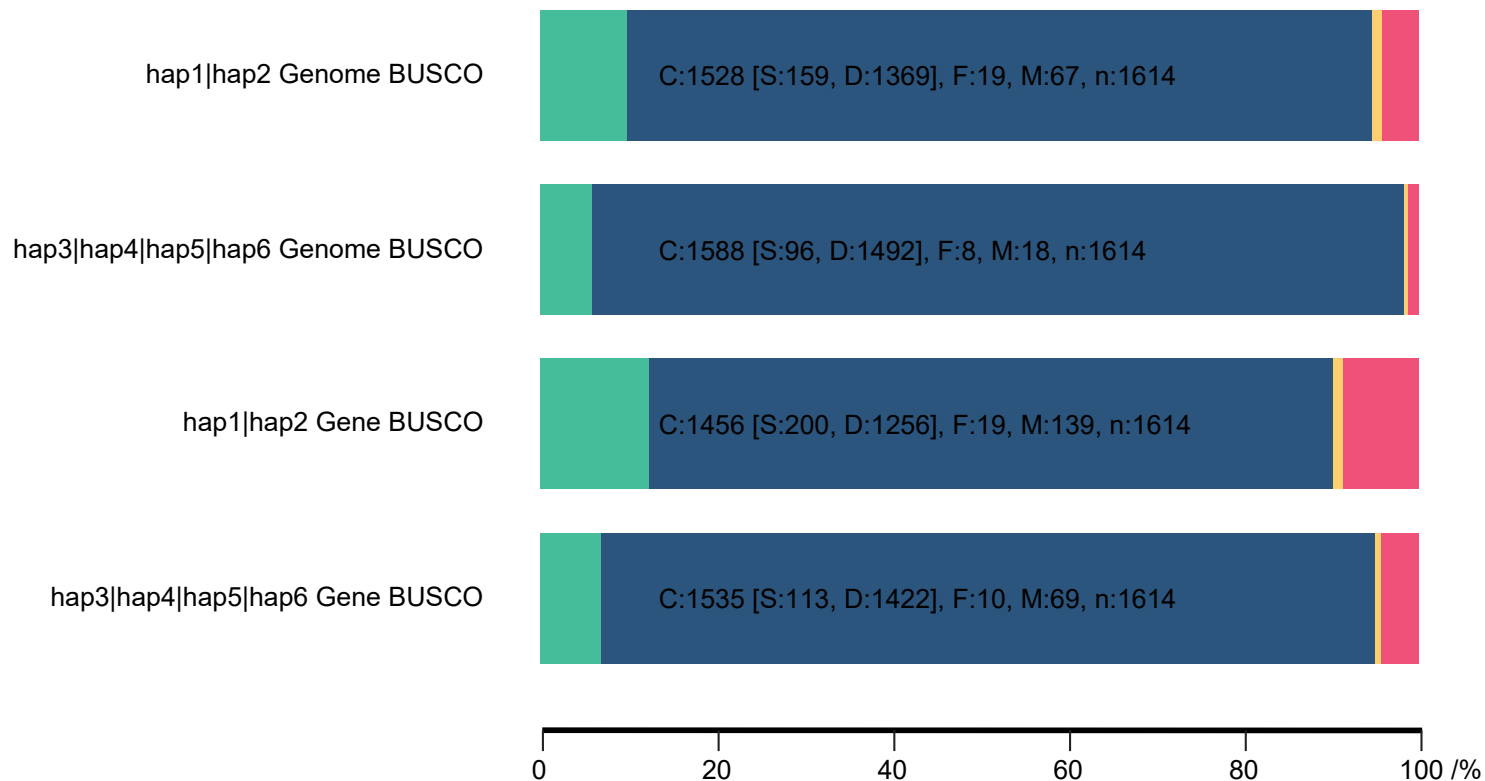

Supplement: Web_Material_uhag011 [file web_material_uhag011.zip › FigureS9.pdf]
